# The potential impact of case-area targeted interventions in response to cholera outbreaks: A modeling study

**DOI:** 10.1371/journal.pmed.1002509

**Published:** 2018-02-27

**Authors:** Flavio Finger, Enrico Bertuzzo, Francisco J. Luquero, Nathan Naibei, Brahima Touré, Maya Allan, Klaudia Porten, Justin Lessler, Andrea Rinaldo, Andrew S. Azman

**Affiliations:** 1 Laboratory of Ecohydrology, École Polytechnique Fédérale de Lausanne, Lausanne, Switzerland; 2 Dipartimento di Scienze Ambientali, Informatica e Statistica, Università Ca’ Foscari Venezia, Venice, Italy; 3 Department of International Health, Johns Hopkins Bloomberg School of Public Health, Baltimore, Maryland, United States of America; 4 Epicentre, Paris, France; 5 Communauté des Amis de l’Informatique pour le Développement–Tchad, N’Djamena, Chad; 6 Department of Epidemiology, Johns Hopkins Bloomberg School of Public Health, Baltimore, Maryland, United States of America; 7 Dipartimento di Ingegneria Civile, Edile ed Ambientale, Università di Padova, Padova, Italy; Mahidol-Oxford Tropical Medicine Research Unit, THAILAND

## Abstract

**Background:**

Cholera prevention and control interventions targeted to neighbors of cholera cases (case-area targeted interventions [CATIs]), including improved water, sanitation, and hygiene, oral cholera vaccine (OCV), and prophylactic antibiotics, may be able to efficiently avert cholera cases and deaths while saving scarce resources during epidemics. Efforts to quickly target interventions to neighbors of cases have been made in recent outbreaks, but little empirical evidence related to the effectiveness, efficiency, or ideal design of this approach exists. Here, we aim to provide practical guidance on how CATIs might be used by exploring key determinants of intervention impact, including the mix of interventions, “ring” size, and timing, in simulated cholera epidemics fit to data from an urban cholera epidemic in Africa.

**Methods and findings:**

We developed a micro-simulation model and calibrated it to both the epidemic curve and the small-scale spatiotemporal clustering pattern of case households from a large 2011 cholera outbreak in N’Djamena, Chad (4,352 reported cases over 232 days), and explored the potential impact of CATIs in simulated epidemics. CATIs were implemented with realistic logistical delays after cases presented for care using different combinations of prophylactic antibiotics, OCV, and/or point-of-use water treatment (POUWT) starting at different points during the epidemics and targeting rings of various radii around incident case households. Our findings suggest that CATIs shorten the duration of epidemics and are more resource-efficient than mass campaigns. OCV was predicted to be the most effective single intervention, followed by POUWT and antibiotics. CATIs with OCV started early in an epidemic focusing on a 100-m radius around case households were estimated to shorten epidemics by 68% (IQR 62% to 72%), with an 81% (IQR 69% to 87%) reduction in cases compared to uncontrolled epidemics. These same targeted interventions with OCV led to a 44-fold (IQR 27 to 78) reduction in the number of people needed to target to avert a single case of cholera, compared to mass campaigns in high-cholera-risk neighborhoods. The optimal radius to target around incident case households differed by intervention type, with antibiotics having an optimal radius of 30 m to 45 m compared to 70 m to 100 m for OCV and POUWT. Adding POUWT or antibiotics to OCV provided only marginal impact and efficiency improvements. Starting CATIs early in an epidemic with OCV and POUWT targeting those within 100 m of an incident case household reduced epidemic durations by 70% (IQR 65% to 75%) and the number of cases by 82% (IQR 71% to 88%) compared to uncontrolled epidemics. CATIs used late in epidemics, even after the peak, were estimated to avert relatively few cases but substantially reduced the number of epidemic days (e.g., by 28% [IQR 15% to 45%] for OCV in a 100-m radius). While this study is based on a rigorous, data-driven approach, the relatively high uncertainty about the ways in which POUWT and antibiotic interventions reduce cholera risk, as well as the heterogeneity in outbreak dynamics from place to place, limits the precision and generalizability of our quantitative estimates.

**Conclusions:**

In this study, we found that CATIs using OCV, antibiotics, and water treatment interventions at an appropriate radius around cases could be an effective and efficient way to fight cholera epidemics. They can provide a complementary and efficient approach to mass intervention campaigns and may prove particularly useful during the initial phase of an outbreak, when there are few cases and few available resources, or in order to shorten the often protracted tails of cholera epidemics.

## Abstract 2

In a modeling study, Andrew Azman and colleagues investigate the potential value of targeted interventions for control of cholera outbreaks.

## Author summary

**Why was this study done?:**

- The risk of cholera around households of cholera cases is higher than in the general population in the days after cholera symptoms start.
- Rapid targeting of cholera interventions to neighbors of cholera cases may provide an effective and resource-efficient way to avert cholera cases and deaths and reduce the duration of epidemics.
- Interventions targeted to neighbors of cases using combinations of antibiotics, oral cholera vaccine, and/or water, sanitation, and hygiene measures have been used in Africa and the Americas to fight cholera, yet limited evidence exists on the potential impact of this approach, the optimal mix of interventions, and the extent of the target population.

**What did the researchers do and find?:**

- Using computational models, we simulated cholera epidemics similar to a large urban cholera outbreak in Chad and evaluated the potential impact of targeted interventions administered to people living within a fixed radius (e.g., 100 m) around reported cholera cases.
- Targeted interventions with oral cholera vaccine were predicted to have the largest impact on reducing cases and shortening epidemics, followed by water treatment interventions and by prophylactic antibiotics, regardless of when interventions started during epidemics.
- The combined use of oral cholera vaccine and water treatment within 100 m around cases starting early in epidemics were estimated to lead to 70% (interquartile range [IQR] 65% to 75%) fewer epidemic days and 82% (IQR 71% to 88%) fewer cases than uncontrolled epidemics.
- Compared to traditional mass intervention campaigns, targeted interventions can have a similar or larger impact on epidemics and use less resources.
- The optimal radius to target around incident case households differed by intervention type, with antibiotics having an optimal radius of 30 m to 45 m compared to 70 m to 100 m for oral cholera vaccine and point-of-use water treatment.

**What do these findings mean?:**

- Interventions targeted to neighbors of cholera cases can be an effective and resource-efficient strategy to fight cholera epidemics; they may be particularly useful during the early phase of an outbreak, when the number of cases is still low, and to truncate the tails of outbreaks, after a mass intervention campaign.
- While field studies and/or clinical trials are needed to measure the effectiveness of targeted interventions, these results provide a rationale to focus efforts on interventions with oral cholera vaccine and water treatment interventions in a roughly 100-m radius around case households.

## Introduction

With over 130,000 cases and 2,400 deaths reported globally in 2016, cholera continues to be a major public health threat, particularly in sub-Saharan Africa [1]. These numbers likely represent an underestimate of the true burden due to poor access to health care, insensitive surveillance systems, and political sensitivities around reporting cases and deaths [2,3]. Cities in sub-Saharan Africa are regularly struck by cholera outbreaks, causing disruption and hindering social and economic development [4,5]. These cities may act as local, national, and/or international hubs of disease spread due to regular travel and migration, and quickly controlling cholera outbreaks in these areas may significantly reduce the number of cholera cases both within the cities and elsewhere.

The cornerstone of cholera prevention and control is improved access to safe water, sanitation, and hygiene (WaSH) and appropriate case management. WaSH includes a heterogeneous mix of interventions, ranging from provision of safe water through infrastructure or point-of-use water treatment (POUWT) tools to latrine building and hygiene behavior change measures [6]. Antibiotics have been used to shorten the duration of shedding in cholera cases and, in some instances, to provide short-term prophylaxis for household contacts, although their prophylactic use is not part of current guidelines by WHO, Médecins Sans Frontières (MSF), the US Centers for Disease Control and Prevention, or icddr,b [7,8]. Recently, oral cholera vaccines (OCVs) have been added to this arsenal and are widely available as a result of the global cholera vaccine stockpile and the addition of new, affordable WHO-prequalified vaccines to the stockpile [9,10]. However, supply of these vaccines remains limited, and countries must often contend with fewer doses than needed to cover the population at risk [11]. OCVs have been shown to be safe, immunogenic, and protective, with 2-dose protection (the standard regimen) lasting at least 3 years, and single-dose protection at least 6 months [12,13], a similar time scale to many cholera epidemics.

These tools are used preventively [14] in areas deemed at high risk for cholera transmission, or reactively in response to a cholera outbreak [15–17]. Control measures are typically given to the population at-large through mass campaigns within high-risk areas, although targeted interventions to households or neighborhoods of cases, including delivery of OCV, antibiotics, and POUWT [7,8,18–20], are common. In Haiti and other countries, efforts to establish rapid response teams tasked with implementing highly targeted interventions are currently underway [21]. The benefits of this type of approach remain unclear, and there is little understanding about when in an epidemic these interventions may have a greater impact than more traditional community-wide interventions, how large an area to target around case households, or the best mix of interventions.

Spatiotemporal clustering of cholera cases—at distances ranging from tens to hundreds of meters—has been observed during numerous cholera outbreaks in endemic and epidemic areas [22–28]. A previous analysis showed that suspected cholera cases were significantly clustered up to distances of at least 200 m from incident case households within the first 5 days of a case presenting for care during epidemics in 2 urban African settings in Chad and the Democratic Republic of the Congo [29]. This clustering has been attributed to common risk factors in those living close to one another, in addition to the risk of transmission often being higher the closer one lives to an infected individual. Intervention strategies targeting disease hotspots [30], particularly vulnerable neighborhoods and camps [31] and other communities, have been successfully applied in the past. Limited literature exists, however, on reactive case-area targeted interventions (CATIs), which take advantage of the inherent spatiotemporal clustering of cholera cases by targeting people living within a given distance around reported cholera cases. Such a strategy could not only present efficient alternatives to reactive mass intervention campaigns in outbreak situations, where resources may be limited or their availability delayed, but may also be used as a complementary approach to mass campaigns when cholera incidence is low, such as during the initial phase or declining tail of an epidemic.

Here we aim to understand the potential impact of CATIs on epidemic cholera using computational transmission models fit to data from a 2011 cholera epidemic in Chad. We aim to provide practical guidance on the best mix of interventions (OCV, POUWT, and/or prophylactic antibiotics), ring size, and timing to maximize efficiency and impact.

## Methods

### Case study and data

During the 2011 cholera epidemic in N’Djamena, Chad, field staff from MSF collected the household coordinates of all suspected cholera cases presenting at the main cholera treatment center starting on June 22 by visiting people at their home (S1 Fig). From August—when the case load began to increase rapidly—through the end of the epidemic in December, household coordinates were collected for every third patient. To minimize potential selection biases, every third patient was identified at the cholera treatment center by an epidemiologist from MSF/Epicentre (NN) who then provided the address to a team of data collectors who visited each household. The resulting dataset, combining the overall epidemic curve of suspected cholera cases (citywide) with GPS coordinates of patient’s homes, has been described previously [29] (S2 Fig). The epidemic totaled 4,352 reported cases (within a population of 993,500) and lasted for 232 days. The attack rate varied between 11.6 and 59.6 per 10,000 among the 10 districts (arrondissements) of the city (S2 Fig). As these data were originally collected for operational purposes, this study did not have a prospective protocol, and it was deemed to be exempt research by the Johns Hopkins Bloomberg School of Public Health Institutional Review Board.

### Quantification of spatiotemporal clustering

To quantify the spatiotemporal clustering of cholera cases, we used the τ statistic, a measure of the relative risk that a person living at a given distance from a known cholera case also becomes a case compared to any person in the entire population becoming a case within the same time frame [32–36] (S1 Text). In the presence of spatiotemporal clustering at a particular distance and time, τ is greater than 1.

### Epidemiological model

**Fig 1:**
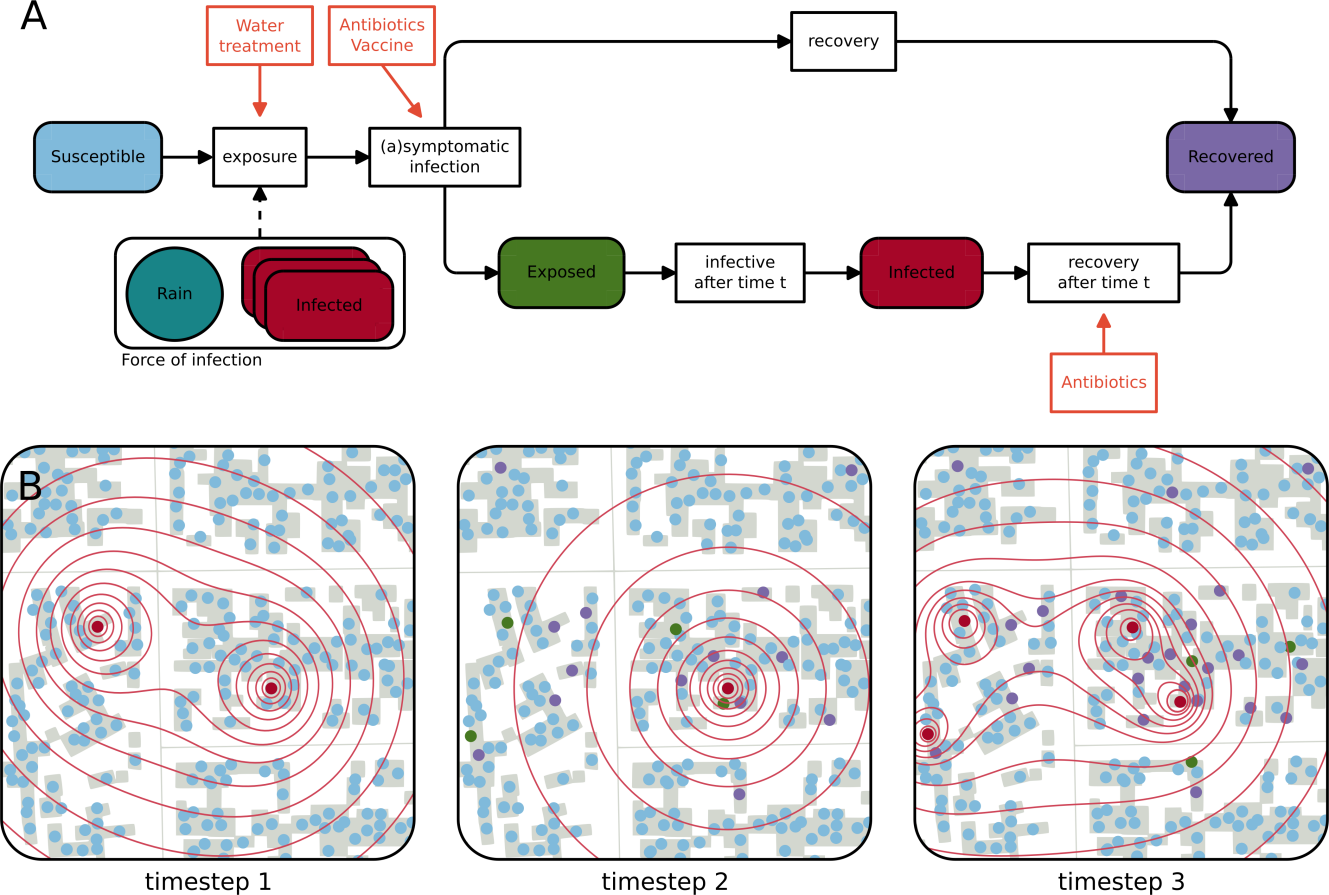
Schematic representation of the epidemiological model and evolution of the infectious state of inhabitants of a neighborhood. (A) Flow chart of the model representing the different epidemiological states a person can be in and the processes that lead to a change of state. The force of infection acting on a susceptible individual depends on the number of infected individuals and the distance to each of them as well as on rainfall during the last 10 days. Orange boxes represent pathways through which interventions (antibiotics, oral cholera vaccine, and point-of-use water treatment) influence the processes in the model. (B) Schematic representation of the evolution of the epidemiological state of the inhabitants of a neighborhood in N’Djamena during 3 timesteps. The closer susceptible people (blue dots) live to an infected individual (red dots), the higher the force of infection (red contours) they face. Susceptible individuals can get symptomatically infected, which means that they get exposed (green dots) and go on to become infectious after their incubation period (red dots), and thus contribute to the force of infection, or asymptomatically infected, in which case they are assumed to recover (purple dots). Infected individuals recover after a given duration. Between timesteps 1 and 2, 1 infected person recovered, 4 susceptible individuals got exposed, and 14 susceptible individuals contracted an asymptomatic infection. At timestep 3, the individuals infected at timestep 1 have recovered, and all exposed individuals have become symptomatic.

We developed an individual-based, spatially explicit stochastic model (S1 Text) and calibrated it to the 2011 cholera outbreak in N’Djamena. All 993,500 inhabitants of the city were assigned a geographical location according to the population density estimated using remotely sensed built-up density as a proxy [37,38] (S2 Fig). Demographic processes, like births and deaths, were assumed to be negligible during the short time course of the outbreak. In the model, each individual’s state (e.g., susceptible, exposed, infectious, or recovered) is tracked during the outbreak (Fig 1A). Susceptible individuals are exposed to a spatially distributed force of infection originating from infectious individuals and decreasing with distance (Fig 1B). The force of infection is modulated by rainfall, which has been shown to be an important environmental driver of cholera epidemics in several settings [39–43]. Exposed individuals can become either symptomatically infected, after an incubation period with a mean duration of 2 days [44], or mildly/asymptomatically infected, in which case it is assumed that they do not significantly contribute to the force of infection [45,46]. Symptomatic infection lasts for an average of 5 days before individuals recover [47].

**Fig 2:**
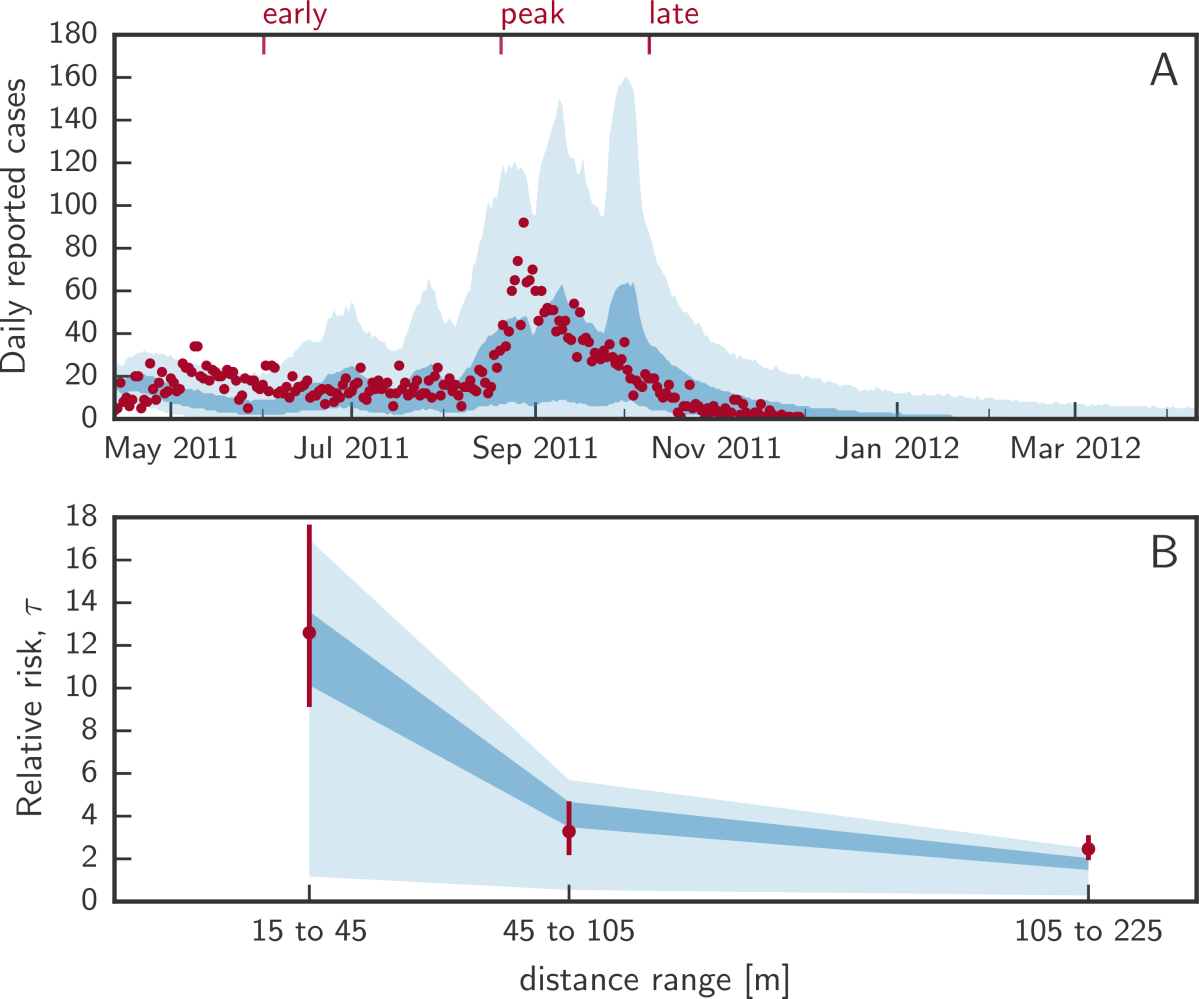
Calibrated model fit. (A) shows the distribution of daily incident cholera cases from uncontrolled epidemic simulations. The shaded areas represent the marginal interquartile range (dark blue) and the 2.5th and 97.5th percentiles (light blue) from 1,000 simulated epidemics with the true number of daily reported cases shown as red dots. Red ticks at the top represent the 3 times when interventions start. (B) shows the interquartile range (dark blue) and 2.5th and 97.5th posterior percentiles (light blue) of the relative risk (τ statistic) of the next case being within a specific distance from a case within 5 days of his/her symptom onset. Red dots and bars (95% confidence intervals) represent the computed τ from the data.

The 4 free parameters of our model are the ratio of symptomatic to asymptomatic infections, a kernel-independent transmission rate, a shape parameter of the power-law transmission kernel, and a coefficient governing the influence of rainfall. Model calibration was performed using an approximate Bayesian computation population Monte Carlo (ABC-PMC) approach (S1 Text) [48]. Specifically, we calibrated the model to the number of newly reported cases per day (i.e., the epidemic curve; Fig 2A) and the spatiotemporal clustering of the case households, as captured by the τ statistic. We estimated τ at 3 different representative distance windows (15 to 45 m, 45 to 105 m, and 105 to 225 m)—chosen to fit the spatial discretization of the model domain—and focused on cases occurring within 5 days after each case (Fig 2B). For simplicity and interpretability, we used the sum of squared errors as a goodness of fit measure for both criteria. The calibration was run with 512 particles, which were accepted if the sums of squared errors of both criteria were lower than predefined thresholds adapted after every calibration step (S1 Text).

### Intervention strategies

To evaluate the benefits (e.g., averted symptomatic cases) and resource needs (e.g., number of people targeted and number of clusters targeted through CATI) of different types of interventions, we simulated a total of 111 scenarios (and several sensitivity analyses), combining different intervention types, modes of allocation, and intervention starting times (S1 Table). Out of an initial 1,000 epidemics simulated without interventions, the ones with at least 10 new cholera cases during the week preceding the initiation time (833, 836, and 829 for interventions starting on day 50, 130, and 180 of the epidemic, respectively) were resimulated for every intervention scenario. This threshold of 10 was selected through trial and error to ensure that simulated epidemics reflected those where interventions were likely to be put in place (e.g., in epidemics with clear evidence of ongoing transmission) and to reduce the number of comparisons where the differences between uncontrolled and intervention simulations was largely a result of stochastic effects rather than the interventions. Simulations were run up to 1 year from the first cases to stay within the realm of historic outbreak durations. For each intervention scenario we then computed the median and interquartile range for the number of averted cases and the number of epidemic days reduced, over all simulations. These interquartile ranges (or prediction intervals) capture the uncertainty related to the model parameters (intervention- and disease-transmission-related parameters) and stochastic variations. Note that, using this method, some simulations may generate a higher number of cases and/or a longer epidemic with interventions than without, because the course of the simulated epidemic trajectories is subject to stochastic processes.

We evaluated 3 types of interventions both individually and in combination: the administration of a single dose of antibiotics (e.g., azithromycin), the administration of a single dose of OCV, and a POUWT intervention (Fig 1A). We reconstructed probability distributions (S14 Fig) for each of the intervention effects, with mean effect sizes and measures of variability derived from the literature (Table 1), and drew from these distributions in simulations. A detailed description of the effects of the 3 types of interventions and their implementation is given in S1 Text. Antibiotics and OCV were assumed to reduce an individual’s probability of becoming symptomatic if infected [49,50]. In addition, we assumed that antibiotics reduce a symptomatic individual’s infectious period [51]. Given the limited cholera-specific effectiveness data for POUWT (or any WaSH intervention [52]), we based our estimate of effectiveness on a meta-analysis of POUWT interventions in urban/peri-urban areas on all cause diarrhea [6]. We assumed that POUWT leads to a 26% reduction in exposure to *Vibrio cholerae* and thus reduces the probability of getting infected [6]. While we assumed that antibiotics and POUWT provide immediate protection, we considered OCV to be fully effective after a lag of 7 days (with 0 efficacy before), based on the design of a clinical trial assessing its efficacy [50]. The effects of antibiotics were assumed to last for 2 days [53], whereas protection from OCV and POUWT was assumed to last through the end of each epidemic. In our main analyses, a person can receive prophylactic antibiotics only once during the entire epidemic even if they live within overlapping targeted areas at different times. In secondary analyses, we included a scenario where a person is eligible for antibiotics each time he/she is in a targeted cluster, provided there has been at least a 2-week gap since the last time he/she was targeted with antibiotics (S1 Text).

**Table 1:** Effects of the 3 types of interventions as implemented in the model.

Measure	Intervention		
	Antibiotics	Oral cholera vaccine	Point-of-use water treatment
Relative risk of symptomatic infection^a^	0.045 (0.001, 0.296)^c^	0.37 (0.18, 0.76)^c^	—
Relative risk of exposure^a^	—	—	0.74 (0.65, 0.85)^c^
Reduction of the infectious period (days)^b^	2.74 (2.40, 3.07)^c^	—	—
Lag to onset of the effect (days)	0	7	0
Duration of the effect	2 days	Full epidemic	Full epidemic
References	[8,49,51,53,54]	[50]	[6]

All relative measures are comparing those receiving the intervention to those not receiving the intervention.

^a^Relative reductions of symptomatic fraction and exposure with respect to no intervention are multiplied by the corresponding model parameter (σ and β, respectively). One minus the relative reduction can be interpreted as the efficacy of the intervention.

^b^The reduction of the infectious period is subtracted from the value without intervention.

^c^95% confidence intervals are given in parentheses.

In our model, CATIs are implemented by targeting people within a given radius (15, 30, 45, 70, or 100 m; S13 Fig) around each reported (i.e., symptomatic) case. In N’Djamena, rings of those radii contain an average (range) of 9 (2 to 21), 42 (5 to 76), 75 (10 to 120), 167 (14 to 263), and 295 (55 to 456) people, respectively (S12 Fig).

To account for the fact that an intervention team visiting a target cluster would not be able to reach all inhabitants because they might be absent, be unwilling to receive the intervention, not comply with interventions, or be under 1 year of age (minimum age for antibiotics and OCV), we assumed that a random sample of 70% of the target population can be effectively reached [18]. We accounted for a variable delay between the onset of symptoms of the initial case and the deployment of an intervention team to the target area based on data from South Sudan and other settings [18]; the delay was drawn from a distribution ranging from 0 to 7 days with a mode of 2 (S16 Fig). Of note, our mechanistic modeling approach implicitly accounts for the fact that with longer intervention delays, the proportion of case neighbors already immune to cholera increases.

To understand the relative value of CATIs, we compared their impact and efficiency with (1) small mass campaigns within the entire city, assuming that the same total number of people gets targeted, (2) mass intervention campaigns reaching 70% of the city population, and (3) mass intervention campaigns targeted to the 3 districts with the highest attack rate at the start of the campaign with 70% coverage in each (S1 Text). To provide an estimate of the logistical implications, we also computed the number of rings that were targeted per day with different CATIs.

As timing is key in controlling outbreaks, we considered 3 different scenarios for the start of interventions (CATIs and mass campaigns). Interventions started during the early (stable) phase in the epidemic (day 50 of the epidemic, May 31), around the peak of the epidemic (day 130, August 19), or late, as the epidemic is declining after the peak (day 180, October 8) (Fig 2). Whereas randomly allocated interventions and mass campaigns were presumed to take 2 weeks to complete (with the same number of interventions administered every day), we assumed that CATIs continue until the end of the simulated epidemic (e.g., no more exposed nor infected individuals in the model) or until the maximum simulation time of 1 year.

## Results

### Calibration

The calibrated model reproduced key characteristics of the 2011 epidemic in N’Djamena, including the epidemic curve and the spatiotemporal clustering of cases (τ; Fig 2). In simulated uncontrolled epidemics, a median of 3,381 (IQR 1,535 to 5,811) cases occurred, with epidemics lasting a median of 262 (IQR 218 to 311) days. In the calibrated model, individuals living within 15 m to 45 m of a cholera case had a 12.1-fold (IQR 10.1 to 13.9) greater risk than the general population of becoming a cholera case within 5 days of the primary case developing symptoms (Fig 2B). The posterior distributions of fitted parameters are shown in S3 Fig.

### Individual interventions

**Fig 3:**
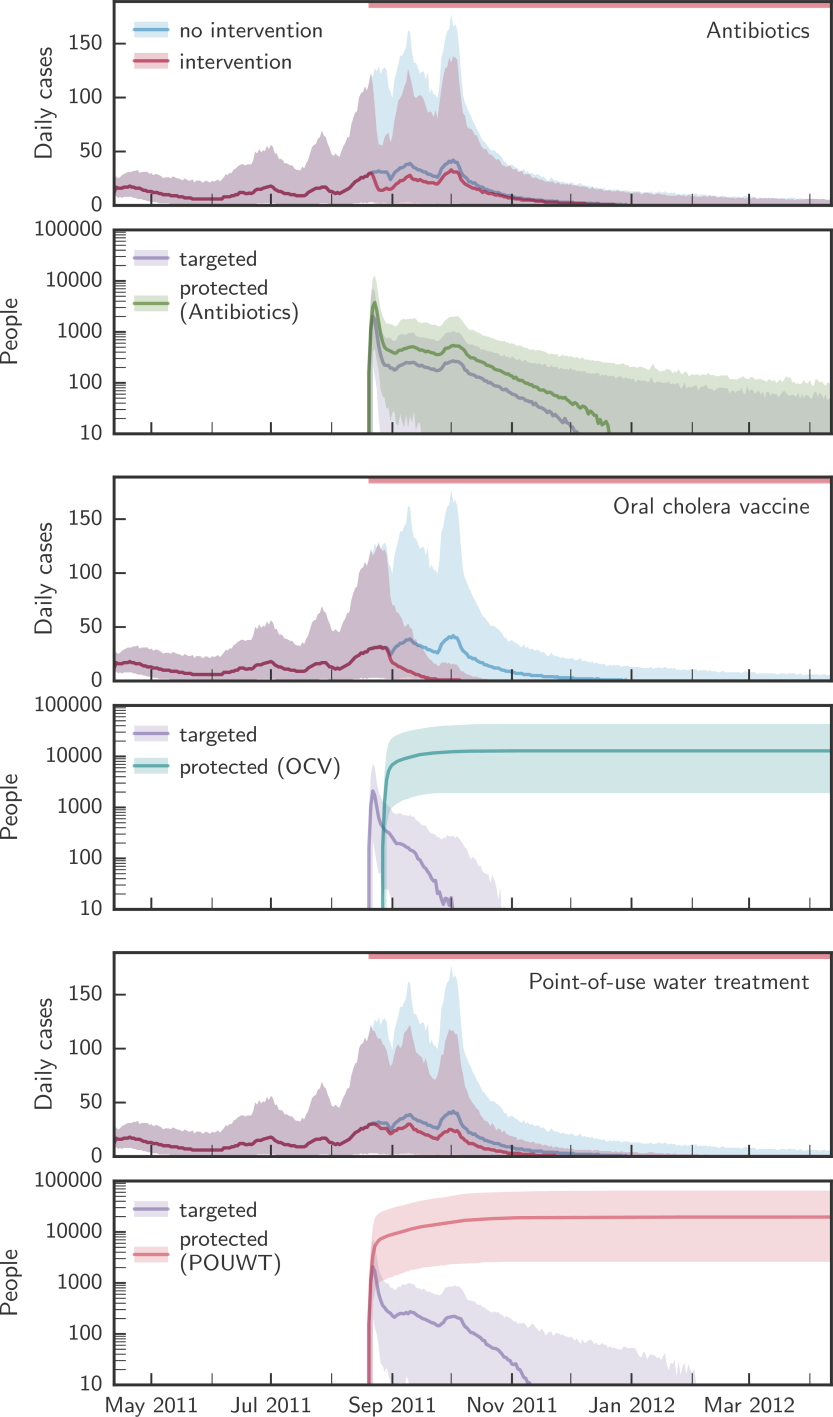
Comparison of the simulated evolution of epidemics with and without case-area targeted interventions. Upper panels in each pair of panels show the simulated evolution of the epidemics without intervention and with case-area targeted allocation of antibiotics, OCV, or POUWT within a 100-m radius starting at the epidemic peak. Lower panels in each pair of panels show the corresponding number of people targeted daily and the number of people protected by each intervention. Solid lines designate the median over all simulations, shaded areas the 2.5th and 97.5th percentiles. The red bars at the top of the panels mark the period during which interventions were applied. OCV, oral cholera vaccine; POUWT, point-of-use water treatment.

Each of the primary interventions—POUWT, OCV, and antibiotics—rapidly decreased incidence when targeted to individuals living within 100 m of a case (Fig 3) and reduced the duration of epidemics. Antibiotics led to the sharpest short-term reduction in incidence due to the high degree of short-term protection. However, the rate of incidence reduction was not sustained due to the short-lasting protection of antibiotics.

**Fig 4:**
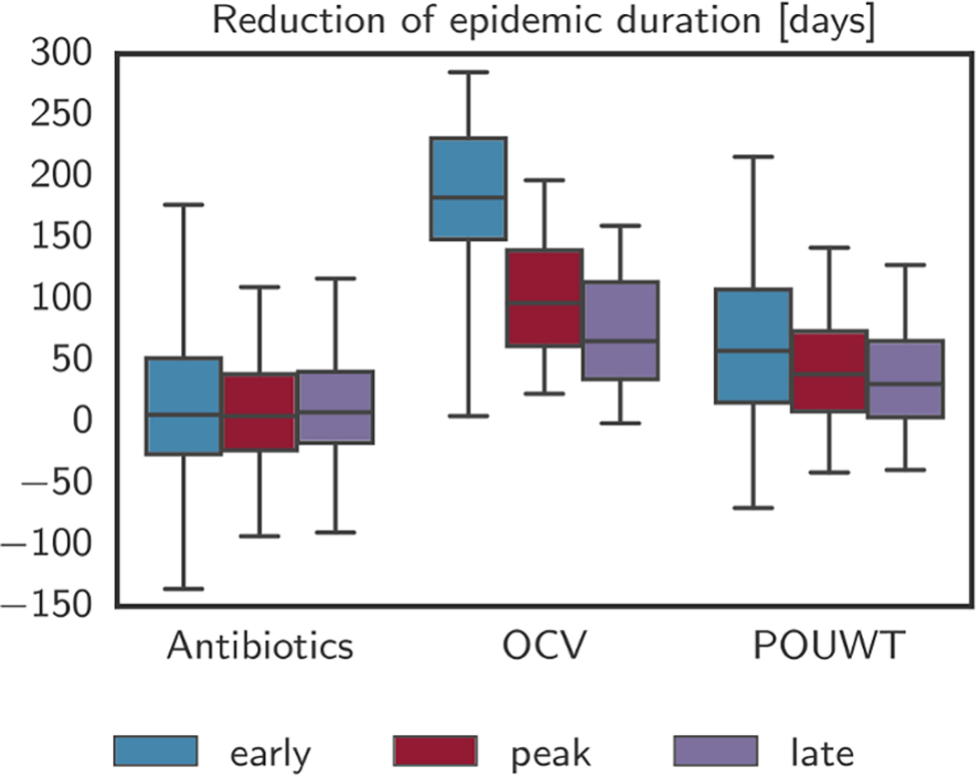
Reduction of epidemic duration with case-area targeted interventions. Reduction of epidemic duration predicted by the model for the 3 main intervention types with case-area targeted allocation in a 100-m radius starting at 3 different times. Whiskers mark the 2.5th and 97.5th percentiles. Negative numbers of days, such as visible for antibiotics, are due to stochastic effects that arise when an intervention alters the course of a particular epidemic without halting it and leads to a higher number of cases at a later point in time. OCV, oral cholera vaccine; POUWT, point-of-use water treatment.

POUWT and OCV led to faster extinction of outbreaks than antibiotics (Fig 4; Table 2). When interventions started early (day 50; Fig 2), OCV reduced epidemic durations by 68% (IQR 62% to 72%), POUWT by 21% (IQR 7% to 35%), and antibiotics by 2% (IQR −11% to 8%). When interventions started around the peak (day 130), epidemics were shortened by 35% (IQR 26% to 44%) using OCV, by 15% (IQR 4% to 24%) using POUWT, and by 2% (IQR −9% to 14%) using antibiotics. Even when intervening late in epidemics (day 180), each of the interventions truncated the epidemic, with a 24% (IQR 14% to 35%) reduction from OCV, 11% (IQR 2% to 21%) from POUWT, and 3% (IQR −7% to 14%) from antibiotics. We found similar qualitative results when interventions were targeted to different radii around incident cholera cases (S1 Table).

**Table 2:** Cases averted and reduction in epidemic duration from different case-area targeted interventions with ring size of 100 m starting early, around the peak, and late in the epidemic (median (IQR)).

Intervention	Reduction in epidemic duration (%)			Averted cases (%)		
	Early	Peak	Late	Early	Peak	Late
Antibiotics	2 (−11, 18)	2 (−9, 14)	3 (−7, 14)	24 (0, 45)	14 (6, 21)	5 (1, 9)
OCV	68 (62, 72)	35 (26, 44)	24 (14, 35)	81 (69, 87)	43 (35, 49)	8 (4, 13)
POUWT	21 (7, 35)	15 (4, 24)	11 (2, 21)	51 (33, 64)	20 (14, 27)	5 (2, 10)
Antibiotics and OCV	70 (64, 74)	37 (28, 46)	25 (16, 36)	83 (72, 88)	50 (41, 55)	10 (6, 16)
Antibiotics and POUWT	26 (11, 45)	15 (5, 25)	15 (5, 23)	61 (45, 72)	30 (24, 36)	8 (5, 13)
OCV and POUWT	70 (65, 75)	38 (29, 47)	25 (16, 36)	82 (71, 88)	47 (38, 52)	9 (5, 14)
Antibiotics, OCV, and POUWT	72 (67, 76)	39 (30, 48)	26 (17, 37)	83 (72, 89)	51 (42, 57)	11 (7, 16)

OCV, oral cholera vaccine; POUWT, point-of-use water treatment.

**Fig 5:**
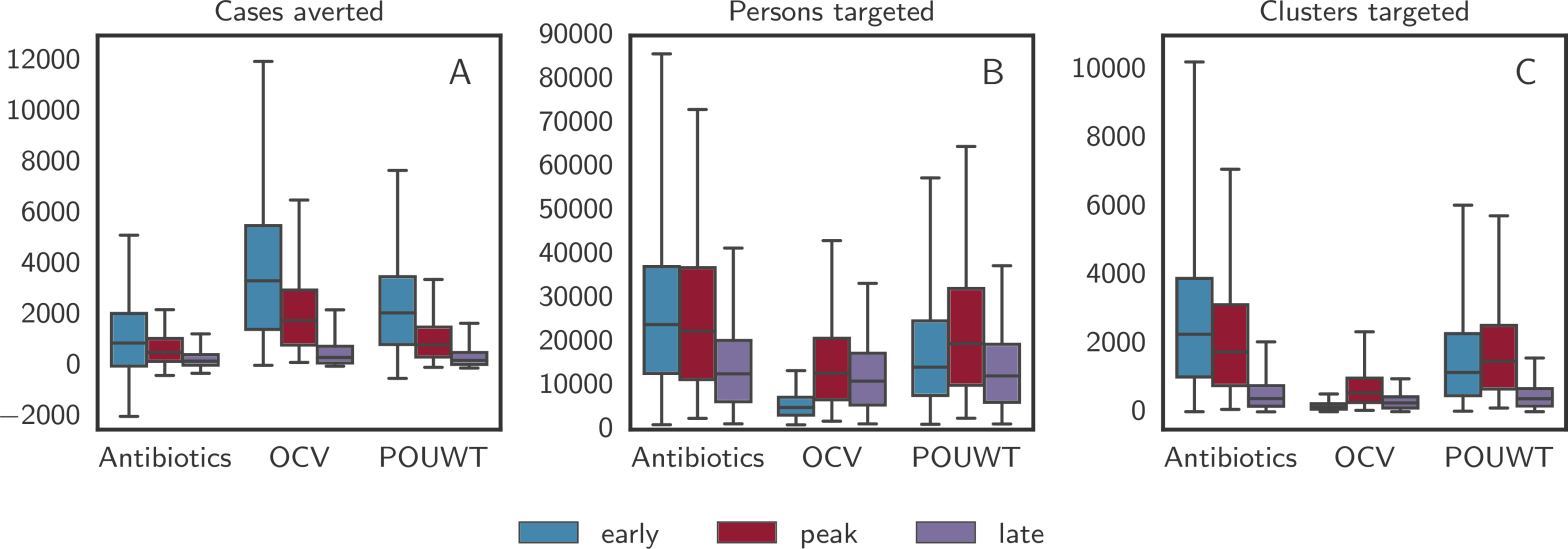
Outcome of the 3 main interventions with case-area targeted allocation in a 100-m radius. Boxplots of (A) the number of averted cases, (B) the number of targeted persons, and (C) the number of targeted clusters predicted by the model for the 3 main intervention types with case-area targeted allocation in a 100-m radius starting at 3 different times. Whiskers mark the 2.5th and 97.5th percentiles. Negative numbers of averted cases, such as those given for antibiotics, are due to stochastic effects that arise when an intervention alters the course of a particular epidemic without halting it and leads to a higher number of cases at a later point in time. OCV, oral cholera vaccine; POUWT, point-of-use water treatment.

The number of cases averted by CATIs focused within 100 m from cases varied and depended on the timing and type of intervention (Table 2; Fig 5A). When interventions started close to the peak of the epidemic, the reduction in cases compared to uncontrolled epidemics for CATIs within 100 m was 43% (IQR 35% to 49%) with OCV alone, 20% (IQR 14% to 27%) with POUWT alone, and 14% (IQR 6% to 21%) with antibiotics alone. Regardless of the intervention type, more cases were averted when interventions were initiated earlier in the epidemic. Interventions that averted more cases in a shorter period of time reduced the duration of epidemics and ultimately required less resources (i.e., number of people and clusters targeted) (Fig 5B and 5C).

### Combined interventions

**Fig 6:**
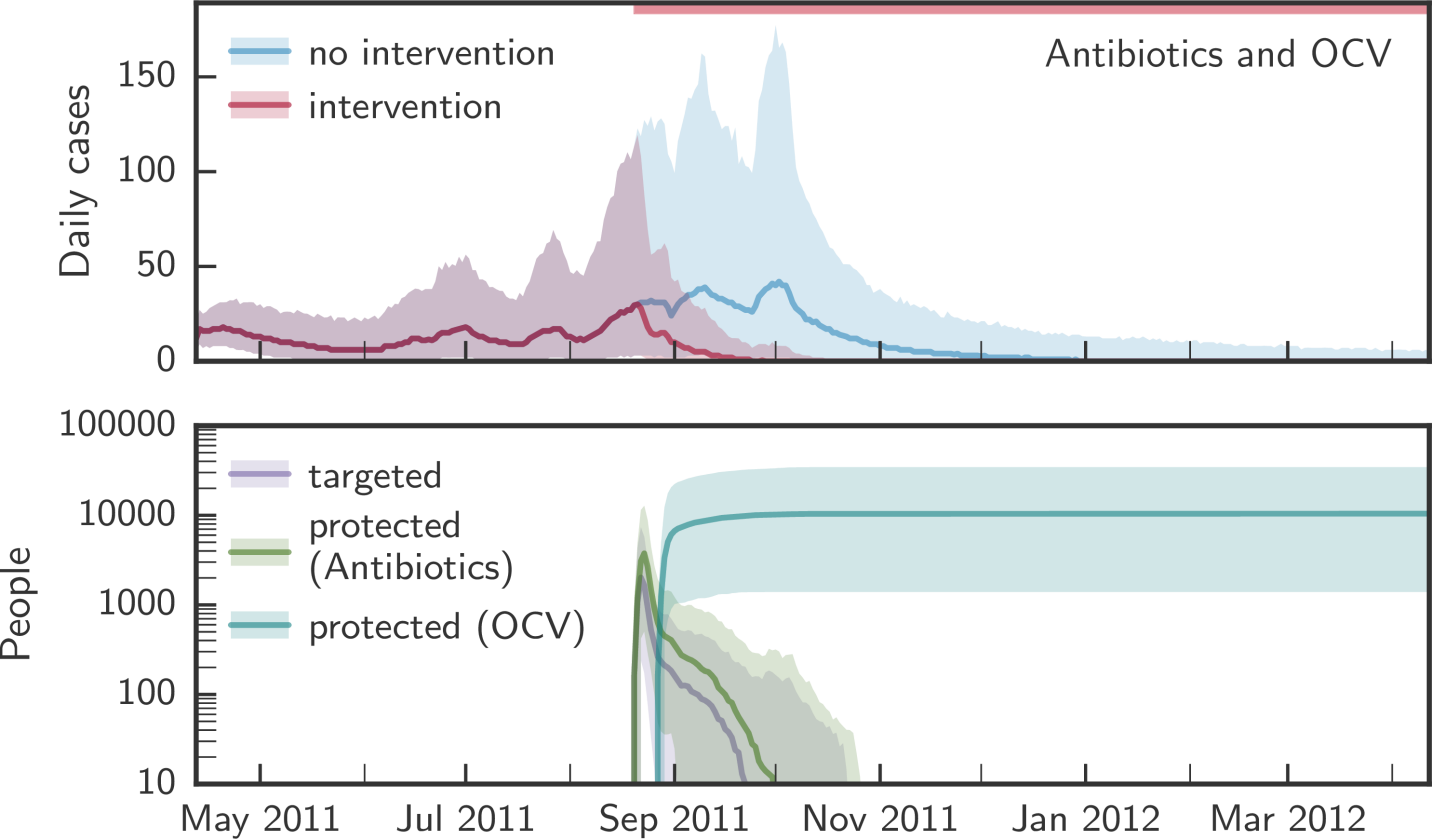
CATIs within a radius of 100 m combining antibiotics and OCV. The upper panel shows the simulated evolution of the epidemics without intervention (blue) and with simultaneous CATIs (red) using antibiotics and OCV within a 100-m radius starting around the epidemic peak. The lower panel shows the number of people targeted each day (purple), and the number of people protected by antibiotics (green) and OCV (blue). Solid lines show the median over all simulations; shaded areas represent the 2.5th and 97.5th percentiles. The red bar at the top of the figure marks the period during which the intervention was applied. CATI, case-area targeted intervention; OCV, oral cholera vaccine; POUWT, point-of-use water treatment.

Combinations of the different interventions, each with its own mechanism of protection, led to larger, quicker, and more robust impacts on the epidemic (Table 2). CATIs with OCV and antibiotics in a radius of 100 m from case households led to 70% (IQR 64% to 74%) shorter epidemics when started early, 37% (IQR 28% to 46%) shorter epidemics when started around the peak, and 25% (IQR 16% to 36%) shorter epidemics when started late (Fig 6). When combining OCV and POUWT in a 100-m radius, epidemics were shortened by 70% (IQR 65% to 75%), 38% (IQR 29% to 47%), and 25% (IQR 16% to 36%) when starting interventions early, around the peak, and late, respectively. CATIs with antibiotics and POUWT reduced epidemic durations by 26% (IQR 11% to 45%), 15% (IQR 5% to 25%), and 15% (IQR 5% to 23%) starting early, around the peak, and late, respectively.

Interventions in a radius of 100 m combining OCV with antibiotics and/or POUWT led to a similar number of cases averted. CATIs starting early during the epidemics reduced the cases by 83% (IQR 72% to 88%) using OCV and antibiotics, by 82% (IQR 71% to 88%) using OCV and POUWT, by 8% (IQR 5% to 13%) using POUWT and antibiotics, and by 83% (IQR 72% to 89%) when using all 3 types of intervention. When starting at the epidemic peak, OCV and antibiotics led to a 50% (IQR 41% to 55%) reduction in cases, with OCV and POUWT leading to a 47% (IQR 38% to 52%) reduction, antibiotics and POUWT to a 30% (IQR 24% to 36%) reduction, and all 3 types of intervention to a 51% (IQR 42% to 57%) reduction. Starting late during the epidemic, a combination of OCV and antibiotics resulted in 10% (IQR 6% to 16%) fewer cases, OCV and POUWT in 9% (IQR 5% to 14%) fewer cases, POUWT and antibiotics in 8% (IQR 5% to 13%) fewer cases, and all 3 interventions in 11% (IQR 7% to 16%) fewer cases.

### Intervention ring size

**Fig 7:**
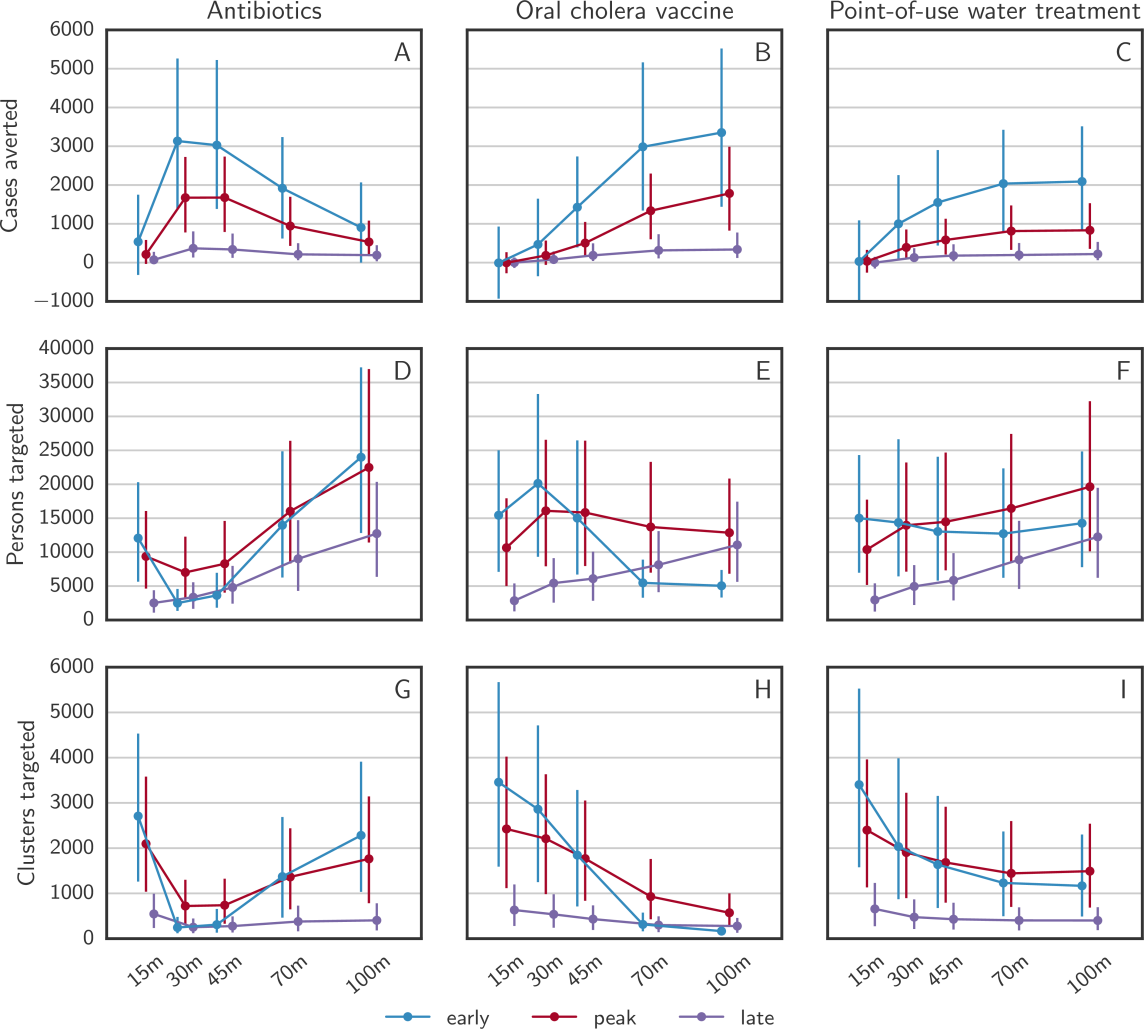
Intervention outcomes as a function of distance in case-area targeted allocations. The numbers of (A–C) averted cases, (D–F) targeted persons, and (G–I) targeted clusters predicted by the model for the 3 main intervention types with case-area targeted allocation and variable radius, starting at 3 different times. The error bars cover the range between the 25th and the 75th quantile over all simulations.

For OCV and POUWT, the number of averted cases steadily increased with the CATI radius until 70 to 100 m. For CATIs with antibiotics, the curves of averted cases by ring radius peaked at 30 m, roughly 2 to 3 times as high as at 100 m, and similar to OCV at 100 m (Fig 7A–7C). This effect results from the short-lasting protection from antibiotics in combination with the limitation that every person can only be targeted once. The epidemic wave arrives at distances farther from the primary case after the protective effect from antibiotics has already vanished. For all 3 types of intervention, the ring size that led to the most efficient reduction in cases was similar regardless of the intervention timing. However, the number of clusters targeted decreased with increasing ability to rapidly stop epidemics (Fig 7G–7I). The number of persons targeted over different radii was governed by 2 contrasting effects, a decrease with better performing interventions and an increase with larger cluster radii (Fig 7D–7F).

### Efficiency of CATIs and comparison to mass intervention campaigns

**Fig 8:**
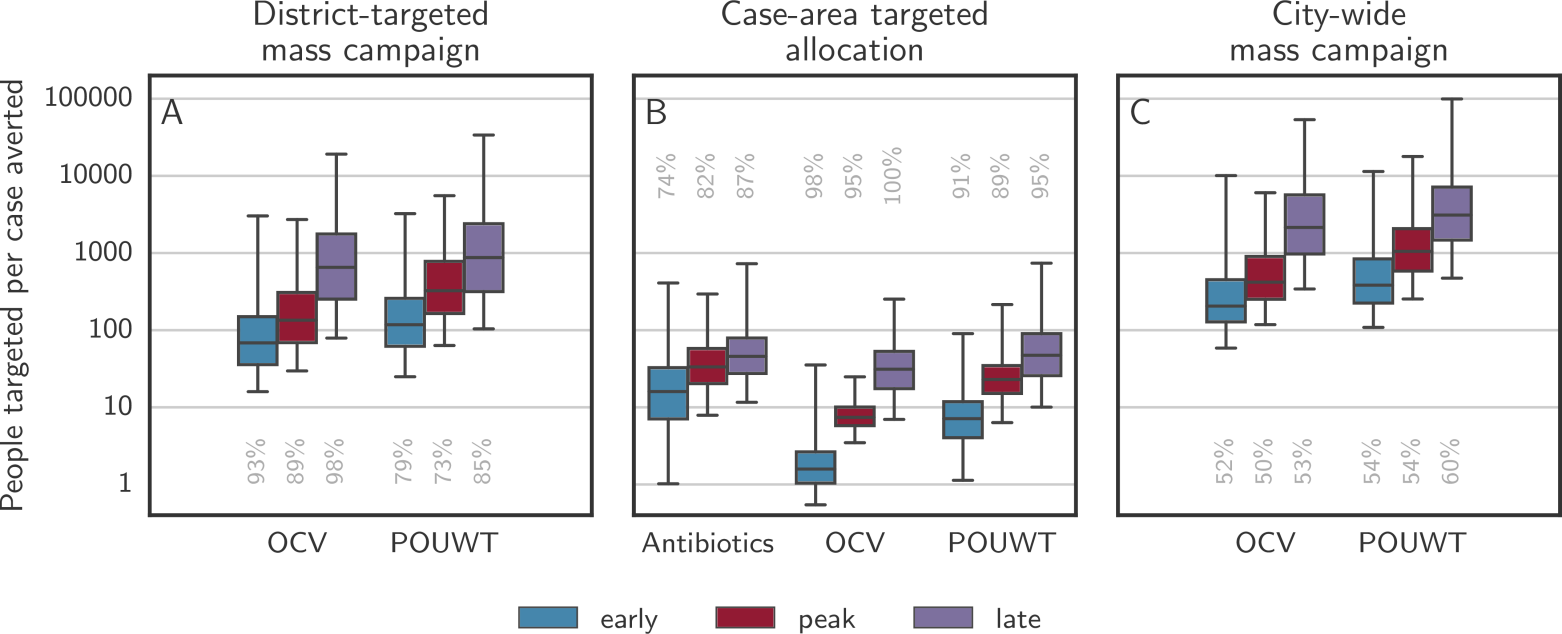
Persons needed to target per case averted for district-targeted mass campaigns, CATIs, and city-wide mass campaigns. Boxplots show the number of persons needed to target to avert 1 case when using antibiotics, OCV, and POUWT using different allocation approaches. (A) illustrates campaigns targeting 70% of the population of the 3 districts with the highest attack rate at the time of intervention. Mass allocation of antibiotics was not considered as it is unlikely to be a realistic approach. (B) illustrates CATIs at a radius of 100 m. (C) illustrates campaigns targeting 70% of the population of the entire city. Whiskers mark the 2.5th and 97.5th percentiles. Only model runs with a positive number of cases averted were considered, with the grey numbers aligned with each box illustrating the percentage of such runs among all simulations. CATI, case-area targeted intervention; OCV, oral cholera vaccine; POUWT, point-of-use water treatment.

The most efficient type of CATI in a radius of 100 m was OCV, with 1.6 (IQR 1.0 to 2.6) people targeted per case averted when starting interventions early, 7.4 (IQR 5.8 to 10) when starting around the peak, and 31 (IQR 17 to 53) when starting late (Fig 8B). For POUWT, the number of people targeted per case averted was 7.1 (IQR 4.0 to 12) when starting interventions early, 23 (IQR 15 to 35) when starting around the peak, and 47 (IQR 26 to 90) when starting late. For antibiotics, it was 16 (IQR 7.1 to 33) when starting early, 33 (IQR 20 to 58) when starting around the peak, and 46 (IQR 27 to 79) when starting late.

Mass intervention strategies, where a large proportion (i.e., 70% in our case, corresponding to approximately 700,000 people) of the city population was targeted in a short time period, achieved similar numbers of averted cases (S6 Fig), but typically required hundreds to tens of thousands of people to be targeted in order to avert a single case. For an intervention campaign starting around the epidemic peak, CATIs within a radius of 100 m were 58 times (IQR 36 to 112) more efficient than a mass intervention campaign using OCV and 43 times (IQR 25 to 85) more efficient using POUWT.

To gauge the relative value of spatial targeting of interventions, we simulated small mass campaigns allocating the same number of doses as used by CATIs throughout the entire city. This mode of intervention did not effectively stop epidemics nor reliably avert significant numbers of cases (S7 Fig), with almost 50% of the simulated epidemics with interventions showing no improvement. When taking into account only simulated epidemics with a positive number of averted cases, CATIs starting around the epidemic peak in a 100-m ring around a primary case led to a 6-fold (IQR 3 to 11) higher reduction in cases using OCV and a 3-fold (IQR 2 to 7) higher reduction using POUWT than their non-targeted counterparts.

As mass interventions are not often applied to an entire city (due to limited resources, especially of OCV [11]), we simulated mass campaigns targeting 70% of the population of the 3 (out of 10) districts with the highest attack rate at the time of intervention (district-targeted campaigns). The number of cases averted using this approach was similar to that achieved with CATIs, but the number of people targeted was considerably higher (up to 300,000 people; S6 Fig) and epidemics typically lasted longer. District-targeted campaigns with OCV shortened epidemics by 37% (IQR 12% to 61%) when started early, by 21% (IQR 11% to 32%) when started at the epidemic peak, and by 15% (IQR 4% to 25%) when started late. In campaigns with POUWT alone, epidemics were shortened by 9% (IQR −5% to 24%), 8% (IQR −2% to 18%), and 6% (IQR −3% to 17%) starting early, at peak, and late, respectively. Combining both OCV and POUWT led to 38% (IQR 11% to 62%) shorter epidemics when starting early, 24% (IQR 12% to 33%) shorter epidemics when starting at peak time, and 15% (IQR 5% to 25%) shorter epidemics when starting late. The number of people needed to target per case averted with a district-targeted campaign starting early was 44 (IQR 27 to 78) times higher than for CATIs within a radius of 100 m with OCV, 18 (IQR 8 to 40) times higher than for CATIs with POUWT, and 50 (IQR 33 to 93) times higher than for CATIs with OCV and POUWT combined (Fig 8).

### Operational considerations

The maximum number of rings needed to target per day varied with intervention type and timing of the start of interventions and was lower for interventions that truncated epidemics faster (Table 3). For CATIs with OCV alone, a maximum of 13 (IQR 9 to 19) rings per day needed to be targeted when starting early in a radius of 100 m. Adding POUWT to this reduced the maximum number of rings to 11 (IQR 8 to 16). The average number of rings needed to target each day was considerably lower, at 4.1 (IQR 2.7 to 6.0) rings per day for OCV and 3.7 (IQR 2.5 to 5.3) rings per day for OCV and POUWT, starting early with a radius of 100 m around reported cases.

**Table 3:** Number of rings needed to target per day when implementing different case-area targeted interventions in a 100-m radius starting early, around the peak, and late in the epidemic (median (IQR)).

Intervention	Maximum number of rings needed to target per day			Average number of rings needed to target per day		
	Early	Peak	Late	Early	Peak	Late
Antibiotics	37 (20, 60)	43 (23, 71)	27 (14, 44)	10.1 (5.4, 16.7)	12.1 (6.6, 18.9)	5.0 (3.0, 7.4)
OCV	13 (9, 19)	40 (22, 63)	28 (15, 45)	4.1 (2.7, 6.0)	11.7 (6.6, 18.7)	9.1 (4.9, 14.3)
POUWT	22 (13, 38)	41 (24, 67)	28 (15, 44)	6.7 (3.8, 11.9)	13.3 (7.4, 21.9)	6.2 (3.8, 9.1)
Antibiotics and OCV	9 (6, 13)	34 (18, 55)	27 (14, 44)	2.8 (2.0, 4.1)	7.9 (4.5, 12.1)	6.9 (4.0, 10.6)
Antibiotics and POUWT	17 (10, 29)	36 (19, 59)	27 (14, 43)	4.7 (2.7, 8.7)	9.7 (5.2, 16.7)	4.8 (2.9, 7.1)
OCV and POUWT	11 (8, 16)	37 (20, 59)	28 (15, 44)	3.7 (2.5, 5.3)	11.0 (6.2, 16.9)	9.1 (5.0, 14.5)
Antibiotics, OCV, and POUWT	9 (6, 13)	34 (18, 56)	27 (14, 44)	2.7 (1.8, 3.9)	7.7 (4.6, 12.1)	7.2 (3.9, 11.1)

OCV, oral cholera vaccine; POUWT, point-of-use water treatment.

### Sensitivity analyses

We performed several sensitivity analyses to explore the impact of key model assumptions on our results. These analyses related to assumptions about the natural history of cholera, transmission pathways, and mechanisms by which interventions protect, and assumptions related to more practical elements. Detailed descriptions of the implementation and results of the sensitivity analyses are reported in S1 Text.

In our primary analyses, we assumed that asymptomatically infected individuals are not infectious, based on the evidence that they produce far less stool and that the stool they do produce contains *V*. *cholerae* for less time and of orders of magnitude lower concentration than symptomatic cases [45,46,55]. To explore if the shedding of *V*. *cholerae* by asymptomatic individuals may have an influence on intervention outcomes, we recalibrated the model and ran additional simulations assuming that each asymptomatic person was 10% as infectious as a symptomatic patient for a single day after infection. The results suggest that allowing asymptomatic individuals to be infectious reduced the overall impact of CATIs, both in terms of cases averted and epidemic time reduced. However, the overall rank order of different interventions did not change (S23 Fig). For example, using CATIs with OCV in a 100-m radius around the peak of an epidemic in a model with infectious asymptomatic individuals led to a 21% (IQR 15% to 31%) reduction in cases averted compared to a 43% (IQR 35% to 49%) reduction in our primary analysis.

In our main analysis, all symptomatic cases were assumed to be detected and followed with a CATI deployment. However, this is unlikely to be the case in reality for a variety of reasons related to inadequacies in surveillance, care-seeking behavior, and logistical constraints. To explore how imperfect CATI response could impact our main findings, we reran simulations targeting OCV at a 100-m radius around a fraction (5%–100%) of symptomatic cases. Results suggest that CATIs responding to as few as 40% of symptomatic cases led to similar impact in terms of cases averted and epidemic days truncated (S20 and S21 Figs). Targeting 100-m rings around 40% of all symptomatic cases with OCV around the peak of an epidemic led to 38% (IQR 30% to 44%) fewer cases than simulations without interventions, and the epidemic duration was reduced by 31% (IQR 22% to 41%). As expected, when targeting CATIs within 100 m around very few symptomatic cases (5%), we saw a significant decrease in the intervention effect.

In our primary analyses, we assumed that POUWT interventions reduce the likelihood of infection by reducing exposure to *V*. *cholerae*. However, it may be that POUWT reduces the likelihood of becoming symptomatic given the clear dose–response relationship seen in a human challenge model of *V*. *cholerae* O1 [46,56,57]. To understand how this mechanistic assumption impacts our primary results, we reran simulations assuming POUWT reduced the risk of symptomatic infection by 74% (95% CI 65% to 85%) instead of reducing exposure. With this alternative POUWT mechanism, CATIs implemented within 100 m around case households led to a larger impact (cases and epidemic days averted) than in our primary analysis (S22 Fig). For example, when intervening around the peak of an epidemic in a radius of 100 m, the number of cases was reduced by 39% (IQR 31% to 44%) and the epidemic duration was reduced by 25% (IQR 16% to 34%), compared with 20% (IQR 14% to 27%) and 15% (IQR 4% to 24%), respectively, in the primary analyses.

Finally, the structure of our model makes long-distance transmission events, which cause the rapid spread of the disease from one neighborhood to another by the means of travelers and commuters [30,42], unlikely to occur. We thus performed sensitivity analyses investigating the influence of adding long-distance transmission events to the model and found that the results were qualitatively similar to those of the primary analyses (S1 Text; S16–S19 Figs).

## Discussion

Using a spatially explicit transmission model fit to data from a large cholera outbreak in Chad, we found that CATIs using OCV, antibiotics, and/or POUWT have the potential to efficiently and effectively mitigate the impact of cholera epidemics in similar urban areas. Of the 3 intervention types explored, OCV most effectively stopped epidemics (e.g., simulated epidemic durations were cut by a third when CATIs started around the epidemic peak and by a fourth when started late in the epidemic), whereas antibiotics had a more pronounced short-term impact. Combinations of the 3 types of interventions can be used to further reduce cases and deaths, although the combinations, as modeled, did not lead to synergistic effects. Our findings suggest that CATIs, which require targeting tens to hundreds of persons per case averted, are far more resource-efficient than mass intervention campaigns, which typically require hundreds to tens of thousands of people to be targeted per case averted. The optimal ring size around a case depends on the type of intervention. For antibiotics, which offer only short-term protection, the optimal CATI radius is around 30 m to 45 m, whereas for OCV and POUWT, which offer longer-lasting protection, the intervention impact increases until CATI radii reach 70 m to 100 m.

Visiting case households is not new to public health [58–60] nor to cholera control [7]. In a number of countries, it is standard practice to visit the households of cholera cases to provide health hygiene education, soap, and sometimes water and or latrine disinfectants and antibiotics [7,18]. While our findings suggest that CATIs work well even when only a fraction of cases are targeted, rapid detection and confirmation of cases is key to maximizing impact. The use of cholera rapid tests may provide one avenue for improving the precision and timeliness of CATIs given that traditional diagnostics (i.e., culture) require days to complete for some patients [61]. CATIs are logistically complex to implement and require well-trained, highly mobile teams that rapidly visit case households, delimit target areas, transport necessary supplies, and deliver interventions.

Knowing that an average of 295 (range 55 to 456) people live in each 100-m ring in N’Djamena (and similar African cities with cholera like Conakry, Monrovia, Lubumbashi, or Nairobi [62]; S12 Fig), and that several rings may overlap, the number of rings that are to be targeted each day during different variants of CATIs can provide understanding of the number of teams required to implement interventions. Other challenges to CATI implementation include finding cases’ households and negotiating with local leaders to efficiently deliver the interventions.

While our study suggests that CATIs can be effective and efficient, it remains unclear when this approach should be used, particularly in contrast to mass campaigns, the current standard for outbreak response. As shown, early CATIs can have profound impacts on the trajectory of an epidemic. If resources such as OCV are limited, as they are at the time of writing this paper, CATIs may be the most appropriate strategy to target those at highest risk. When only a few cases are detected, especially when they are spatially dispersed, ministries of health may want to initiate CATIs to efficiently quell the epidemic with supplies already in the country, while making contingency plans for mass interventions if the epidemic continues to grow. Finally, these strategies may be used late in epidemics or epidemic seasons, possibly after a mass campaign, to quickly stop the often protracted tails of epidemics [18,63], which can ultimately save scarce health system resources used during time periods when an epidemic is officially ongoing. CATIs with POUWT interventions, focused on larger geographical units (villages) than simulated in this paper, are currently being deployed to fight the cholera epidemic in Haiti through rapid response teams, although their effectiveness in reducing cases and deaths remains unknown [21].

Prophylactic antibiotic use is not part of most guidelines on cholera prevention and control; however, a number of countries, like Kenya, use antibiotics for household contacts as part of their standard outbreak response. Our results suggest that antibiotics could play a role in CATIs, although the potential impact appears lower than for other interventions in the main analyses. It is unclear how a short (or single-dose) course of antibiotics used in CATIs would affect short- and long-term antimicrobial resistance profiles in the community. Any decision to use CATIs with antibiotics should be accompanied by increased antimicrobial resistance monitoring in the community [64].

While our study used a rigorous approach to calibrate the models and capture uncertainty in both the epidemic processes and the intervention effects, it comes with a number of limitations. First, cases used in these analyses were suspected cholera cases, only some of which were confirmed [29]. If confirmed cases represent a simple random proportion of suspected cases (in terms of space and time), our characterization of space–time clustering should not be affected [32]. It is possible that cases living around other cases are more likely to present for care, which could lead to an artificial increase in our estimates of the space–time clustering of cases, meaning that our estimated impact of CATIs could be overly optimistic. However, given that estimates of space–time clustering have been relatively similar across settings with different surveillance systems and health-seeking behavior [22,25,29], we do not believe that this is likely to cause major biases in this study.

We made a number of assumptions related to cholera transmission processes. In our main analyses we assumed that asymptomatic individuals were not infectious based on evidence that asymptomatic individuals have an orders-of-magnitude lower concentration of bacteria in their stool, shed virus for less time, and excrete less stool [45]. In sensitivity analyses, we found that the results were qualitatively similar when this assumption was relaxed although the overall impact of CATIs was diminished. We did not account for a potential hyperinfectious state, where freshly shed *V*. *cholerae* may be orders of magnitude more infectious [65,66]. However, if the hyperinfectious state plays an important role in transmission, we expect that it would be in part reflected by clustering at short distances, which should be accounted for in our model through the τ fitting of the function.

The quantification of the effects and mechanisms of each intervention were based on limited data. The purpose of this study was to explore the impact of CATIs over the course of a single outbreak, disregarding the long-term effects of interventions, which may influence future epidemics. Given that the effectiveness of OCV and POUWT interventions likely wane differently over time, it is possible that there are different optimal mixes of interventions depending on the timescale of interest. The protective effects assumed for prophylactic antibiotic use were based on meta-analyses, clinical studies, and modeling studies (S1 Text) representative of the current state of evidence, some of which only included data on post-exposure antibiotic use. However, as pointed out by a recent meta-analysis [8], the studies supporting the effectiveness of prophylactic antibiotic use have a high risk of bias. We incorporated modest variability in the protective effects given the limited and sometimes weak data and the diversity of different drugs studied in the literature [51]. We assumed that antibiotic effects lasted only 2 days based on a review of the literature and consultations with experts; however, if the duration was longer, we expect that the impact of antibiotics would be larger than our results suggest. For OCV, we assumed that a single-dose regimen had an efficacy of 67% for the duration of an outbreak although there exists only 1 clinical trial estimating the single-dose efficacy—in Bangladesh over a 6-month period, where cholera is highly endemic [50]—and 1 observational study, in South Sudan, measuring short-term protection in the first 2 months after vaccination [15].

While there are a variety of WaSH interventions used to fight cholera [6], we chose to model POUWT as it is often a key WaSH intervention in outbreak settings. POUWT interventions come in different shapes and sizes and can only be effective if people appropriately comply with the intervention, which poses challenges to incorporating them into a generalizable model. We based the POUWT effect estimate on a meta-analysis of diarrhea reductions from POUWT interventions conducted in a variety of settings, including some that do not regularly report cholera cases (e.g., Bolivia) [6,67,68]. By using these estimates, we implicitly assumed that the reductions in cholera from POUWT interventions would be similar to reductions in diarrhea, which may not be the case if the diarrhea in the studies in the meta-analysis was caused by etiologic agents with different transmission pathways than cholera. For simplicity, in our main analysis we assumed that the entire mechanism of POUWT protection was to reduce the exposure probability to *V*. *cholerae*, although POUWT may both reduce exposure to water with *V*. *cholerae* and reduce the likelihood of becoming symptomatic by reducing the concentration of bacteria in any contaminated water [56]. With CATIs, it is likely that each household will have a single opportunity to receive behavior change messaging and training on using the POUWT intervention, which could lead to lower compliance, and thus lower effectiveness against cholera, than what we modeled, especially as the time since the household visit increases [68]. However, during outbreaks, mass behavior change campaigns (e.g., through radio) are common and may help sustain any changes in behavior catalyzed in the CATI household visit.

Although the modeled relative risk, τ, matches the observed data well, we did not fit τ at very short distances (i.e., between 0 m and 15 m from a case) as our model does not include household structure and is based on a grid of 30 m by 30 m cells. Small-scale spatial structure has been shown to have significant impact on transmission for other diseases [69], and neglecting it could lead to underestimation of the effect of CATIs in our model. This limitation means that we could not adequately capture the effects of targeting household contacts with interventions, which may be especially important for antibiotics [8,70].

Our results are based on a large number of epidemic trajectories of a model fit to a single outbreak; however, the relative impact of CATIs is largely shaped by the spatiotemporal clustering of cholera cases, which has been shown to be similar in both epidemic and endemic settings around the globe [22–29,61]. This commonality between the clustering of cholera cases provides reassurance that our findings may not only be representative of the potential impacts in this single outbreak but likely reflect (qualitatively) the impacts in other similar settings. Thus, we advocate that CATIs can be a promising approach to control cholera epidemics in urban areas. While the optimal target radius may vary between settings, due to population density and logistical constraints, antibiotic interventions will likely continue to have a smaller optimal radius than OCV and POUWT, because of the duration of protection and likely delays in responding to each ring, which are unlikely to vary significantly across settings.

Our results suggest that CATIs may be an effective and efficient approach to reducing morbidity and mortality and saving public health resources in cholera epidemics. While more work is needed to understand how and when to best use this approach in outbreaks across different settings, taking into account both human resource capacity and supply availability, our study provides data-based support to public health programs currently using CATIs to fight cholera.

## Supporting information

S1 Fig Map of cholera cases. Map of the city of N’Djamena and the locations of cases with available GPS coordinates by time of reporting. Red lines show the limits of the 10 districts (arrondissements) of N’Djamena. (Background map: Tiles by CartoDB, under CC BY 3.0. Data by OpenStreetMap, under ODbL. Administrative subdivision: OpenStreetMap, under ODbL.)
(PDF)

S2 Fig Proxy for population density. Map of the city of N’Djamena with built-up density (in percent) [37,38] of each 30 m by 30 m grid cell. Values equal to 0 or located outside the city boundary are transparent. (Background map: Tiles by CartoDB, under CC BY 3.0. Data by OpenStreetMap, under ODbL.)
(PDF)

S3 Fig Posterior parameters. Marginal posterior parameter distributions computed from 1,000 samples. Blue shaded ranges along the axes show the intervals within which parameters were allowed to vary during calibration.
(PDF)

S4 Fig Simulated evolution of the epidemics without intervention and with case-area targeted allocation of combinations of the 3 main intervention types within a 100-m radius starting around the epidemic peak. The lower panels in each pair of panels show the number of people targeted during each timestep and the number of people protected by the interventions. Solid lines show the median over all simulations, shaded areas the 2.5th and 97.5th percentiles. The red bar at the top of each panel marks the period during which interventions are applied.
(PDF)

S5 Fig Outcome of combinations of the 3 main intervention types with case-area targeted allocation in a 100-m radius. Boxplots of the number of averted cases, the number of targeted persons, and the number of targeted clusters predicted by the model for combinations of the 3 main intervention types with case-area targeted allocation in a 100-m radius starting at 3 different times. Abx stands for antibiotics. Abx* stands for administering antibiotics only within a range of 15 m, while OCV is administered within the whole cluster. Whiskers mark the 2.5th and 97.5th percentiles.
(PDF)

S6 Fig Outcome of the 3 main intervention types in mass intervention campaigns. Mass intervention campaigns targeting 70% of the entire city population (left column) and 70% of the people living in the 3 districts with the highest attack rate at the onset of the interventions (right column). Boxplots show the number of averted cases (top row) and the number of targeted persons (bottom row) predicted by the model. Whiskers mark the 2.5th and 97.5th percentiles. Boxplots of the number of targeted persons in a mass intervention campaign of the entire city collapse because the number of people to target was fixed.
(PDF)

S7 Fig Outcome of the 3 main intervention types in small city-wide mass campaigns. Boxplots of the number of averted cases and the number of targeted persons predicted by the model. The number of targeted persons has been fixed to the same values for case-area targeted allocation with a 100-m radius (Fig 5). Whiskers mark the 2.5th and 97.5th percentiles.
(PDF)

S8 Fig Intervention outcomes of case-area targeted allocation of OCV within a varying radius combined with the allocation of antibiotics within a radius of 15 m. The number of averted cases, the number of targeted persons, and the number of targeted clusters predicted by the model with case-area targeted allocation and variable radius, starting at 3 different times. The error bars cover the range between the 25th and 75th quantile over all simulations.
(PDF)

S9 Fig Comparison between single and repeated antibiotic administration through CATIs in a 100-m radius. Boxplots of the number of averted cases, the number of targeted persons, and the number of targeted clusters predicted by the model for 2 different strategies of allocating antibiotics in CATIs within a 100-m radius starting at 3 different times. Whiskers mark the 2.5th and 97.5th percentiles. Single allocation designates the standard mode, where every person can receive 1 dose of antibiotics only once during the epidemic. Repeated allocation designates a mode where a person can get antibiotics several times, with a minimal interval of 2 weeks, if he/she lives within the intervention radius of several cases.
(PDF)

S10 Fig Comparison of relative risk τ evaluated in 15-m distance ranges for different time ranges. (PDF)

S11 Fig Daily precipitation depth in N’Djamena from April 2011 to April 2012. (PDF)

S12 Fig Number of people living within a given range in N’Djamena. Histograms of the number of people living within a circle with radius 15 m (A), 30 m (B), 45 m (C), 70 m (D), and 100 m (E) in N’Djamena obtained by sampling the population distribution at 1,000 random points. The bin width in the histograms is 10.
(PDF)

S13 Fig Circles of given radius (in meters) as implemented in the regular model grid. The side length of each square is 30 m.
(PDF)

S14 Fig Distributions of intervention parameters. Intervention parameters related to antibiotics (reduction of symptomatic fraction [A] and reduction of duration of shedding [B]), OCV (reduction of symptomatic fraction [C]), and POUWT (reduction of exposure [D]). (A) was obtained from Lewnard et al. [49] directly, whereas (B) (normal), (C) (log-normal), and (D) (log-normal) were fitted to the corresponding confidence intervals given in Table 1.
(PDF)

S15 Fig Delay between symptom onset and deployment of an intervention team. Probability distribution of the delay (in days) between the onset of symptoms of the initial case in a cluster and the deployment of an intervention team.
(PDF)

S16 Fig Calibrated model fit with additional long-distance transmission. (A) shows the distribution of daily incident cholera cases from uncontrolled epidemic simulations. The shaded areas represent the marginal interquartile range (dark blue) and the 2.5th and 97.5th percentiles (light blue) from 1,000 simulated epidemics, with the true number of daily reported cases shown as red dots. Red ticks at the top of (A) represent the 3 times when interventions start. (B) shows the interquartile range (dark blue) and 2.5th and 97.5th posterior percentiles (light blue) of the relative risk (τ statistic) of the next case being within a specific distance from a case within 5 days of his/her symptom onset. Red dots and bars (95% confidence intervals) represent the computed τ from the data.
(PDF)

S17 Fig Posterior parameters with long-distance transmission. Marginal posterior parameter distributions computed from 1,000 samples. Blue shaded ranges along the axes show the intervals within which parameters were allowed to vary during calibration.
(PDF)

S18 Fig Comparison of the simulated evolution including long-distance transmission of the epidemics with and without CATIs. Upper panels in each pair of panels show the evolution of the epidemics simulated including long-distance transmission without intervention and with case-area targeted allocation of antibiotics, OCV, and POUWT within a 100-m radius starting at the epidemic peak. Lower panels in each pair of panels show the corresponding number of people targeted during each timestep and the number of people protected by each intervention. Solid lines designate the median over all simulations, shaded areas the 2.5th and 97.5th percentiles. The red bars at the top of the panels mark the period during which interventions were applied.
(PDF)

S19 Fig Comparison of intervention outcomes with (*c* = 0.05) and without (*c* = 0) long-distance transmission. Boxplots of the number of averted cases, the number of targeted persons, and the number of targeted clusters predicted by 2 models without and with 5% long-distance transmission events and by of allocating OCV, antibiotics, and POUWT in CATIs within a 100-m radius starting at 3 different times. Whiskers mark the 2.5th and 97.5th percentiles.
(PDF)

S20 Fig Comparison of outcomes when targeting CATIs using OCV within 100 m around a fraction of symptomatic cases. Boxplots of the number of averted cases, the number of targeted persons, and the number of targeted clusters predicted when targeting CATIs using OCV within 100 m around 100%, 80%, 60%, 40%, and 5% of symptomatic cases and with district-targeted mass campaigns. Colors denote campaigns starting at 3 different times. Whiskers mark the 2.5th and 97.5th percentiles.
(PDF)

S21 Fig Comparison of the reduction of epidemic duration when targeting CATIs using OCV within 100 m around a fraction of symptomatic cases. Boxplots of the number of epidemic days reduced predicted when targeting CATIs using OCV within 100 m around 100%, 80%, 60%, 40%, and 5% of symptomatic cases and with district-targeted mass campaigns. Colors denote campaigns starting at 3 different times. Whiskers mark the 2.5th and 97.5th percentiles.
(PDF)

S22 Fig Comparison of outcomes of CATIs using different mechanisms to implement POUWT. Boxplots of the number of averted cases, the number of targeted persons, and the number of targeted clusters predicted when implementing CATIs using POUWT in a 100-m radius around reported cases through reduction of the likelihood of getting infected (mechanism of our main analysis) or through reduction of the likelihood of getting symptoms (mechanism in sensitivity analysis). Colors denote campaigns starting at 3 different times. Whiskers mark the 2.5th and 97.5th percentiles.
(PDF)

S23 Fig Comparison of outcomes of CATIs with and without shedding of *V*. *cholerae* by asymptomatically infected individuals. Boxplots of the relative number of averted cases (averted cases divided by number of cases without intervention) and the relative number of epidemic days reduced (number of epidemic days reduced divided by the number of epidemic days without intervention) with asymptomatic individuals being 10% as infectious as symptomatic individuals, compared to the results of our main analysis (no shedding by asymptomatic individuals), with different CATIs in a 100-m radius. Colors denote campaigns starting at 3 different times. Whiskers mark the 2.5th and 97.5th percentiles.
(PDF)

S1 Table Summary of all intervention scenarios considered, with number of targeted clusters and number of averted cases. The table shows all intervention scenarios considered, specifically the type of intervention, the timing, and the type of allocation, together with median values (2.5% and 97.5% percentiles in brackets) of the number of targeted clusters, the total number of targeted people, and the total number of averted cases computed using 1,000 model simulations. *Every person can get antibiotics only once during the epidemic. **Every person can get antibiotics several times with a minimum delay of 2 weeks between 2 administrations.
(PDF)

S1 Text Supplementary materials and methods. Description of the data used, the implementation of the epidemiological model, its calibration, and the implementation of intervention strategies.
(PDF)

S1 Alternative Language Abstract French translation of the abstract by Maya Allan, Étienne Gignoux, Javier Perez-Saez, and Flavio Finger. (PDF)

## Abbreviations

CATI: case-area targeted intervention
OCV: oral cholera vaccine
MSF: Médecins Sans Frontières
POUWT: point-of-use water treatment
WaSH: water, sanitation, and hygiene

## References

[1] World Health Organization. Cholera, 2016. Wkly Epidemiol Rec. 2017;92(36):521–36. 28884993

[2] Ali M, Nelson AR, Lopez AL, Sack DA. Updated global burden of cholera in endemic countries. PLoS Negl Trop Dis. 2015;9(6):e0003832 doi: 10.1371/journal.pntd.0003832 26043000 10.1371/journal.pntd.0003832 PMC4455997

[3] Luquero FJ, Rondy M, Boncy J, Munger A, Mekaoui H, Rymshaw E, et al Mortality rates during cholera epidemic, Haiti, 2010–2011. Emerg Infect Dis. 2016;22(3):410–6. doi: 10.3201/eid2203.141970 26886511 10.3201/eid2203.141970 PMC4766911

[4] Rebaudet S, Sudre B, Faucher B, Piarroux R. Cholera in coastal Africa: a systematic review of its heterogeneous environmental determinants. J Infect Dis. 2013;208(Suppl 1):S98–106. doi: 10.1093/infdis/jit202 24101653 10.1093/infdis/jit202

[5] Rebaudet S, Sudre B, Faucher B, Piarroux R. Environmental determinants of cholera outbreaks in inland Africa: a systematic review of main transmission foci and propagation routes. J Infect Dis. 2013;208(Suppl 1):S46–54. doi: 10.1093/infdis/jit195 24101645 10.1093/infdis/jit195

[6] Fewtrell L, Kaufmann RB, Kay D, Enanoria W, Haller L, Colford JM Jr. Water, sanitation, and hygiene interventions to reduce diarrhoea in less developed countries: a systematic review and meta-analysis. Lancet Infect Dis. 2005;5(1):42–52. doi: 10.1016/S1473-3099(04)01253-8 15620560 10.1016/S1473-3099(04)01253-8

[7] Guévart É, Noeske J, Sollé J, Mouangue A, Bikoti JM. Antibioprophylaxie ciblée à large échelle au cours de l’épidémie de choléra de Douala en 2004. Santé. 2007;17(2):63–8. 17962152

[8] Reveiz L, Chapman E, Ramon-Pardo P, Koehlmoos TP, Cuervo LG, Aldighieri S, et al Chemoprophylaxis in contacts of patients with cholera: systematic review and meta-analysis. PLoS ONE. 2011;6(11):e27060 doi: 10.1371/journal.pone.0027060 22102873 10.1371/journal.pone.0027060 PMC3216950

[9] Desai SN, Pezzoli L, Martin S, Costa A, Rodriguez C, Legros D, et al A second affordable oral cholera vaccine: implications for the global vaccine stockpile. Lancet Glob Health. 2016;4(4):e223–4. doi: 10.1016/S2214-109X(16)00037-1 27013303 10.1016/S2214-109X(16)00037-1

[10] Martin S, Costa A, Perea W. Stockpiling oral cholera vaccine. Bull World Health Organ. 2012;90(10):714 doi: 10.2471/BLT.12.112433 23109735 10.2471/BLT.12.112433 PMC3471062

[11] Parker LA, Rumunu J, Jamet C, Kenyi Y, Lino RL, Wamala JF, et al Adapting to the global shortage of cholera vaccines: targeted single dose cholera vaccine in response to an outbreak in South Sudan. Lancet Infect Dis. 2017;17(4):e123–7. doi: 10.1016/S1473-3099(16)30472-8 28109819 10.1016/S1473-3099(16)30472-8

[12] Bhattacharya SK, Sur D, Ali M, Kanungo S, You YA, Manna B, et al 5 year efficacy of a bivalent killed whole-cell oral cholera vaccine in Kolkata, India: a cluster-randomised, double-blind, placebo-controlled trial. Lancet Infect Dis. 2013;13(12):1050–6. doi: 10.1016/S1473-3099(13)70273-1 24140390 10.1016/S1473-3099(13)70273-1

[13] Bi Q, Ferreras E, Pezzoli L, Legros D, Ivers LC, Date K, et al Protection against cholera from killed whole-cell oral cholera vaccines: a systematic review and meta-analysis. Lancet Infect Dis. 2017;17(10):1080–8. doi: 10.1016/S1473-3099(17)30359-6 28729167 10.1016/S1473-3099(17)30359-6 PMC5639147

[14] Abubakar A, Azman AS, Rumunu J, Ciglenecki I, Helderman T, West H, et al The first use of the global oral cholera vaccine emergency stockpile: lessons from South Sudan. PLoS Med. 2015;12(11):e1001901 doi: 10.1371/journal.pmed.1001901 26576044 10.1371/journal.pmed.1001901 PMC4648513

[15] Azman AS, Parker LA, Rumunu J, Tadesse F, Grandesso F, Deng LL, et al Effectiveness of one dose of oral cholera vaccine in response to an outbreak: a case-cohort study. Lancet Glob Health. 2016;4(11):e856–63. doi: 10.1016/S2214-109X(16)30211-X 27765293 10.1016/S2214-109X(16)30211-X

[16] Ciglenecki I, Sakoba K, Luquero FJ, Heile M, Itama C, Mengel M, et al Feasibility of mass vaccination campaign with oral cholera vaccines in response to an outbreak in Guinea. PLoS Med. 2013;10(9):e1001512 doi: 10.1371/journal.pmed.1001512 24058301 10.1371/journal.pmed.1001512 PMC3769208

[17] Ivers LC, Hilaire IJ, Teng JE, Almazor CP, Jerome JG, Ternier R, et al Effectiveness of reactive oral cholera vaccination in rural Haiti: a case-control study and bias-indicator analysis. Lancet Glob Health. 2015;3(3):e162–8. doi: 10.1016/S2214-109X(14)70368-7 25701994 10.1016/S2214-109X(14)70368-7 PMC4384694

[18] Parker LA, Rumunu J, Jamet C, Kenyi Y, Lino RL, Wamala JF, et al Neighborhood-targeted and case-triggered use of a single dose of oral cholera vaccine in an urban setting: feasibility and vaccine coverage. PLoS Negl Trop Dis. 2017;11(6):e0005652 doi: 10.1371/journal.pntd.0005652 28594891 10.1371/journal.pntd.0005652 PMC5478158

[19] Piarroux R, Bompangue D, Oger PY, Boinet A, Haaser F, Vandevelde T. From research to field action: example of the fight against cholera in the Democratic Republic of Congo. Field Actions Sci Rep. 2009;2.

[20] Farmer P, Almazor CP, Bahnsen ET, Barry D, Bazile J, Bloom BR, et al Meeting cholera’s challenge to Haiti and the world: a joint statement on cholera prevention and care. PLoS Negl Trop Dis. 2011;5(5):e1145 doi: 10.1371/journal.pntd.0001145 21655350 10.1371/journal.pntd.0001145 PMC3104956

[21] Santa-Olalla P, Gayer M, Magloire R, Barrais R, Valenciano M, Aramburu C, et al Implementation of an alert and response system in Haiti during the early stage of the response to the cholera epidemic. Am J Trop Med Hyg. 2013;89(4):688–97. doi: 10.4269/ajtmh.13-0267 24106196 10.4269/ajtmh.13-0267 PMC3795099

[22] Debes AK, Ali M, Azman AS, Yunus M, Sack DA. Cholera cases cluster in time and space in Matlab, Bangladesh: implications for targeted preventive interventions. Int J Epidemiol. 2016;45(6):2134–9. doi: 10.1093/ije/dyw267 27789673 10.1093/ije/dyw267

[23] Snow J. On the mode of communication of cholera. John Churchill; 1855.

[24] Blackburn JK, Diamond U, Kracalik IT, Widmer J, Brown W, Morrissey BD, et al Household-level spatiotemporal patterns of incidence of cholera, Haiti, 2011. Emerg Infect Dis. 2014;20(9):1516–9. doi: 10.3201/eid2009.131882 25148590 10.3201/eid2009.131882 PMC4178390

[25] Ali M, Debes AK, Luquero FJ, Kim DR, Park JY, Digilio L, et al Potential for controlling cholera using a ring vaccination strategy: re-analysis of data from a cluster-randomized clinical trial. PLoS Med. 2016;13(9):e1002120 doi: 10.1371/journal.pmed.1002120 27622507 10.1371/journal.pmed.1002120 PMC5021260

[26] Luquero FJ, Banga CN, Remartnez D, Palma PP, Baron E, Grais RF. Cholera epidemic in Guinea-Bissau (2008): the importance of “place”. PLoS ONE. 2011;6(5):e19005 doi: 10.1371/journal.pone.0019005 21572530 10.1371/journal.pone.0019005 PMC3087718

[27] You YA, Ali M, Kanungo S, Sah B, Manna B, Puri M, et al risk map of cholera infection for vaccine deployment: the eastern Kolkata case. PLoS ONE. 2013;8(8):e71173 doi: 10.1371/journal.pone.0071173 23936491 10.1371/journal.pone.0071173 PMC3732263

[28] Carrel M, Emch M, Streatfield PK, Yunus M. Spatio-temporal clustering of cholera: the impact of flood control in Matlab, Bangladesh, 1983–2003. Health Place. 2009;15(3):771–82. doi: 10.1016/j.healthplace.2008.12.008 19217821 10.1016/j.healthplace.2008.12.008 PMC2790410

[29] Azman A, Luquero FJ, Salje H, Naibei N, Adalbert N, Ali M, et al Micro-hotspots of risk in urban cholera epidemics. bioRxiv. 2018 1 18 doi: 10.1101/248476 10.1093/infdis/jiy283 PMC6107744 29757428

[30] Azman AS, Lessler J. Reactive vaccination in the presence of disease hotspots. Proc Biol Sci. 2015;282(1798):20141341 doi: 10.1098/rspb.2014.1341 25392464 10.1098/rspb.2014.1341 PMC4262159

[31] Azman AS, Rumunu J, Abubakar A, West H, Ciglenecki I, Helderman T, et al Population-level effect of cholera vaccine on displaced populations, South Sudan, 2014. Emerg Infect Dis. 2016;22(6):1067–70. doi: 10.3201/eid2206.151592 27192187 10.3201/eid2206.151592 PMC4880069

[32] Lessler J, Salje H, Grabowski MK, Cummings DAT. Measuring spatial dependence for infectious disease epidemiology. PLoS ONE. 2016;11(5):e0155249 doi: 10.1371/journal.pone.0155249 27196422 10.1371/journal.pone.0155249 PMC4873007

[33] Salje H, Lessler J, Endy TP, Curriero FC, Gibbons RV, Nisalak A, et al Revealing the microscale spatial signature of dengue transmission and immunity in an urban population. Proc Natl Acad Sci U S A. 2012;109(24):9535–8. doi: 10.1073/pnas.1120621109 22645364 10.1073/pnas.1120621109 PMC3386131

[34] Grabowski MK, Lessler J, Redd AD, Kagaayi J, Laeyendecker O, Ndyanabo A, et al The role of viral introductions in sustaining community-based HIV epidemics in rural Uganda: evidence from spatial clustering, phylogenetics, and egocentric transmission models. PLoS Med. 2014;11(3):e1001610 doi: 10.1371/journal.pmed.1001610 24595023 10.1371/journal.pmed.1001610 PMC3942316

[35] Salje H, Cauchemez S, Alera MT, Rodriguez-Barraquer I, Thaisomboonsuk B, Srikiatkhachorn A, et al Reconstruction of 60 years of chikungunya epidemiology in the Philippines demonstrates episodic and focal transmission. J Infect Dis. 2016;213(4):604–10. doi: 10.1093/infdis/jiv470 26410592 10.1093/infdis/jiv470 PMC4721913

[36] Salje H, Lessler J, Berry IM, Melendrez MC, Endy T, Kalayanarooj S, et al Dengue diversity across spatial and temporal scales: local structure and the effect of host population size. Science. 2017;355(6331):1302–6. doi: 10.1126/science.aaj9384 28336667 10.1126/science.aaj9384 PMC5777672

[37] Esch T, Schenk A, Ullmann T, Thiel M, Roth A, Dech S. Characterization of land cover types in TerraSAR-X images by combined analysis of speckle statistics and intensity information. IEEE Trans Geosci Remote Sens. 2011;49(6):1911–25. doi: 10.1109/TGRS.2010.2091644

[38] Esch T, Heldens W, Hirner A, Keil M, Marconcini M, Roth A, et al Breaking new ground in mapping human settlements from space—the Global Urban Footprint. ISPRS J Photogramm Remote Sens. 2017;134:30–42. doi: 10.1016/j.isprsjprs.2017.10.012

[39] Rinaldo A, Bertuzzo E, Mari L, Righetto L, Blokesch M, Gatto M, et al Reassessment of the 2010–2011 Haiti cholera outbreak and rainfall-driven multiseason projections. Proc Natl Acad Sci U S A. 2012;109(17):6602–7. doi: 10.1073/pnas.1203333109 22505737 10.1073/pnas.1203333109 PMC3340092

[40] de Magny GC, Thiaw W, Kumar V, Manga NM, Diop BM, Gueye L, et al Cholera outbreak in Senegal in 2005: was climate a factor? PLoS ONE. 2012;7(8):e44577 doi: 10.1371/journal.pone.0044577 22952995 10.1371/journal.pone.0044577 PMC3432123

[41] Gaudart J, Rebaudet S, Barrais R, Boncy J, Faucher B, Piarroux M, et al Spatio-temporal dynamics of cholera during the first year of the epidemic in Haiti. PLoS Negl Trop Dis. 2013;7(4):e2145 doi: 10.1371/journal.pntd.0002145 23593516 10.1371/journal.pntd.0002145 PMC3617102

[42] Finger F, Genolet T, Mari L, de Magny GC, Manga NM, Rinaldo A, et al Mobile phone data highlights the role of mass gatherings in the spreading of cholera outbreaks. Proc Natl Acad Sci U S A. 2016;113(23):6421–6. doi: 10.1073/pnas.1522305113 27217564 10.1073/pnas.1522305113 PMC4988598

[43] Ngwa MC, Liang S, Kracalik IT, Morris L, Blackburn JK, Mbam LM, et al Cholera in Cameroon, 2000–2012: spatial and temporal analysis at the operational (health district) and sub climate levels. PLoS Negl Trop Dis. 2016;10(11):e0005105 doi: 10.1371/journal.pntd.0005105 27855171 10.1371/journal.pntd.0005105 PMC5113893

[44] Azman AS, Rudolph KE, Cummings DAT, Lessler J. The incubation period of cholera: a systematic review. J Infect. 2013;66(5):432–8. doi: 10.1016/j.jinf.2012.11.013 23201968 10.1016/j.jinf.2012.11.013 PMC3677557

[45] Nelson EJ, Harris JB, Glenn Morris J, Calderwood SB, Camilli A. Cholera transmission: the host, pathogen and bacteriophage dynamic. Nat Rev Microbiol. 2009;7(10):693–702. doi: 10.1038/nrmicro2204 19756008 10.1038/nrmicro2204 PMC3842031

[46] Fung ICH. Cholera transmission dynamic models for public health practitioners. Emerging Themes in Epidemiology. 2014;11:1 doi: 10.1186/1742-7622-11-1 24520853 10.1186/1742-7622-11-1 PMC3926264

[47] Kaper JB, Morris JG, Levine MM. Cholera. Clin Microbiol Rev. 1995;8(1):48–86. 7704895 10.1128/cmr.8.1.48 PMC172849

[48] Akeret J, Refregier A, Amara A, Seehars S, Hasner C. Approximate Bayesian computation for forward modeling in cosmology. J Cosmol Astropart Phys. 2015;2015(08):043 doi: 10.1088/1475-7516/2015/08/043

[49] Lewnard JA, Antillón M, Gonsalves G, Miller AM, Ko AI, Pitzer VE. Strategies to prevent cholera introduction during international personnel deployments: a computational modeling analysis based on the 2010 Haiti outbreak. PLoS Med. 2016;13(1):e1001947 doi: 10.1371/journal.pmed.1001947 26812236 10.1371/journal.pmed.1001947 PMC4727895

[50] Qadri F, Wierzba TF, Ali M, Chowdhury F, Khan AI, Saha A, et al Efficacy of a single-dose, inactivated oral cholera vaccine in Bangladesh. N Engl J Med. 2016;374(18):1723–32. doi: 10.1056/NEJMoa1510330 27144848 10.1056/NEJMoa1510330

[51] Leibovici-Weissman Y, Neuberger A, Bitterman R, Sinclair D, Salam MA, Paul M. Antimicrobial drugs for treating cholera. Cochrane Database Syst Rev. 2014;6:CD008625. 10.1002/14651858.CD008625.pub2 PMC4468928 24944120

[52] Taylor DL, Kahawita TM, Cairncross S, Ensink JHJ. The impact of water, sanitation and hygiene interventions to control cholera: a systematic review. PLoS ONE. 2015;10(8):e0135676 doi: 10.1371/journal.pone.0135676 26284367 10.1371/journal.pone.0135676 PMC4540465

[53] Khan WA, Saha D, Rahman A, Salam MA, Bogaerts J, Bennish ML. Comparison of single-dose azithromycin and 12-dose, 3-day erythromycin for childhood cholera: a randomised, double-blind trial. Lancet. 2002;360(9347):1722–7. doi: 10.1016/S0140-6736(02)11680-1 12480424 10.1016/S0140-6736(02)11680-1

[54] Echevarria J, Seas C, Carrillo C, Mostorino R, Ruiz R, Gotuzzo E. Efficacy and tolerability of ciprofloxacin prophylaxis in adult household contacts of patients with cholera. Clin Infect Dis. 1995;20(6):1480–4. doi: 10.1093/clinids/20.6.1480 7548495 10.1093/clinids/20.6.1480

[55] Cash RA, Music SI, Libonati JP, Snyder MJ, Wenzel RP, Hornick RB. Response of man to infection with Vibrio cholerae. I. Clinical, serologic, and bacteriologic responses to a known inoculum. J Infect Dis. 1974;129(1):45–52. doi: 10.1093/infdis/129.1.45 4809112 10.1093/infdis/129.1.45

[56] Hornick RB, Music SI, Wenzel R, Cash R, Libonati JP, Snyder MJ, et al The Broad Street pump revisited: response of volunteers to ingested cholera vibrios. Bull N Y Acad Med. 1971;47(10):1181–91. 5286453 PMC1749960

[57] Grad YH, Miller JC, Lipsitch M. Cholera modeling: challenges to quantitative analysis and predicting the impact of interventions. Epidemiology. 2012;23(4):523–30. doi: 10.1097/EDE.0b013e3182572581 22659546 10.1097/EDE.0b013e3182572581 PMC3380087

[58] Hitchings MDT, Grais RF, Lipsitch M. Using simulation to aid trial design: ring-vaccination trials. PLoS Negl Trop Dis. 2017;11(3):e0005470 doi: 10.1371/journal.pntd.0005470 28328984 10.1371/journal.pntd.0005470 PMC5378415

[59] Kucharski AJ, Eggo RM, Watson CH, Camacho A, Funk S, Edmunds WJ. Effectiveness of ring vaccination as control strategy for Ebola virus disease. Emerg Infect Dis. 2016;22(1):105–8. doi: 10.3201/eid2201.151410 26691346 10.3201/eid2201.151410 PMC4696719

[60] Merler S, Ajelli M, Fumanelli L, Parlamento S, y Piontti AP, Dean NE, et al Containing Ebola at the source with ring vaccination. PLoS Negl Trop Dis. 2016;10(11):e0005093 doi: 10.1371/journal.pntd.0005093 27806049 10.1371/journal.pntd.0005093 PMC5091901

[61] Debes AK, Ateudjieu J, Guenou E, Lopez AL, Bugayong MP, Retiban PJ, et al Evaluation in Cameroon of a novel, simplified methodology to assist molecular microbiological analysis of V. cholerae in resource-limited settings. PLoS Negl Trop Dis. 2016;10(1):e0004307 doi: 10.1371/journal.pntd.0004307 26735969 10.1371/journal.pntd.0004307 PMC4703203

[62] Cox W. Demographia world urban areas. 13th annual edition. Belleville (IL): Demographia; 2017 [cited 2018 Jan 22]. Available from: http://www.demographia.com/db-worldua.pdf.

[63] Rebaudet S, Gazin P, Barrais R, Moore S, Rossignol E, Barthelemy N, et al The dry season in Haiti: a window of opportunity to eliminate cholera. PLoS Curr. 2013 6 10 doi: 10.1371/currents.outbreaks.2193a0ec4401d9526203af12e5024ddc 23873011 10.1371/currents.outbreaks.2193a0ec4401d9526203af12e5024ddc PMC3712488

[64] Wernli D, Jørgensen PS, Harbarth S, Carroll SP, Laxminarayan R, Levrat N, et al Antimicrobial resistance: the complex challenge of measurement to inform policy and the public. PLoS Med. 2017;14(8):e1002378 doi: 10.1371/journal.pmed.1002378 28817562 10.1371/journal.pmed.1002378 PMC5560527

[65] Hartley DM, Morris JG Jr, Smith DL. Hyperinfectivity: a critical element in the ability of V. cholerae to cause epidemics? PLoS Med. 2006;3(1):e7 doi: 10.1371/journal.pmed.0030007 16318414 10.1371/journal.pmed.0030007 PMC1298942

[66] Faruque SM, Biswas K, Udden SMN, Ahmad QS, Sack DA, Nair GB, et al Transmissibility of cholera: in vivo-formed biofilms and their relationship to infectivity and persistence in the environment. Proc Natl Acad Sci U S A. 2006;103(16):6350–5. doi: 10.1073/pnas.0601277103 16601099 10.1073/pnas.0601277103 PMC1458881

[67] Fewtrell L, Colford JM Jr. Water, sanitation and hygiene: interventions and diarrhoea—a systematic review and meta-analysis Washington (DC): World Bank; 2004. 10.1016/S1473-3099(04)01253-8 15620560

[68] Clasen T, Schmidt WP, Rabie T, Roberts I, Cairncross S. Interventions to improve water quality for preventing diarrhoea: systematic review and meta-analysis. BMJ. 2007;334(7597):782 doi: 10.1136/bmj.39118.489931.BE 17353208 10.1136/bmj.39118.489931.BE PMC1851994

[69] Salje H, Lessler J, Paul KK, Azman AS, Rahman MW, Rahman M, et al How social structures, space, and behaviors shape the spread of infectious diseases using chikungunya as a case study. Proc Natl Acad Sci U S A. 2016;113(47):13420–5. doi: 10.1073/pnas.1611391113 27821727 10.1073/pnas.1611391113 PMC5127331

[70] Weil AA, Begum Y, Chowdhury F, Khan AI, Leung DT, LaRocque RC, et al Bacterial shedding in household contacts of cholera patients in Dhaka, Bangladesh. Am J Trop Med Hyg. 2014;91(4):738–42. doi: 10.4269/ajtmh.14-0095 25114012 10.4269/ajtmh.14-0095 PMC4183396

