# Supplementary figures and images for: The potential impact of case-area targeted interventions in response to cholera outbreaks: A modeling study
Source: PLoS Med. 2018 Feb 27;15(2):e1002509. doi: 10.1371/journal.pmed.1002509 (PMC5828347; doi:10.1371/journal.pmed.1002509)

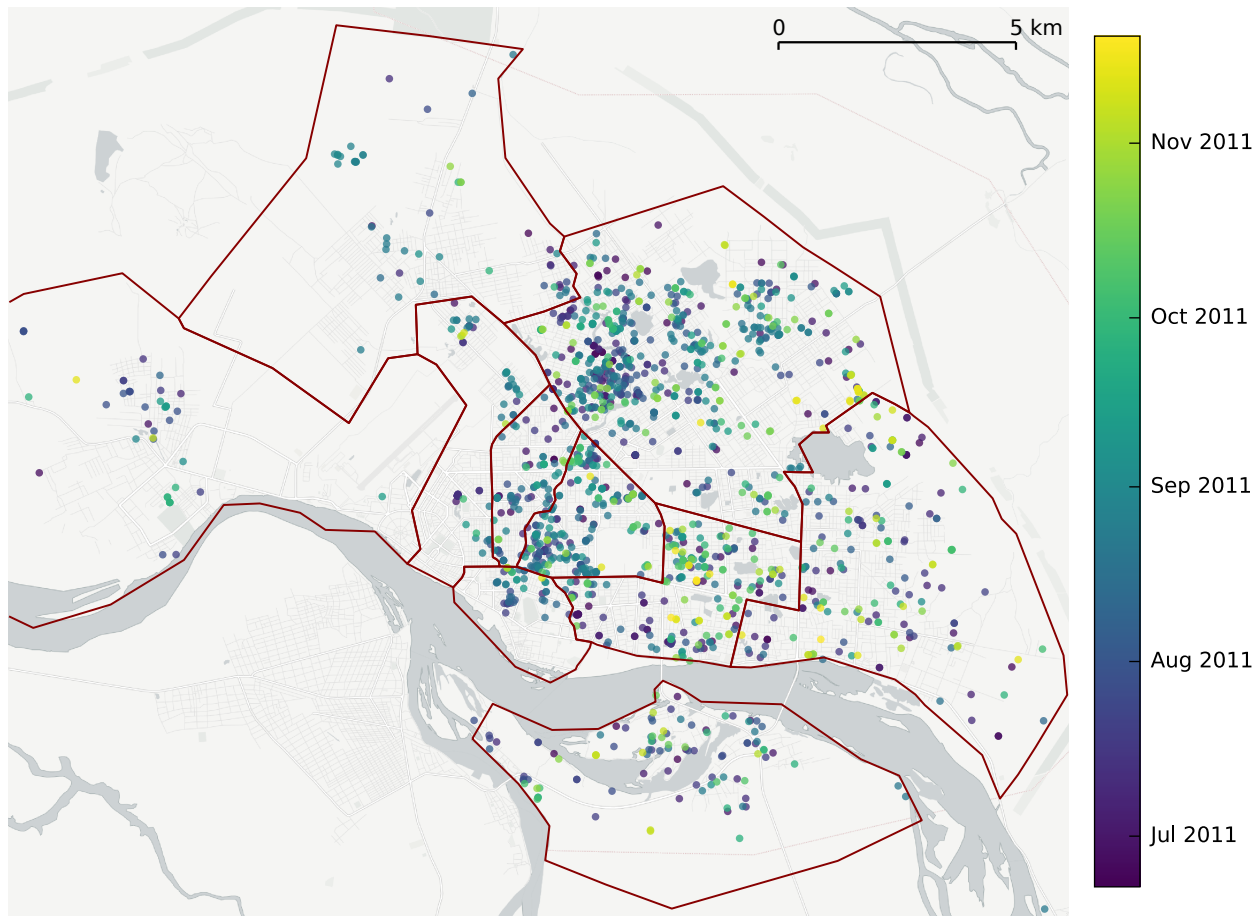

Supplement: S1 Fig — Map of the city of N’Djamena and the locations of cases with available GPS coordinates by time of reporting. Red lines show the limits of the 10 districts (arrondissements) of N’Djamena. (Background map: Tiles by CartoDB, under CC BY 3.0. Data by OpenStreetMap, under ODbL. Administrative subdivision: OpenStreetMap, under ODbL.) (PDF) [file pmed.1002509.s001.pdf]

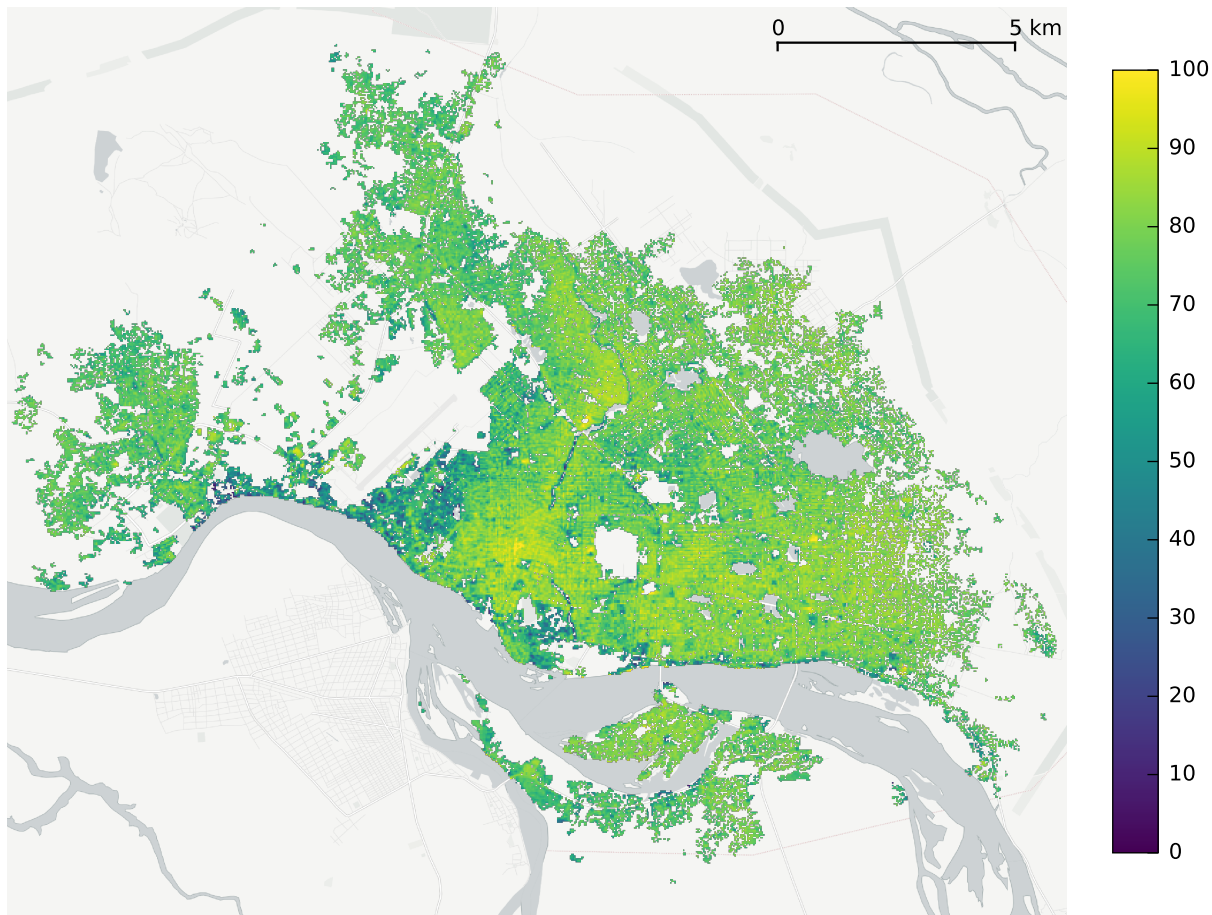

Supplement: S2 Fig — Map of the city of N’Djamena with built-up density (in percent) [37,38] of each 30 m by 30 m grid cell. Values equal to 0 or located outside the city boundary are transparent. (Background map: Tiles by CartoDB, under CC BY 3.0. Data by OpenStreetMap, under ODbL.) (PDF) [file pmed.1002509.s002.pdf]

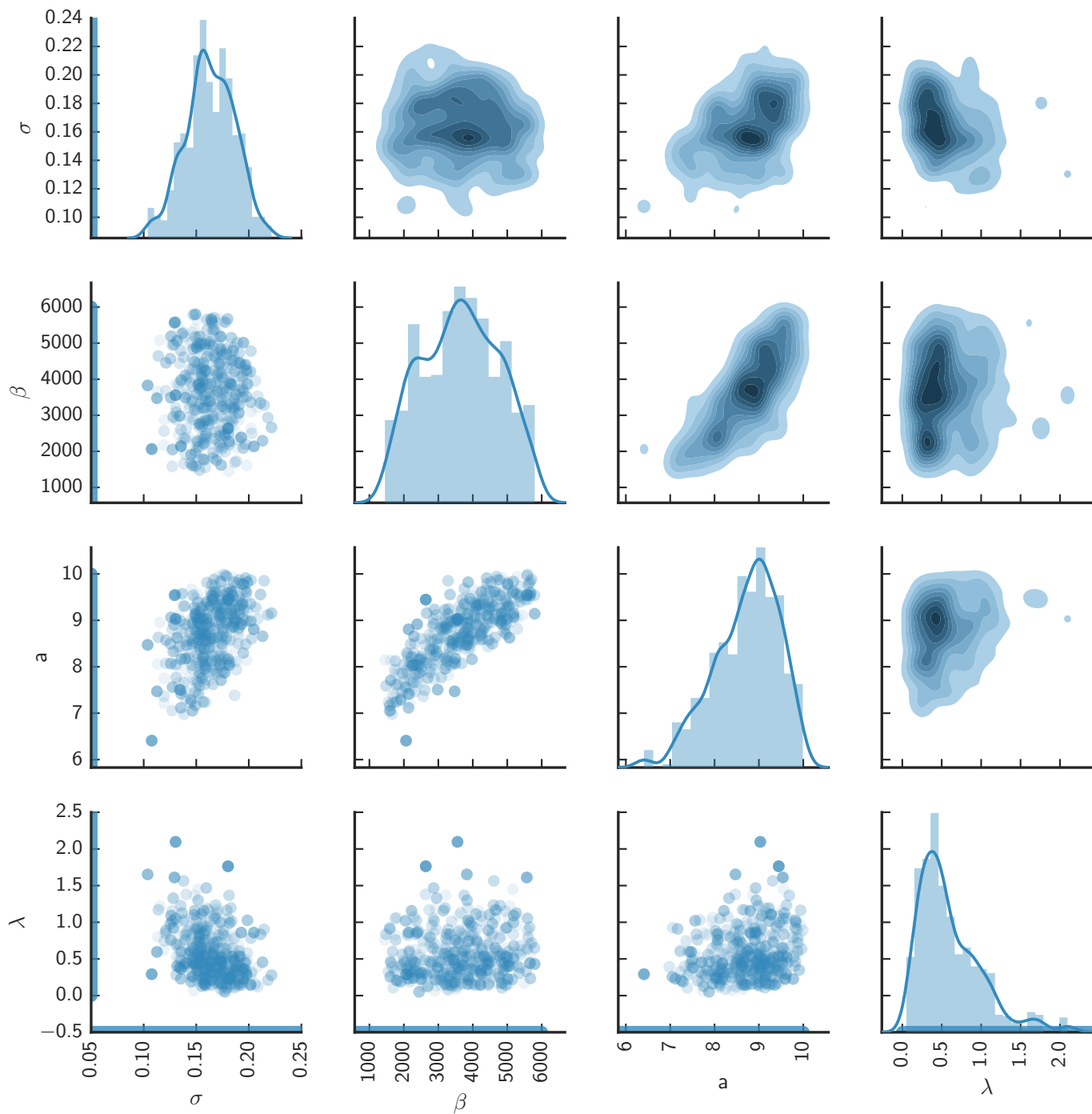

Supplement: S3 Fig — Marginal posterior parameter distributions computed from 1,000 samples. Blue shaded ranges along the axes show the intervals within which parameters were allowed to vary during calibration. (PDF) [file pmed.1002509.s003.pdf]

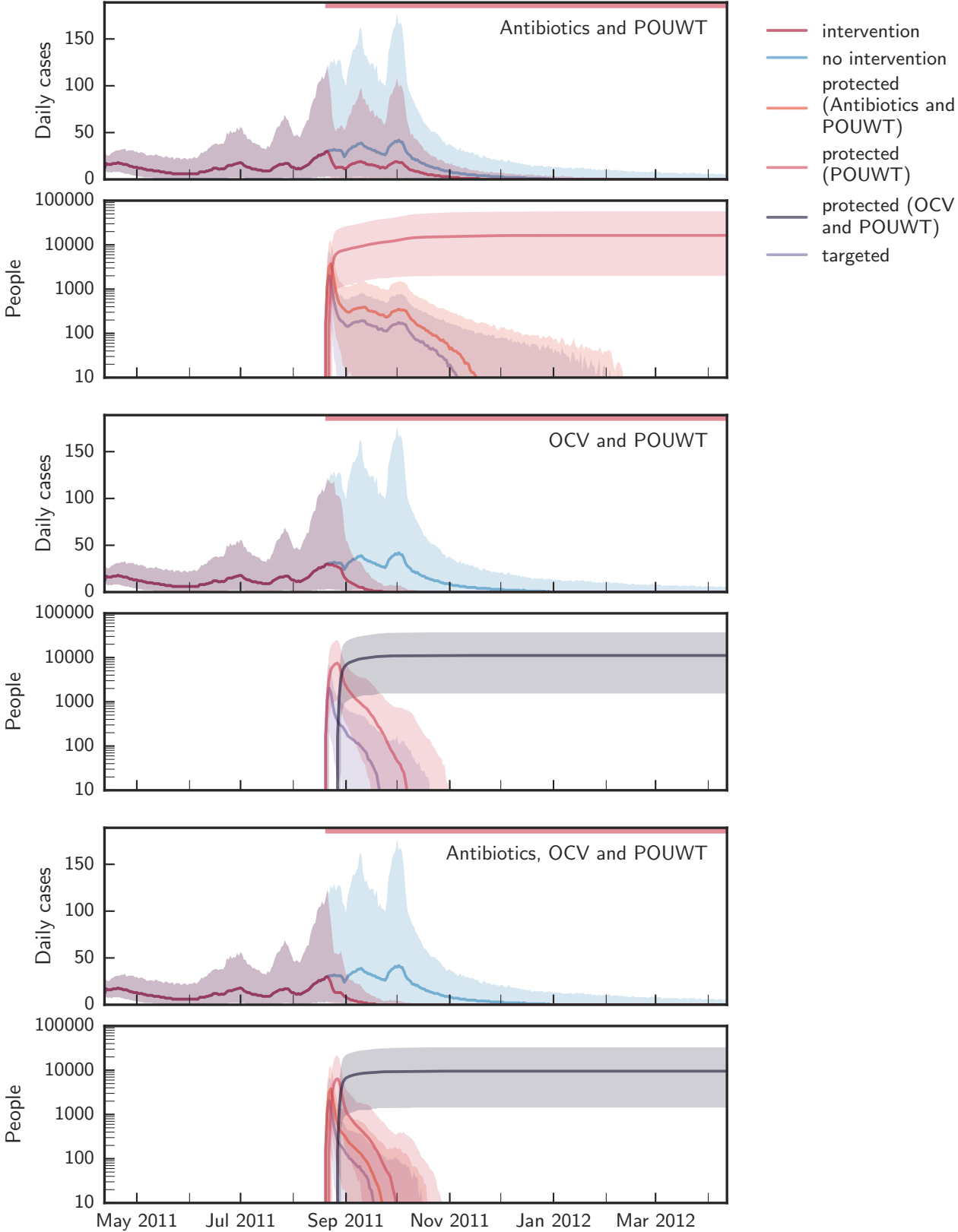

Supplement: S4 Fig — The lower panels in each pair of panels show the number of people targeted during each timestep and the number of people protected by the interventions. Solid lines show the median over all simulations, shaded areas the 2.5th and 97.5th percentiles. The red bar at the top of each panel marks the period during which interventions are applied. (PDF) [file pmed.1002509.s004.pdf]

Cases averted

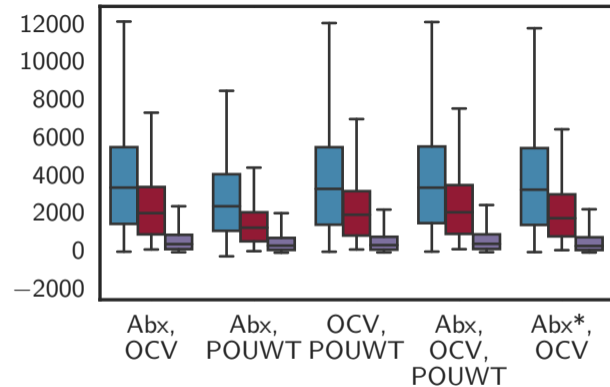

Persons targeted

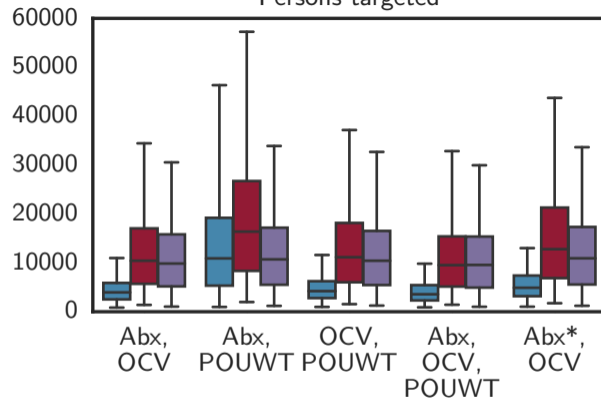

Clusters targeted

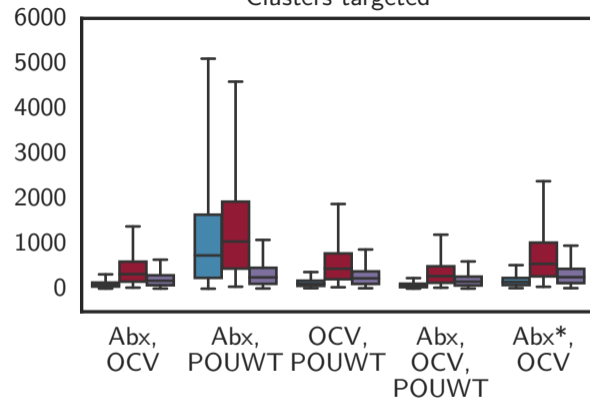

early peak late

Supplement: S5 Fig — Boxplots of the number of averted cases, the number of targeted persons, and the number of targeted clusters predicted by the model for combinations of the 3 main intervention types with case-area targeted allocation in a 100-m radius starting at 3 different times. Abx stands for antibiotics. Abx* stands for administering antibiotics only within a range of 15 m, while OCV is administered within the whole cluster. Whiskers mark the 2.5th and 97.5th percentiles. (PDF) [file pmed.1002509.s005.pdf]

City-wide mass campaign

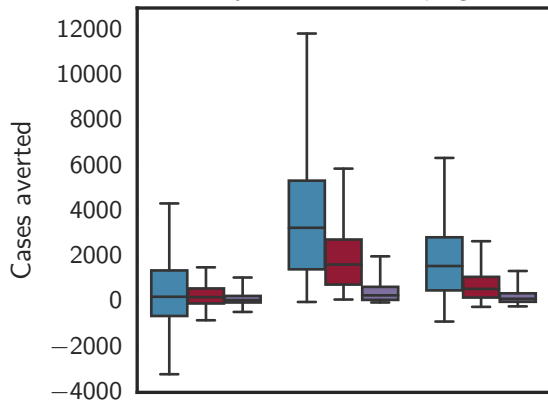

District-targeted mass campaign

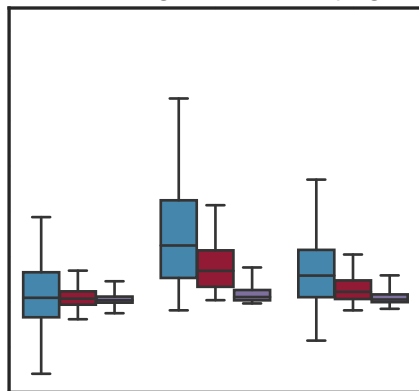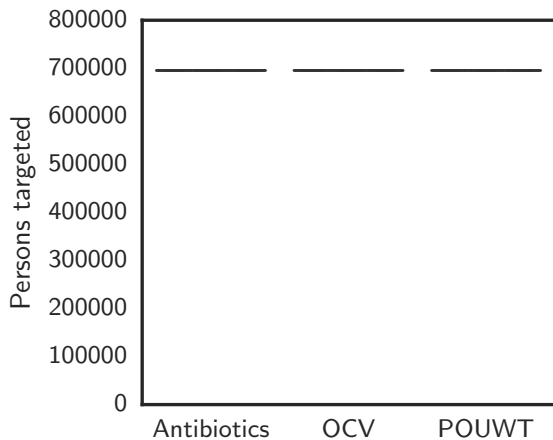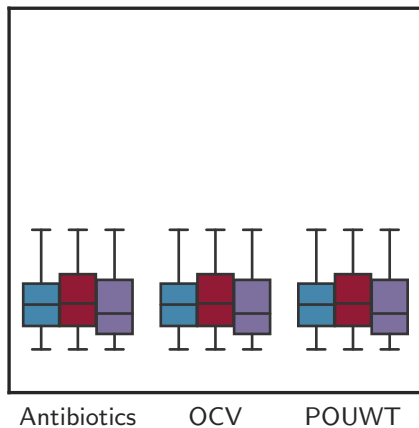

early peak late

Supplement: S6 Fig — Mass intervention campaigns targeting 70% of the entire city population (left column) and 70% of the people living in the 3 districts with the highest attack rate at the onset of the interventions (right column). Boxplots show the number of averted cases (top row) and the number of targeted persons (bottom row) predicted by the model. Whiskers mark the 2.5th and 97.5th percentiles. Boxplots of the number of targeted persons in a mass intervention campaign of the entire city collapse because the number of people to target was fixed. (PDF) [file pmed.1002509.s006.pdf]

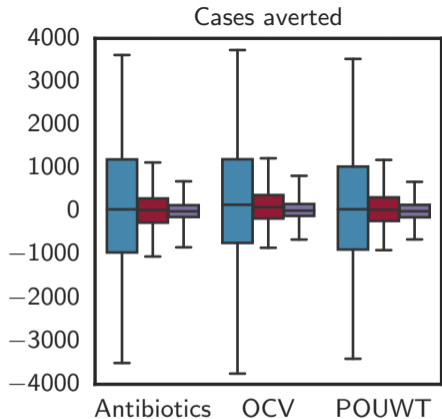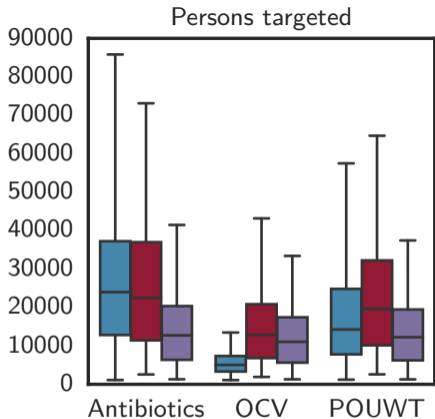

early peak late

Supplement: S7 Fig — Boxplots of the number of averted cases and the number of targeted persons predicted by the model. The number of targeted persons has been fixed to the same values for case-area targeted allocation with a 100-m radius (Fig 5). Whiskers mark the 2.5th and 97.5th percentiles. (PDF) [file pmed.1002509.s007.pdf]

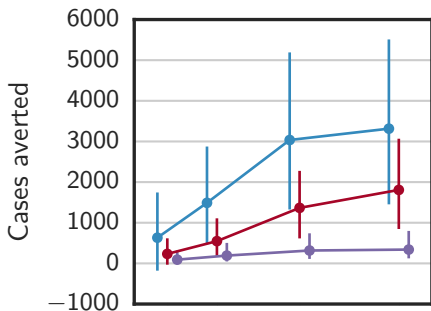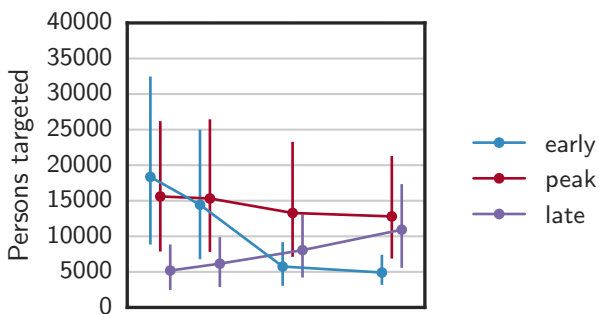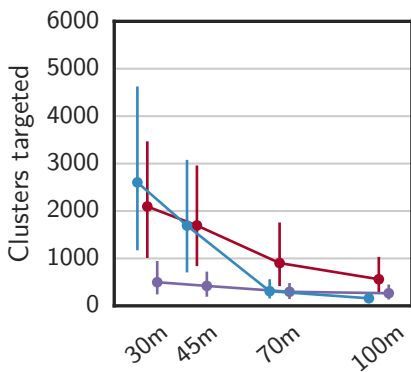

Supplement: S8 Fig — The number of averted cases, the number of targeted persons, and the number of targeted clusters predicted by the model with case-area targeted allocation and variable radius, starting at 3 different times. The error bars cover the range between the 25th and 75th quantile over all simulations. (PDF) [file pmed.1002509.s008.pdf]

Cases averted

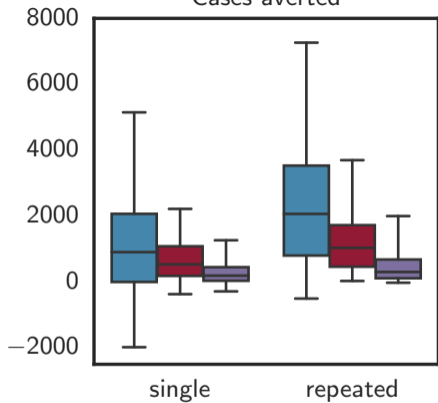

Persons targeted

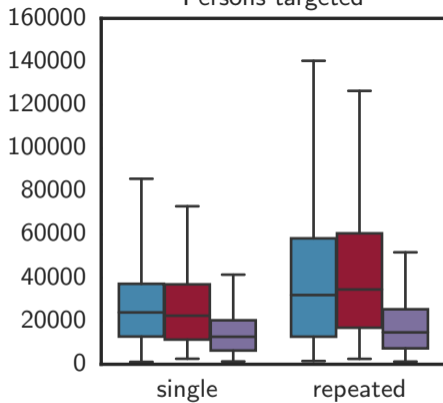

Clusters targeted

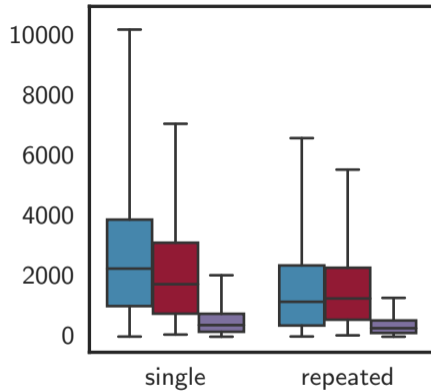

early peak late

Supplement: S9 Fig — Boxplots of the number of averted cases, the number of targeted persons, and the number of targeted clusters predicted by the model for 2 different strategies of allocating antibiotics in CATIs within a 100-m radius starting at 3 different times. Whiskers mark the 2.5th and 97.5th percentiles. Single allocation designates the standard mode, where every person can receive 1 dose of antibiotics only once during the epidemic. Repeated allocation designates a mode where a person can get antibiotics several times, with a minimal interval of 2 weeks, if he/she lives within the intervention radius of several cases. (PDF) [file pmed.1002509.s009.pdf]

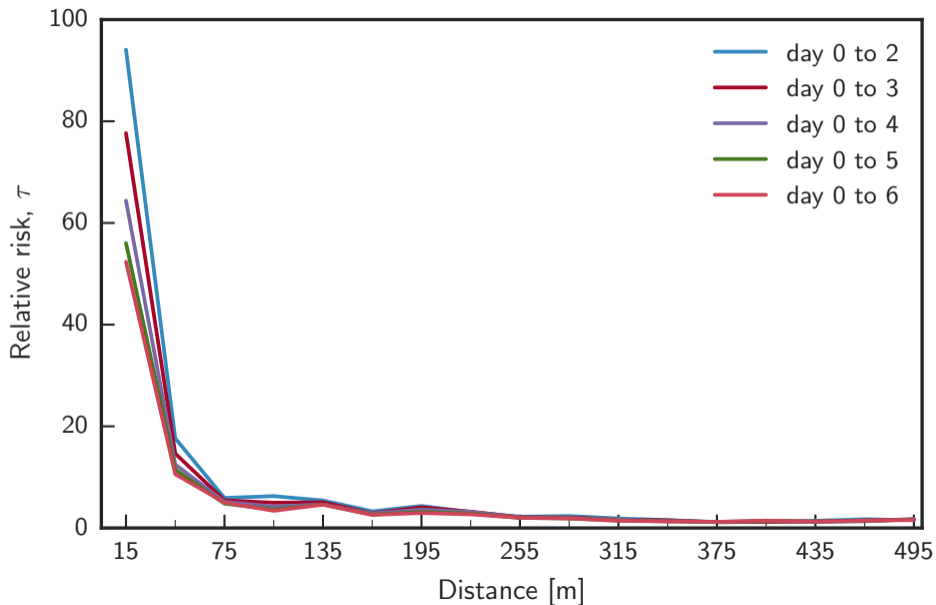

Supplement: S10 Fig — (PDF) [file pmed.1002509.s010.pdf]

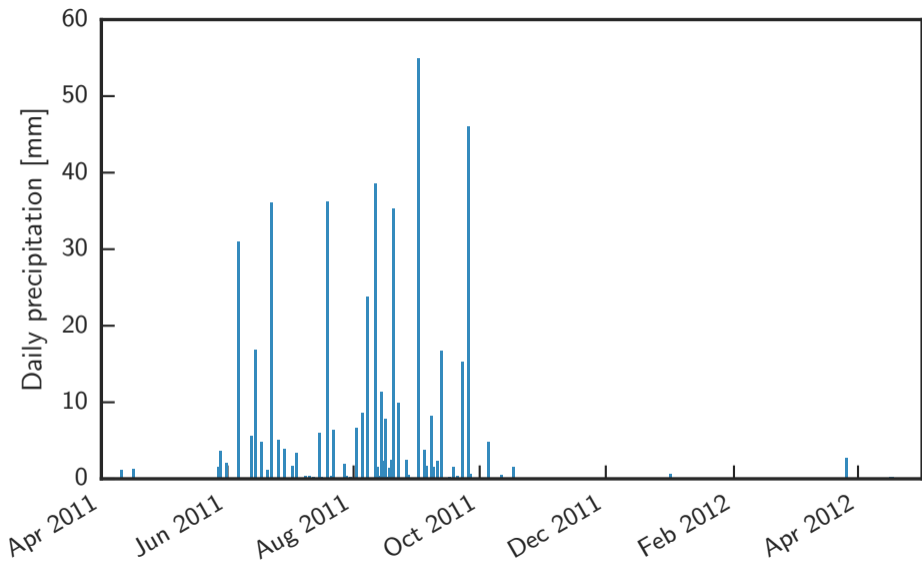

Supplement: S11 Fig — (PDF) [file pmed.1002509.s011.pdf]

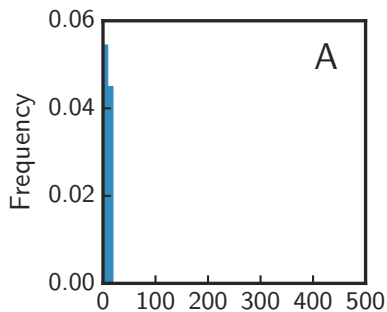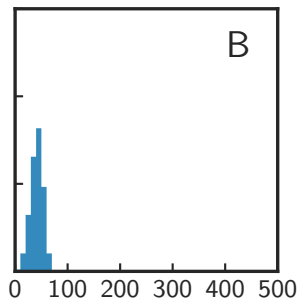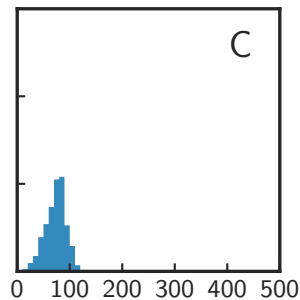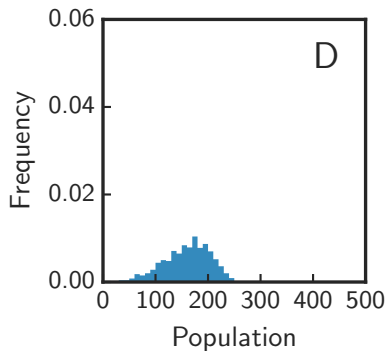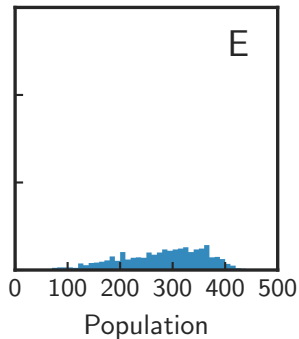

Supplement: S12 Fig — Histograms of the number of people living within a circle with radius 15 m (A), 30 m (B), 45 m (C), 70 m (D), and 100 m (E) in N’Djamena obtained by sampling the population distribution at 1,000 random points. The bin width in the histograms is 10. (PDF) [file pmed.1002509.s012.pdf]

15

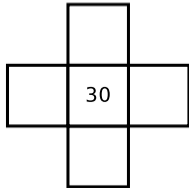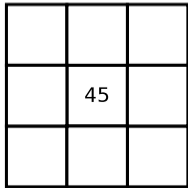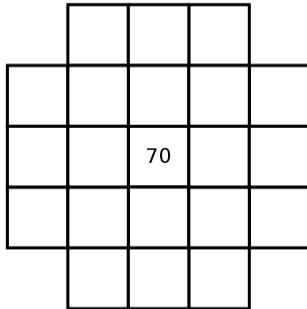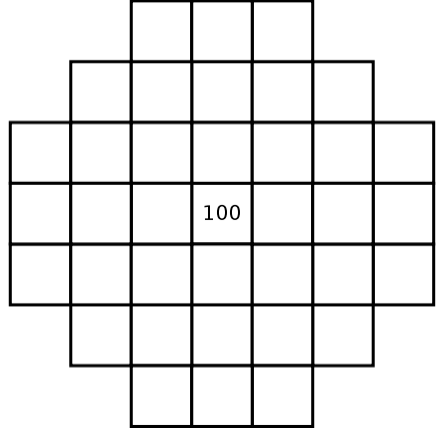

Supplement: S13 Fig — The side length of each square is 30 m. (PDF) [file pmed.1002509.s013.pdf]

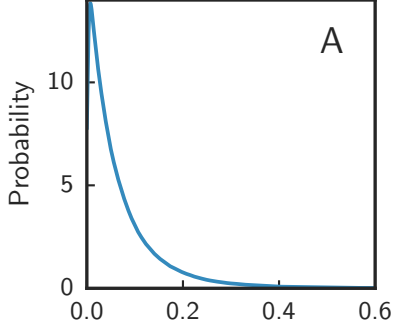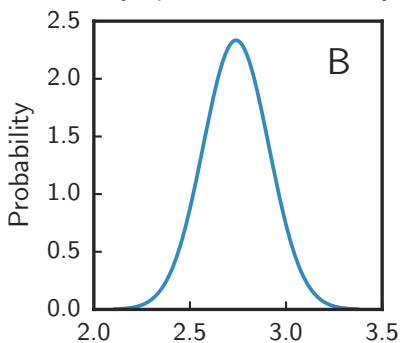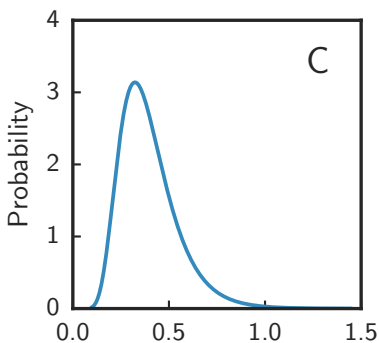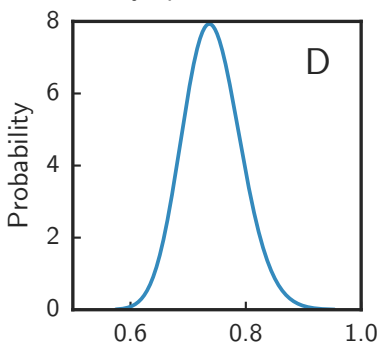

Supplement: S14 Fig — Intervention parameters related to antibiotics (reduction of symptomatic fraction [A] and reduction of duration of shedding [B]), OCV (reduction of symptomatic fraction [C]), and POUWT (reduction of exposure [D]). (A) was obtained from Lewnard et al. [49] directly, whereas (B) (normal), (C) (log-normal), and (D) (log-normal) were fitted to the corresponding confidence intervals given in Table 1. (PDF) [file pmed.1002509.s014.pdf]

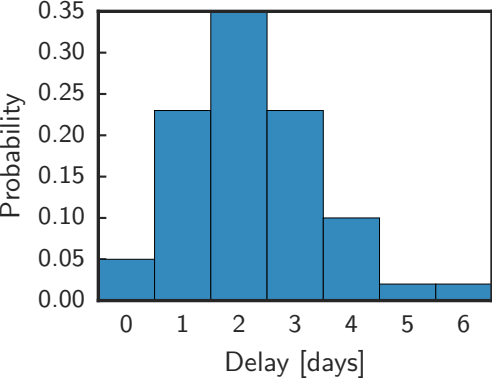

Supplement: S15 Fig — Probability distribution of the delay (in days) between the onset of symptoms of the initial case in a cluster and the deployment of an intervention team. (PDF) [file pmed.1002509.s015.pdf]

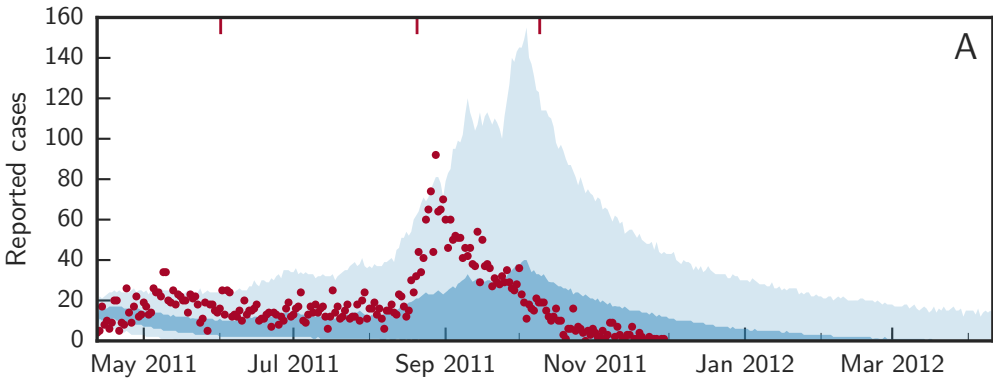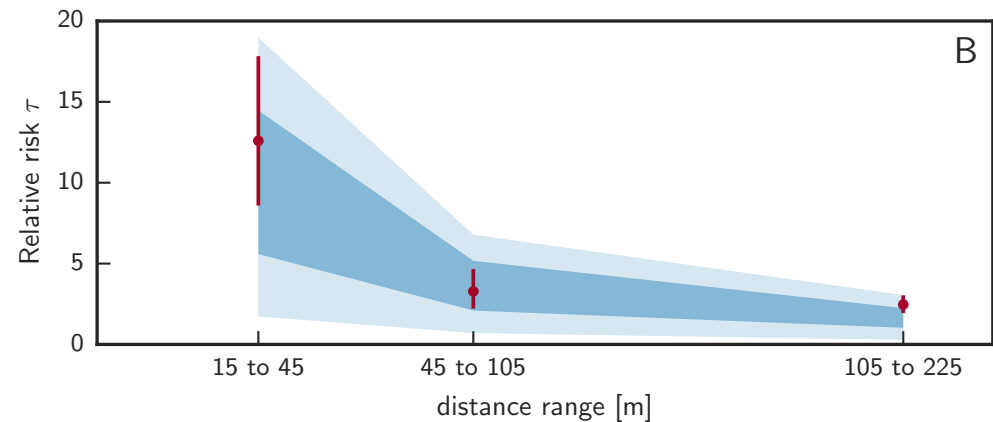

Supplement: S16 Fig — (A) shows the distribution of daily incident cholera cases from uncontrolled epidemic simulations. The shaded areas represent the marginal interquartile range (dark blue) and the 2.5th and 97.5th percentiles (light blue) from 1,000 simulated epidemics, with the true number of daily reported cases shown as red dots. Red ticks at the top of (A) represent the 3 times when interventions start. (B) shows the interquartile range (dark blue) and 2.5th and 97.5th posterior percentiles (light blue) of the relative risk (τ statistic) of the next case being within a specific distance from a case within 5 days of his/her symptom onset. Red dots and bars (95% confidence intervals) represent the computed τ from the data. (PDF) [file pmed.1002509.s016.pdf]

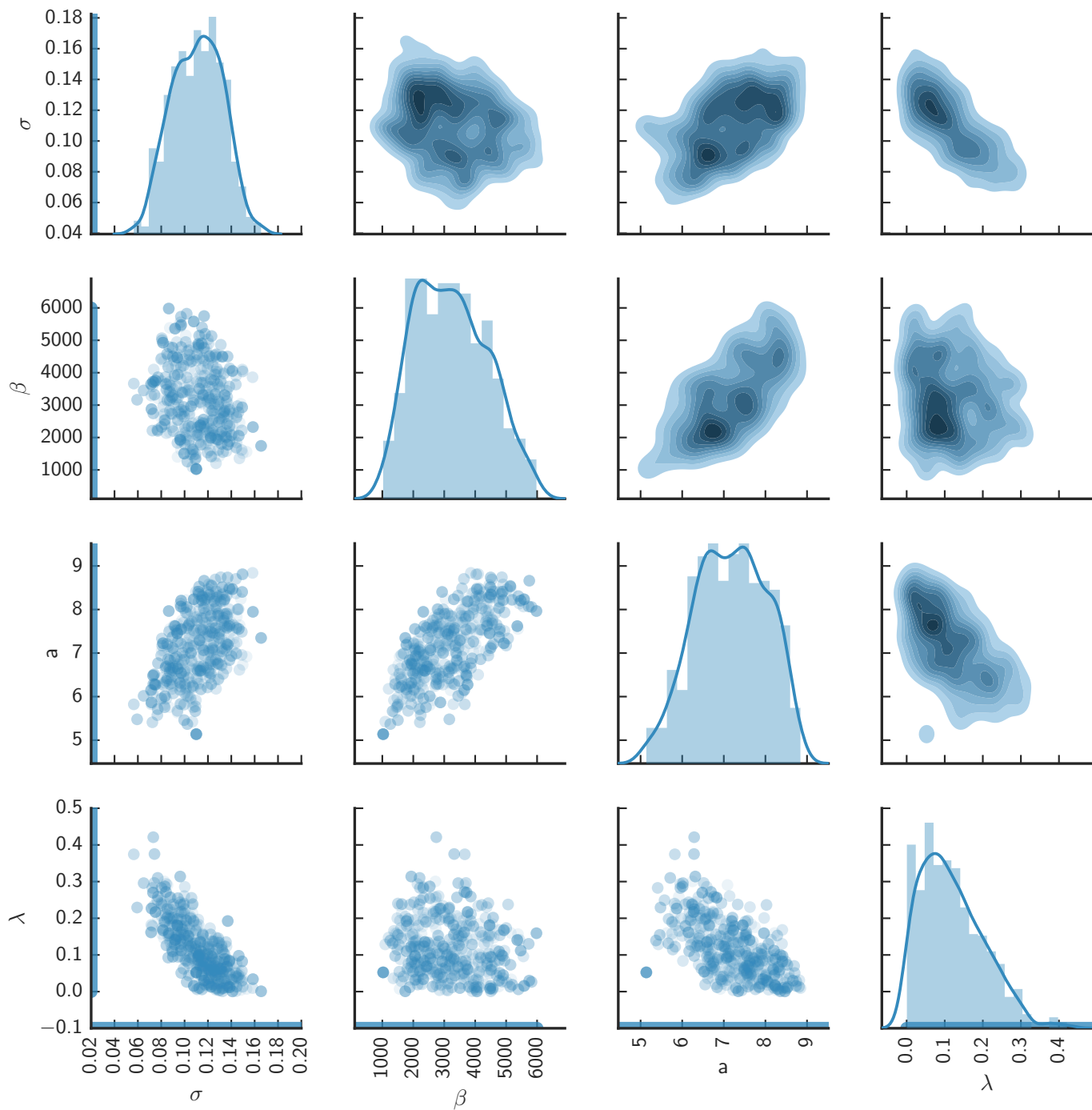

Supplement: S17 Fig — Marginal posterior parameter distributions computed from 1,000 samples. Blue shaded ranges along the axes show the intervals within which parameters were allowed to vary during calibration. (PDF) [file pmed.1002509.s017.pdf]

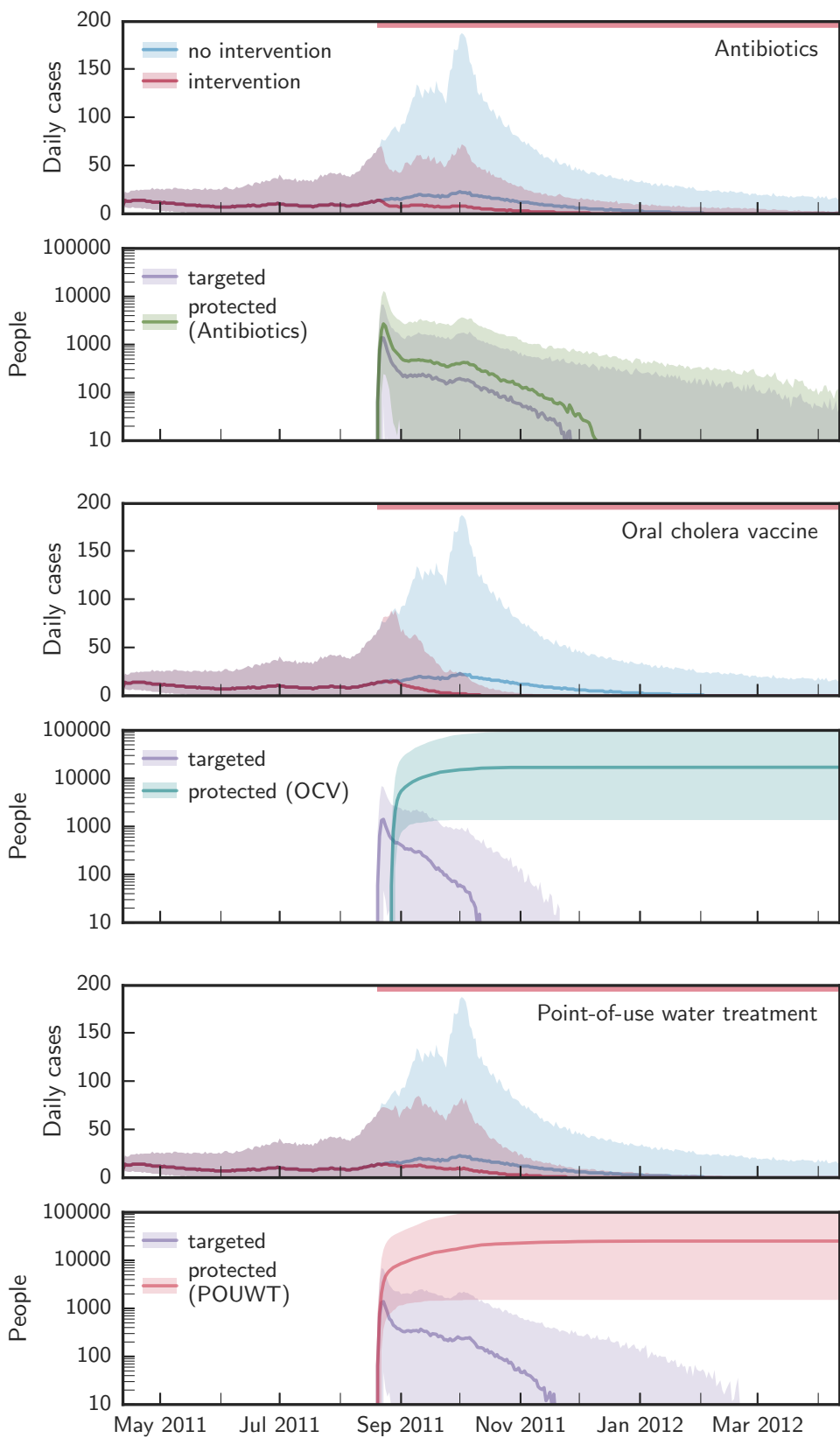

Supplement: S18 Fig — Upper panels in each pair of panels show the evolution of the epidemics simulated including long-distance transmission without intervention and with case-area targeted allocation of antibiotics, OCV, and POUWT within a 100-m radius starting at the epidemic peak. Lower panels in each pair of panels show the corresponding number of people targeted during each timestep and the number of people protected by each intervention. Solid lines designate the median over all simulations, shaded areas the 2.5th and 97.5th percentiles. The red bars at the top of the panels mark the period during which interventions were applied. (PDF) [file pmed.1002509.s018.pdf]

Cases averted

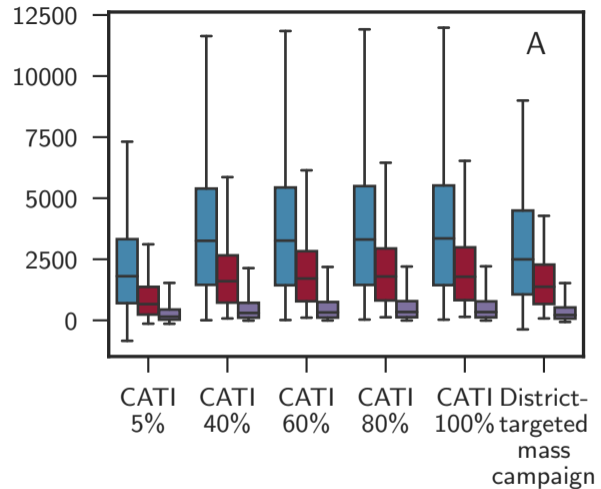

Persons targeted

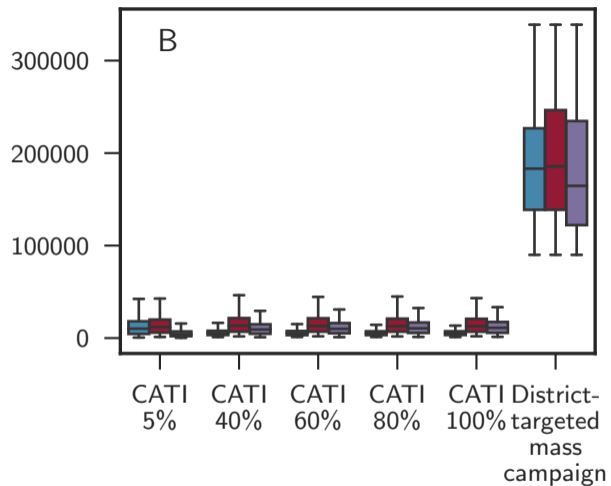

Clusters targeted

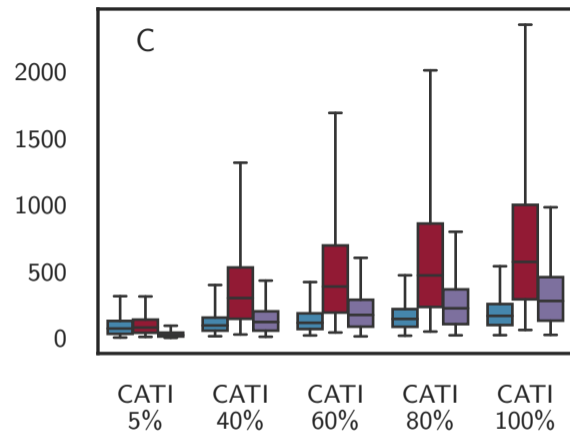

early peak late

Supplement: S20 Fig — Boxplots of the number of averted cases, the number of targeted persons, and the number of targeted clusters predicted when targeting CATIs using OCV within 100 m around 100%, 80%, 60%, 40%, and 5% of symptomatic cases and with district-targeted mass campaigns. Colors denote campaigns starting at 3 different times. Whiskers mark the 2.5th and 97.5th percentiles. (PDF) [file pmed.1002509.s020.pdf]

Reduction of epidemic duration [days]

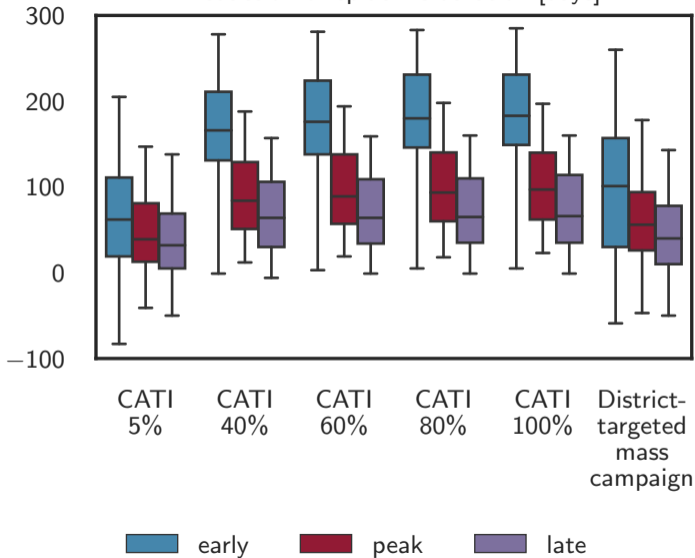

Supplement: S21 Fig — Boxplots of the number of epidemic days reduced predicted when targeting CATIs using OCV within 100 m around 100%, 80%, 60%, 40%, and 5% of symptomatic cases and with district-targeted mass campaigns. Colors denote campaigns starting at 3 different times. Whiskers mark the 2.5th and 97.5th percentiles. (PDF) [file pmed.1002509.s021.pdf]

Cases averted

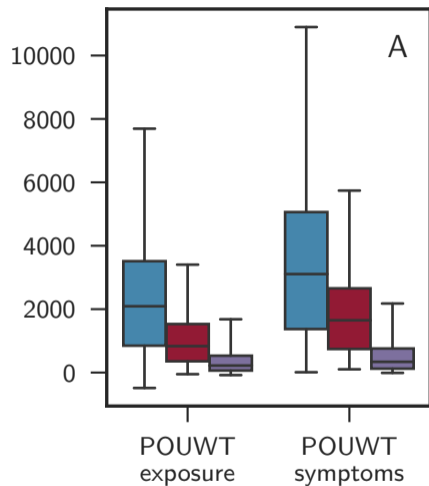

Persons targeted

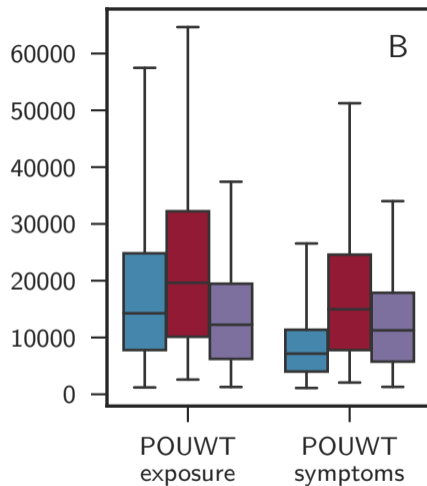

Clusters targeted

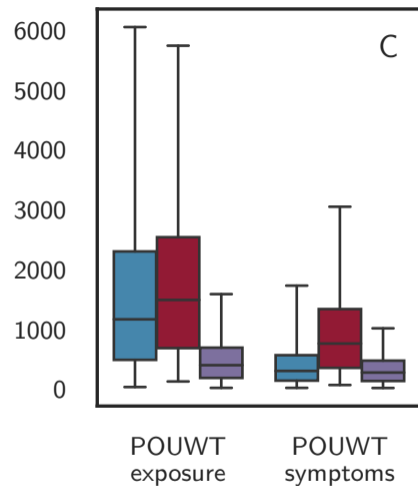

Supplement: S22 Fig — Boxplots of the number of averted cases, the number of targeted persons, and the number of targeted clusters predicted when implementing CATIs using POUWT in a 100-m radius around reported cases through reduction of the likelihood of getting infected (mechanism of our main analysis) or through reduction of the likelihood of getting symptoms (mechanism in sensitivity analysis). Colors denote campaigns starting at 3 different times. Whiskers mark the 2.5th and 97.5th percentiles. (PDF) [file pmed.1002509.s022.pdf]

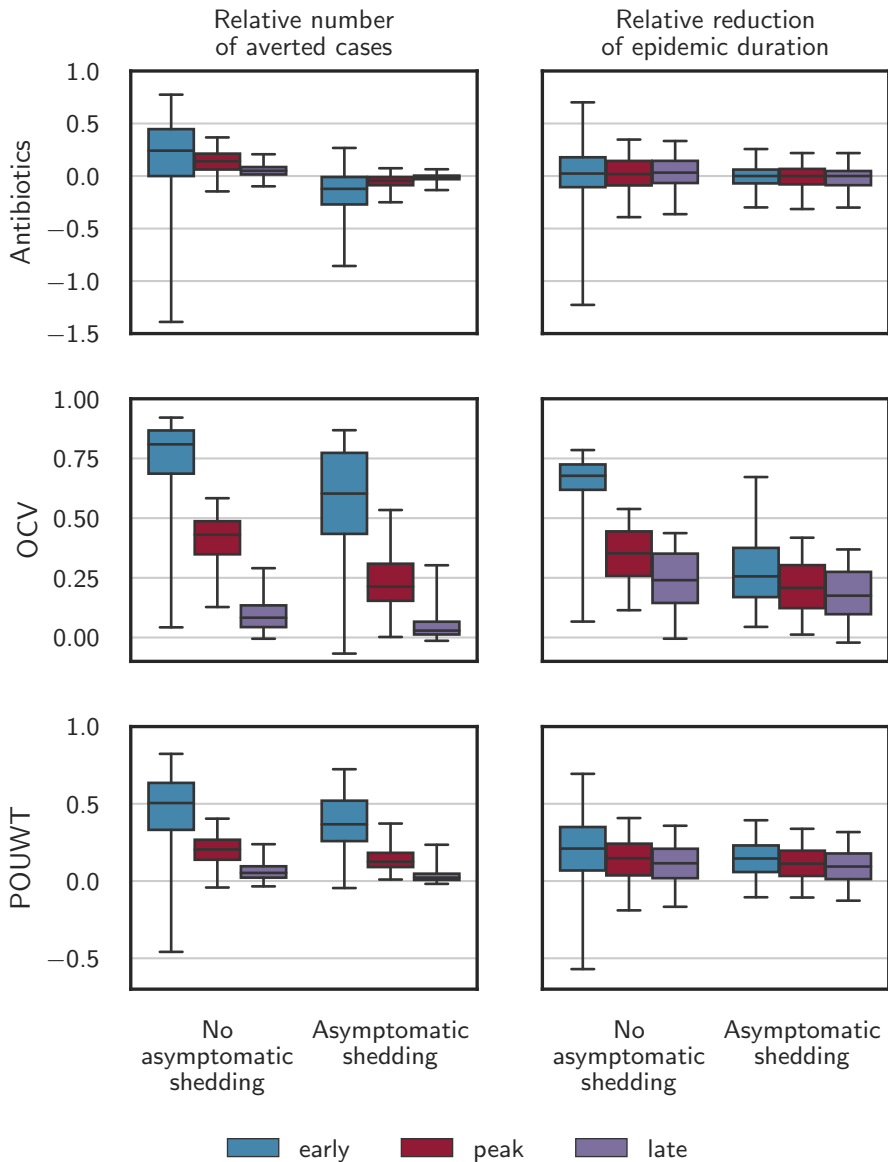

Supplement: S23 Fig — Boxplots of the relative number of averted cases (averted cases divided by number of cases without intervention) and the relative number of epidemic days reduced (number of epidemic days reduced divided by the number of epidemic days without intervention) with asymptomatic individuals being 10% as infectious as symptomatic individuals, compared to the results of our main analysis (no shedding by asymptomatic individuals), with different CATIs in a 100-m radius. Colors denote campaigns starting at 3 different times. Whiskers mark the 2.5th and 97.5th percentiles. (PDF) [file pmed.1002509.s023.pdf]
