# Supplementary material for: The potential impact of case-area targeted interventions in response to cholera outbreaks: A modeling study
Source: PLoS Med. 2018 Feb 27;15(2):e1002509. doi: 10.1371/journal.pmed.1002509 (PMC5828347; doi:10.1371/journal.pmed.1002509)
Supplement: S19 Fig — Boxplots of the number of averted cases, the number of targeted persons, and the number of targeted clusters predicted by 2 models without and with 5% long-distance transmission events and by of allocating OCV, antibiotics, and POUWT in CATIs within a 100-m radius starting at 3 different times. Whiskers mark the 2.5th and 97.5th percentiles. (PDF) [file pmed.1002509.s019.pdf]

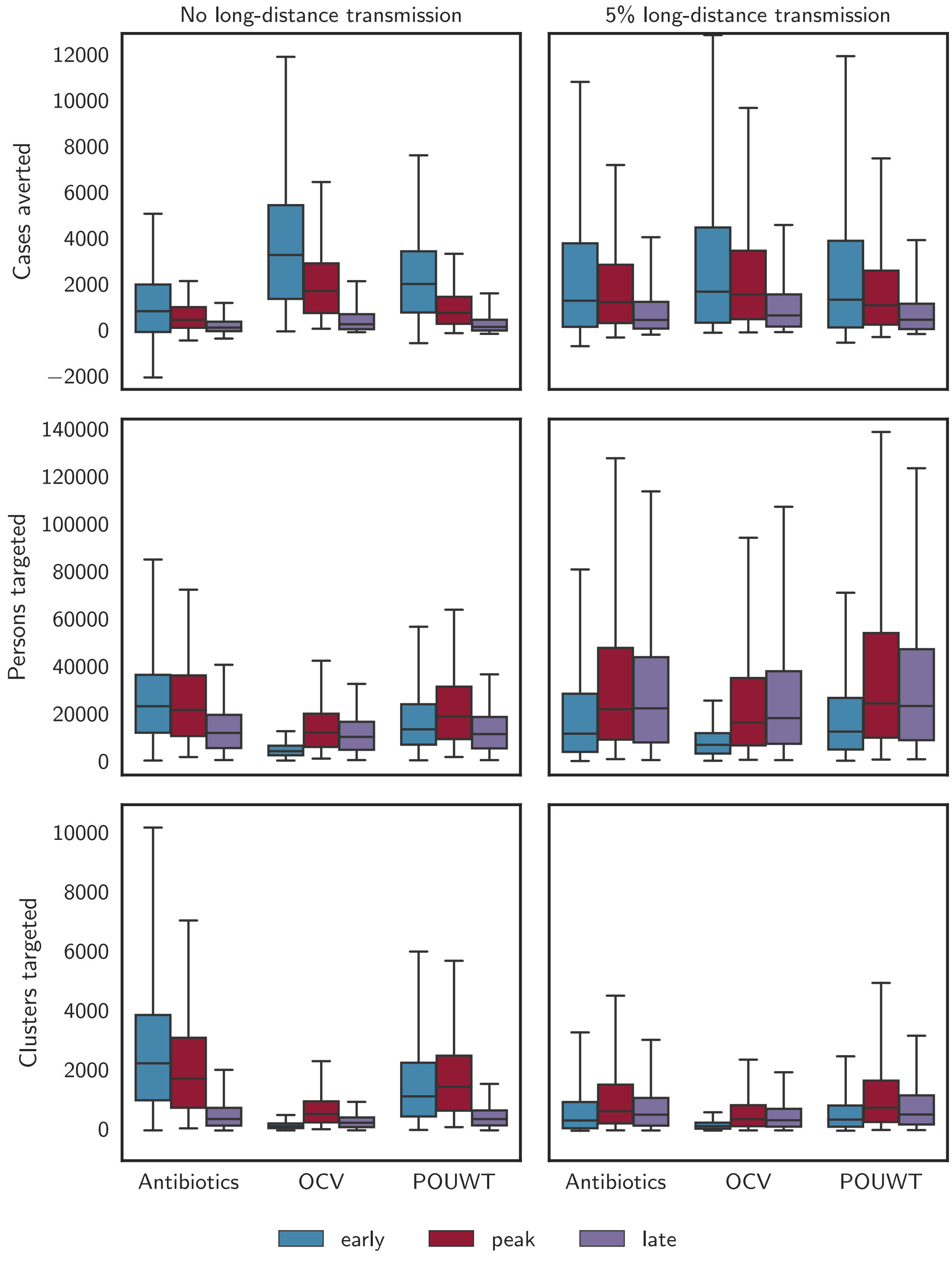

No long-distance transmission
5% long-distance transmission
Cases averted
12000
10000
8000
6000
4000
2000
0
−2000
Persons targeted
140000
120000
100000
80000
60000
40000
20000
0
Clusters targeted
10000
8000
6000
4000
2000
0
Antibiotics
OCV
POUWT
early
peak
late
