# Supplementary material for: The potential impact of case-area targeted interventions in response to cholera outbreaks: A modeling study
Source: PLoS Med. 2018 Feb 27;15(2):e1002509. doi: 10.1371/journal.pmed.1002509 (PMC5828347; doi:10.1371/journal.pmed.1002509)
Supplement: S1 Table — The table shows all intervention scenarios considered, specifically the type of intervention, the timing, and the type of allocation, together with median values (2.5% and 97.5% percentiles in brackets) of the number of targeted clusters, the total number of targeted people, and the total number of averted cases computed using 1,000 model simulations. *Every person can get antibiotics only once during the epidemic. **Every person can get antibiotics several times with a minimum delay of 2 weeks between 2 administrations. (PDF) [file pmed.1002509.s024.pdf]

	Antibiotics*	Antibiotics**	OCV	POUWT	Clusters 100m	Clusters 70m	Clusters 45m	Clusters 30m	Clusters 15m	Random	Entire population	By district	Early	Peak	Late	Clusters targeted	People targeted	Cases averted
1	x				x								x			2282 [21, 10229]	23987 [1109, 85818]	905 [-1984, 5157]
2	x				x									x		1763 [87, 7098]	22477 [2539, 73165]	532 [-371, 2220]
3	x				x										x	404 [18, 2072]	12735 [1288, 41564]	191 [-299, 1267]
4		x			x								x			1179 [24, 6671]	32027 [1501, 140503]	2065 [-506, 7300]
5		x			x									x		1289 [62, 5573]	34596 [2479, 126474]	1034 [28, 3698]
6		x			x										x	301 [17, 1308]	14760 [1248, 51802]	302 [-24, 2000]
7			x		x								x			166 [20, 539]	5044 [1083, 13489]	3353 [26, 12006]
8			x		x									x		571 [59, 2360]	12862 [1915, 43188]	1784 [139, 6539]
9			x		x										x	278 [23, 982]	11064 [1291, 33596]	338 [-9, 2227]
10				x	x								x			1167 [36, 6053]	14263 [1200, 57750]	2091 [-494, 7697]
11				x	x									x		1491 [128, 5747]	19632 [2590, 64664]	833 [-51, 3404]
12				x	x										x	401 [20, 1590]	12249 [1267, 37495]	221 [-80, 1683]
13	x					x							x			1371 [18, 6635]	13962 [783, 51868]	1917 [-738, 7021]
14	x					x								x		1363 [76, 5760]	16024 [1425, 56816]	943 [-15, 3499]
15	x					x									x	378 [19, 1870]	9022 [775, 29234]	213 [-159, 1398]
16		x				x							x			985 [20, 5716]	19376 [916, 93989]	2285 [-291, 8244]
17		x				x								x		1207 [79, 5075]	24803 [2100, 88682]	1162 [24, 3936]
18		x				x									x	321 [20, 1520]	10456 [873, 41415]	284 [-51, 1785]
19			x			x							x			314 [33, 1855]	5474 [1013, 22979]	2986 [0, 11090]
20			x			x								x		930 [86, 4212]	13707 [1738, 48912]	1337 [87, 5333]
21			x			x									x	300 [24, 1056]	8137 [875, 25731]	315 [-11, 2096]
22				x		x							x			1231 [39, 6489]	12723 [1047, 53439]	2037 [-671, 7231]
23				x		x								x		1442 [104, 5709]	16447 [1895, 55113]	813 [-24, 3413]
24				x		x									x	403 [28, 1737]	8882 [897, 29411]	197 [-91, 1615]
25	x						x						x			309 [22, 1836]	3633 [427, 16925]	3028 [2, 10753]
26	x						x							x		738 [50, 3218]	8289 [841, 31695]	1677 [122, 5727]

	Antibiotics*	Antibiotics**	OCV	POUWT	Clusters 100m	Clusters 70m	Clusters 45m	Clusters 30m	Clusters 15m	Random	Entire population	By district	Early	Peak	Late	Clusters targeted	People targeted	Cases averted
27	x						x								x	277 [17, 1104]	4805 [428, 16353]	339 [-9, 2116]
28		x					x						x			285 [21, 1775]	4236 [455, 22493]	3147 [4, 10933]
29		x					x							x		727 [50, 2867]	10297 [859, 37559]	1690 [114, 5651]
30		x					x								x	260 [18, 1082]	5107 [427, 18789]	346 [-11, 2027]
31			x				x						x			1846 [55, 7972]	15018 [838, 59912]	1428 [-1213, 6344]
32			x				x							x		1768 [158, 6833]	15836 [1828, 55569]	504 [-272, 2802]
33			x				x								x	432 [30, 1718]	6097 [552, 21097]	187 [-96, 1659]
34				x			x						x			1636 [41, 8401]	13040 [652, 58138]	1551 [-964, 5924]
35				x			x							x		1683 [126, 6924]	14468 [1306, 52742]	584 [-212, 2719]
36				x			x								x	429 [21, 1748]	5848 [475, 19915]	180 [-134, 1422]
37	x							x					x			246 [20, 1654]	2489 [296, 13447]	3135 [11, 11105]
38	x							x						x		721 [51, 3163]	7013 [655, 28443]	1674 [114, 5961]
39	x							x							x	253 [17, 938]	3361 [285, 11443]	368 [-6, 2327]
40		x						x					x			244 [19, 1532]	2768 [283, 15127]	3108 [15, 11030]
41		x						x						x		706 [54, 3125]	7512 [659, 29943]	1643 [114, 5907]
42		x						x							x	255 [16, 991]	3462 [295, 12188]	361 [-1, 2204]
43			x					x					x			2860 [63, 10839]	20125 [690, 70958]	469 [-2730, 4531]
44			x					x						x		2209 [174, 8049]	16090 [1363, 56277]	185 [-667, 1776]
45			x					x							x	537 [35, 2219]	5439 [426, 18911]	80 [-340, 1083]
46				x				x					x			2034 [57, 9769]	14356 [595, 64138]	1000 [-1500, 5147]
47				x				x						x		1903 [177, 7341]	13959 [1500, 47698]	392 [-371, 2233]
48				x				x							x	475 [24, 1993]	4949 [342, 16945]	130 [-191, 1276]
49	x								x				x			2708 [84, 11191]	12064 [409, 47077]	538 [-2866, 4538]
50	x								x					x		2096 [172, 7775]	9375 [772, 33680]	211 [-611, 1636]
51	x								x						x	546 [28, 2453]	2516 [147, 10247]	72 [-351, 1087]
52		x							x				x			2737 [88, 10912]	11921 [411, 49021]	501 [-2404, 4623]

	Antibiotics*	Antibiotics**	OCV	POUWT	Clusters 100m	Clusters 70m	Clusters 45m	Clusters 30m	Clusters 15m	Random	Entire population	By district	Early	Peak	Late	Clusters targeted	People targeted	Cases averted
53		x							x					x		2094 [176, 7871]	9368 [872, 34746]	205 [-605, 1739]
54		x							x						x	554 [30, 2298]	2510 [151, 9975]	78 [-405, 1143]
55			x						x				x			3457 [98, 12756]	15436 [412, 53544]	-8 [-3582, 3217]
56			x						x					x		2424 [188, 8580]	10651 [822, 36913]	-3 [-1056, 1176]
57			x						x						x	632 [34, 2937]	2846 [161, 12352]	0 [-706, 698]
58				x					x				x			3403 [108, 12318]	15006 [480, 52809]	30 [-3292, 3287]
59				x					x					x		2398 [219, 8483]	10383 [995, 37653]	35 [-1052, 1148]
60				x					x						x	657 [34, 2951]	2960 [147, 12097]	-1 [-834, 733]
61	x		x		x								x			94 [11, 328]	3957 [843, 10987]	3425 [38, 12203]
62	x		x		x									x		332 [33, 1390]	10431 [1393, 34495]	2076 [153, 7383]
63	x		x		x										x	186 [13, 656]	9855 [1042, 30585]	439 [9, 2455]
64	x			x	x								x			747 [10, 5139]	10908 [976, 46386]	2438 [-208, 8552]
65	x			x	x									x		1055 [53, 4606]	16385 [1980, 57266]	1307 [64, 4481]
66	x			x	x										x	261 [15, 1095]	10710 [1189, 33937]	359 [-15, 2076]
67			x	x	x								x			122 [20, 380]	4217 [981, 11607]	3358 [34, 12132]
68			x	x	x									x		453 [42, 1887]	11147 [1539, 37172]	1991 [152, 7046]
69			x	x	x										x	240 [19, 882]	10432 [1247, 32787]	386 [0, 2293]
70	x		x	x	x								x			77 [12, 245]	3573 [897, 9838]	3422 [41, 12197]
71	x		x	x	x									x		287 [32, 1209]	9531 [1428, 32858]	2120 [169, 7613]
72	x		x	x	x										x	167 [12, 614]	9570 [1009, 30049]	454 [9, 2510]
73			x								x		x			-	695445 [695445, 695445]	3279 [-2, 11890]
74			x								x			x		-	695445 [695445, 695445]	1653 [105, 5904]
75			x								x				x	-	695445 [695445, 695445]	291 [-24, 2021]
76				x							x		x			-	695445 [695445, 695445]	1588 [-893, 6371]
77				x							x			x		-	695445 [695445, 695445]	577 [-218, 2688]
78				x							x				x	-	695445 [695445, 695445]	141 [-198, 1378]

	Antibiotics*	Antibiotics**	OCV	POUWT	Clusters 100m	Clusters 70m	Clusters 45m	Clusters 30m	Clusters 15m	Random	Entire population	By district	Early	Peak	Late	Clusters targeted	People targeted	Cases averted
79			x									x	x			-	183180 [89966, 338855]	2496 [-374, 9000]
80			x									x		x		-	185556 [89966, 338855]	1374 [75, 4282]
81			x									x			x	-	164586 [89966, 338855]	217 [-67, 1535]
82				x								x	x			-	183180 [89966, 338855]	1165 [-1718, 5410]
83				x								x		x		-	185556 [89966, 338855]	446 [-373, 2099]
84				x								x			x	-	164586 [89966, 338855]	107 [-307, 1174]
85			x	x								x	x			-	183180 [89966, 338855]	2545 [-277, 9572]
86			x	x								x		x		-	185556 [89966, 338855]	1437 [94, 4571]
87			x	x								x			x	-	164586 [89966, 338855]	250 [-85, 1684]
88	x*		x		x				x				x			159 [21, 532]	4908 [1031, 13116]	3315 [14, 11893]
89	x*		x		x				x					x		560 [51, 2395]	12796 [1736, 43734]	1808 [118, 6505]
90	x*		x		x				x						x	266 [21, 964]	10928 [1202, 33704]	340 [-4, 2282]
91	x*		x			x			x				x			309 [25, 1866]	5751 [855, 22897]	3036 [-8, 10748]
92	x*		x			x			x					x		902 [79, 4319]	13280 [1664, 51063]	1366 [69, 5347]
93	x*		x			x			x						x	296 [22, 1111]	8052 [825, 25865]	317 [-12, 2112]
94	x*		x				x		x				x			1692 [58, 8145]	14440 [684, 60945]	1490 [-1160, 6348]
95	x*		x				x		x					x		1694 [145, 6883]	15321 [1419, 55727]	547 [-209, 2768]
96	x*		x				x		x						x	420 [20, 1668]	6152 [432, 19921]	192 [-77, 1662]
97	x*		x					x	x				x			2603 [69, 10724]	18356 [718, 69874]	632 [-2455, 4276]
98	x*		x					x	x					x		2095 [170, 7662]	15603 [1503, 54376]	233 [-516, 1716]
99	x*		x					x	x						x	498 [24, 2301]	5188 [379, 19053]	92 [-264, 1221]
100	x									x			x			-	23987 [1109, 85818]	45 [-3509, 3616]
101	x									x				x		-	22477 [2539, 73165]	31 [-1047, 1131]
102	x									x					x	-	12735 [1288, 41564]	0 [-837, 699]
103		x								x			x			-	32027 [1501, 140503]	8 [-3398, 3346]
104		x								x				x		-	34596 [2479, 126474]	41 [-1094, 1282]

	Antibiotics*	Antibiotics**	OCV	POUWT	Clusters 100m	Clusters 70m	Clusters 45m	Clusters 30m	Clusters 15m	Random	Entire population	By district	Early	Peak	Late	Clusters targeted	People targeted	Cases averted
105		x								x					x	-	14760 [1248, 51802]	6 [-748, 629]
106			x							x			x			-	5044 [1083, 13489]	152 [-3754, 3730]
107			x							x				x		-	12862 [1915, 43188]	99 [-845, 1230]
108			x							x					x	-	11064 [1291, 33596]	17 [-660, 821]
109				x						x			x			-	14263 [1200, 57750]	47 [-3417, 3558]
110				x						x				x		-	19632 [2590, 64664]	38 [-898, 1192]
111				x						x					x	-	12249 [1267, 37495]	2 [-650, 683]
