# Supplementary material for: The potential impact of case-area targeted interventions in response to cholera outbreaks: A modeling study
Source: PLoS Med. 2018 Feb 27;15(2):e1002509. doi: 10.1371/journal.pmed.1002509 (PMC5828347; doi:10.1371/journal.pmed.1002509)
Supplement: S1 Text — Description of the data used, the implementation of the epidemiological model, its calibration, and the implementation of intervention strategies. (PDF) [file pmed.1002509.s025.pdf]

S1 Text

The potential impact of case-area targeted interventions in response to cholera outbreaks: A modeling study

Quantification of spatiotemporal clustering

We use the τ statistic introduced by Lessler et al. (2016), which has been shown to be a good measure of global spatial clustering for epidemiological applications (Salje et al. 2012, Grabowski et al. 2014, Salje et al. 2016, 2017), to quantify spatiotemporal clustering of cholera cases. Here, we consider two cases to be potentially transmission related (e.g. to potentially share a recent common ancestor) if they occurred within a time interval of 0 to 4 days from each other (using upper bounds between 2 and 6 days gives similar results, S10 Fig). τ is then defined as the relative risk that a person in a given distance range $[d_1, d_2]$ from a disease case also becomes a case that is potentially transmission related (i.e. to become infected within a time interval of 0 to 4 days), compared to the risk of any person in the population becoming a potentially transmission related case. $\hat{\tau}(d_1, d_2)$ can be computed by dividing the estimated odds ratio $\hat{\theta}(d_1, d_2)$ of the number of potentially transmission related cases against non-transmission related cases within $[d_1, d_2]$ by the same odds ratio computed for the whole domain $\theta(0, \infty)$ (Lessler et al. 2016):

$$\hat{\theta}(d_1, d_2) = \frac{\sum_i \sum_j \mathbf{I_1}(i, j)}{\sum_i \sum_j \mathbf{I_2}(i, j)} \quad (1)$$

$$\hat{\tau}(d_1, d_2) = \frac{\hat{\theta}(d_1, d_2)}{\hat{\theta}(0, \infty)}, \quad (2)$$

where $\mathbf{I_1}(i, j)$ denotes an indicator function which is equal to one if cases i and j are within the distance range $[d_1, d_2]$ from each other and within the time interval of 0 to 4 days, and zero otherwise. $\mathbf{I_2}(i, j)$ is an indicator function which is equal to one if cases i and j are within the distance range $[d_1, d_2]$ from each other but not transmission related (i.e. with the time interval between the cases longer than 4 days), and zero otherwise.

Epidemiological model

We employ a spatially explicit, individual-based stochastic epidemiological model (see e.g. Keeling & Rohani (2008)) with a timestep Δt of one day. The N individuals in the model space are assigned random positions according to the population distribution (Section Spatial setup and population distribution) and can either be susceptible (S), exposed (E), infected (I) or recovered (R). A power-law-distribution shaped isotropic kernel (K_1) originating from the position of every infected and decreasing with distance d accounts for the local transmission of the disease, whereas a constant, distance-invariant kernel (K_2) accounts for long-distance transmission:

$$K_1(d) = \frac{a-2}{2\pi\left(d_0^{-a+2} - d_{\max}^{-a+2}\right)} d^{-a} \tag{3}$$

$$K_2 = \frac{1}{\pi\left(d_{\max}^2 - d_0^2\right)} \tag{4}$$

$$K(d) = (1-c)K_1(d) + cK_2. \tag{5}$$

The shape of K_1 is determined by the parameter a defined to be greater than 2. The minimal distance d_0 is fixed to 15 m, half the grid size of our case study, and the upper distance limit $d_{\max}$ is set to 30 km, the approximate diameter of the study domain (Section Spatial setup and population distribution). The constant c determines the probability of random, long-distance transmission events with respect to local transmission events. In the main analysis, this constant is set to 0. To evaluate the influence of long-distance transmission events, an additional analysis with $c = 0.05$ has been performed (Section The role of long distance transmission).

The force of infection F_i affecting a susceptible i depends on his position in the model space relative to infected individuals and can be computed by taking the sum of the kernels originating from all infected evaluated at the position of i and multiplying it with individual exposure parameters, as well as a term accounting for rainfall:

$$F_i(t) = \beta_i\left(1 + \lambda r(t)\right) \sum_{\substack{j=1 \\ j \neq i}}^{N} \mathbf{I_I}(j)\theta_j K(d_{i,j}), \tag{6}$$

where β_i is an individual exposure parameter, $\mathbf{I_I}(j)$ is an indicator function whose value is equal to 1 if individual j is infected and 0 otherwise and θ_j is fixed to 1 in the main analysis. λ is a parameter that multiplies daily precipitation $r(t)$.

Exposure events are assumed to follow a Poisson process with rate $F_i(t)$. The resulting probability of susceptible i being exposed during Δt is given in (7). A fraction σ_i of infections is symptomatic. Asymptomatic individuals recover immediately after exposure, their contribution to the environmental bacterial concentration is assumed to be negligible (Nelson et al. 2009). Symptomatic individuals stay in the exposed state for a time t_E and in the infected state for a time t_I, which are drawn from gamma distributions according to (8) (Azman et al. 2013) and (9) (Kaper et al. 1995).

$$P(S_i \to E_i) = \left(1 - e^{F_i(t)\Delta t}\right) \tag{7}$$
$$t_E \sim \Gamma(2, 0.5) \tag{8}$$
$$t_I \sim \Gamma(10, 0.5)\,. \tag{9}$$

A timestep t of a model simulation thus consists of the following steps:

1. update states of individuals from E to I or from I to R if their t_E or t_I is reached
2. compute $F_i(t)$ (6) at the position of every susceptible i
3. use (7) to determine susceptibles who get exposed by drawing a uniform random number p_i for each of them: if $p_i < P(S_i \to E_i)$ the individual gets exposed
4. for every exposed individual, draw a random number q_i to determine if she/he gets symptomatic or asymptomatic: if $q_i < \sigma_i$, the infection is symptomatic and the individual goes to the infected class I, otherwise she/he recovers and goes to class R.

To accelerate the model run for large populations and vast areas, the model space is a discrete grid (Section Spatial setup and population distribution) and the convolution between the kernel and the distance matrix is done using the Fast Fourier Transform.

Spatial setup and population distribution

The domain of our model is the city of N'Djamena, Chad, subdivided into regular grid cells (30 m by 30 m). The remotely sensed built-up density (S2 Fig) (Esch et al. 2011, 2017) was used as a proxy for the small scale spatial population density.

Every inhabitant (993 500 individuals) was randomly assigned to a grid cell with a probability proportional to the estimated average built-up density of the cell. The euclidean distances between the centers of the two cells between persons who live in distinct grid cells were used to compute the value of the infection kernel (4). A distance of 10 m was assumed between two persons living in the same grid cell.

Rainfall data

Daily precipitation (S11 Fig) was obtained from the NASA TRMM Version 7 Daily Precipitation Estimates (Huffman et al. 2010)*.

*http://iridl.ldeo.columbia.edu/SOURCES/.NASA/.GES-DAAC/.TRMM_L3/.TRMM_3B42/.v7/.daily/.precipitation/X/15.0/15.25/RANGEEDGES/Y/12/12.25/RANGEEDGES/T/(01%20Apr%202011)(01%20May%202012)RANGEEDGES/, accessed on June 29, 2016

Initial conditions

An initial number of 25 randomly chosen individuals in the model space are set to be symptomatically infected. They are assumed to be infectious for a period t_I according to (9), starting from a point in time between 4 and 1 days before the start of the model. The remaining part of the population is assumed to be susceptible.

Calibration

In the absence of treatments, parameter values are assumed to be the same for all individuals (i.e. $\beta_i = \beta$ and $\sigma_i = \sigma$). Four free parameters of our model (σ, β, a and λ) were calibrated to match the characteristics of the real epidemic in N'Djamena.

We employed a Python implementation of the Approximate Bayesian Computation Population Monte Carlo (ABC-PMC) algorithm (Beaumont et al. 2009, Akeret et al. 2015). Two summary statistics were used: the sum of squared residuals (10) on the reported number of cases (Fig. 2A) and on the τ statistic over 3 different distance ranges (15 m to 45 m, 45 m to 105 m and 105 m to 225 m) and with a time range of 2 to 4 days (Fig. 2B). The distance ranges have been chosen to fit the spatial discretization of the model domain (Section Spatial setup and population distribution). The calibration implied running the model with a high number of particles (i.e. parameter sets), which were accepted if the resulting summary statistics were under certain thresholds (130 000 and 140 during the first calibration step). A step was completed after 512 particles had been accepted, the acceptance rate varied between 15% initially and 2% during the last step. After each calibration step the thresholds were adapted to the 85th percentile of the summary statistic values taken by the particles of the previous step (Akeret et al. 2015). Particles for a new calibration step were drawn from the accepted particles of the previous step and perturbed using a multivariate normal kernel with optimal local covariance matrix (Filippi et al. 2013, Akeret et al. 2015). The calibration was stopped after 15 steps because the acceptance rate had fallen below 2% and the posterior had reached a stable state (Akeret et al. 2015). The final thresholds for the two summary statistics were 41 923 and 1.94 respectively. Samples from the posterior parameter distribution are shown in S3 Fig.

$$ssr(x, \hat{x}) = \sum_{i=1}^{N} (x_i - \hat{x}_i)^2 \tag{10}$$

Simulation

To run a simulation, a parameter set is drawn from the posterior distribution and used to run the model. All results and figures presented are derived from a set of 1000 simulation runs. Note that the outcome of two model runs with identical parameter sets may differ because of stochastic processes. Simulations are run either until the

end of the epidemic (no infected or exposed present in the model), or up to a maximal duration of 1 year.

Implementation of interventions

We consider three different types of preventive interventions and combinations thereof: the administration of a single dose of antibiotics, household scale water, sanitation and hygiene (POUWT, stands for point-of-use water treatment) measures and the administration of a single dose of oral cholera vaccine (OCV). The implementation of the effects of those interventions are described below. Values and references are summarized in Table 1 in the main text and S14 Fig. In addition to these types of interventions and their possible combinations, we also consider different strategies to select people to benefit from the interventions, and different points in time when the application of interventions start. S1 Table summarizes all interventions tested for this study along with their outcome.

Antibiotics

We consider the joint effect of two mechanisms of protection against cholera by antibiotics: a reduced probability of acquiring infection (Reveiz et al. 2011) and a lower probability to get symptoms if exposed (Echevarria et al. 1995). As studies have not yet quantified the combined effect (Reveiz et al. 2011), we follow Lewnard et al. (2016) and estimate the joint effect by multiplying individual effects. Finally, the joint effect is translated into a relative risk of symptomatic infection of 0.045 [95% CI 0.001 to 0.296] multiplied with parameter σ_i.

Antibiotics have also been found to reduce the duration of bacterial shedding (Leibovici-Weissman et al. 2014, Lewnard et al. 2016), which we model through a reduction of t_I by -2.74 [95% CI -3.07 to -2.40] days.

We estimate that the beneficial effects of antibiotics last for 2 days, as the drug concentration in stools has been shown to be sufficient to eliminate *Vibrio Cholerae* during this period of time after the administration of a single dose of Azithromycin in a clinical trial (Khan et al. 2002).

OCV

The administration of a single dose of OCV affects the chances of an exposed individual i to get symptomatic (e.g. to get severe cholera) by multiplying parameter σ_i with the relative risk of symptomatic infection 0.37 [95% CI 0.18 to 0.76], which corresponds to one minus the vaccine efficacy reported by Qadri et al. (2016) for severe cholera episodes. As beneficial effects of the vaccine have only been confirmed after a lag of 7 days (Qadri et al. 2016), we assume that the vaccine takes effect only 1 week after administration.

POUWT

WaSH and in particular POUWT interventions reduce the probability of individuals to get exposed to an infectious dose of *Vibrio Cholerae*. In the model, this is achieved by multiplying the exposure parameter β_i for targeted individuals i with the relative risk of exposure 0.74 [95% CI 0.65 to 0.85], reported by Fewtrell et al. (2005) for household scale water quality interventions in (peri-)urban settings.

Combined interventions

We also consider combinations between the three main type of interventions (S1 Table). This is achieved by simultaneously applying the estimated effects of several interventions to the targeted population.

Duration of intervention effects

Whereas the duration of the effects of antibiotics is short (2 days, see Antibiotics), we consider that protection from OCV and POUWT lasts (at least) until the end of the current epidemic. This implies that people who benefited from OCV or POUWT interventions once don't need to be treated again. In the case of antibiotics, as the effect vanishes rapidly, we consider two scenarios, one in which every person can get antibiotics only once during the epidemic (main text), and one in which a single person can be allocated antibiotics several times, with a minimal interval of 2 weeks (Section Allocation of several doses of antibiotics per person).

Uncertainty of intervention effects

To propagate uncertainty regarding intervention effects we use the distributions shown in S14 Fig, which have been obtained by fitting normal or log-normal distributions to the reported 95% confidence intervals shown in Table 1 in the main text or directly from the cited references. During the simulation, a different set of reduction parameters is drawn for every person treated.

Intervention timing

We consider three different scenarios as to when interventions start:

early (day 50) interventions are launched during the flat phase early in the epidemic,

peak (day 130) around the paeak of the epidemic,

late (day 180) after the epidemic peak, during the recession phase.

During simulation, we assume that intervention scenarios are only started when at least 10 new cases were reported during the week before the start date.

Allocation strategies

Case-area targeted allocation (CATIs)

In addition to the different kinds of interventions we consider different intervention strategies. The first strategy takes advantage of the clustering of cases. It consists in targeting people with an increased risk of getting exposed to *V. cholerae* because they are living within a given distance (in time and space) to a known case. Every time a case gets reported (e.g. when a person in the model changes from the exposed (E) to the symptomatically infected state (I)), people who live within a distance of 100 m of the reported case's home are targeted by the intervention. In N'Djamena a cluster of this radius typically consists of 100 to 500 people (S12 Fig). We also evaluate the effect of reducing this radius to 70 m, 45 m, 30 m and 15 m, measured within the gridded model space (S13 Fig). To account for the fact that an intervention team visiting the target area will not be able to reach all inhabitants because they might be absent or might not agree to receive preventive treatment or not comply with POUWT measures, we consider that a random sample of 70% of the people who live within the designated area can be effectively reached. In addition, we account for the delay between the reporting of the initial case and the deployment of an intervention team to the corresponding cluster by drawing it from a distribution given in S15 Fig, considering that all clusters can be targeted within 7 days counting from the reporting of the initial case (day 0), with the mode on day 2. We assume that interventions continue until the end of the epidemic (i.e. no more reported cases) or the maximal duration of the simulation (Section Simulation).

Mass campaign targeting the same number of people

To estimate the comparative advantage of CATIs, we also simulated a second strategy, where we allocated the interventions at the same starting time as we would have in the targeted interventions and to the same total number of people for each simulation run, though randomly in space. We assume that all interventions can be administered within 14 days from the start date.

Mass campaign targeting 70% of the population

To estimate the effect of randomly allocated interventions to a high number of people (i.e. mass interventions), we simulated a strategy where we allocated the interventions at the same time as we would have in the targeted interventions, but randomly to 70% of all people living in the city. We consider that all interventions can be administered within 14 days from the start date.

District-wise mass campaign

An alternative strategy to a mass intervention campaign targeting the entire city is to target only certain districts, selected by criteria such as number of reported cases or indicators of vulnerability to cholera, at the advantage to simplify the logistics and

reduce the required number of doses or intervention teams needed. To explore the potential advantage of such a strategy we implemented an additional scenario where, for each simulated epidemic, interventions are allocated to the population of the 3 districts (out of a total of 10, S1 Fig) with the highest attack rate at the time when interventions start, assuming that 70% of the districts population can be effectively reached.

Additional results

Allocation of antibiotics to household members and closest neighbors in combination with allocation of OCV in larger radii

Despite the high efficiency of case-area targeted allocation of antibiotics, especially at smaller radii or in combination with OCV, the administration of such drugs to a high number of people unavoidably raises concerns about the development of antimicrobial resistances. As an alternative, we consider scenarios where antibiotics are administered only to household members and closest neighbors of cholera patients, combined with the allocation of OCV in a larger radius, limiting the number people getting antibiotics and thus the potential risk of widespread antimicrobial resistance. We evaluate the administration of antibiotics within the same model cell (i.e. a radius of 15 m) as the reported case and the simultaneous allocation of OCV within a larger radius, varying between 30 m and 100 m, thus combining the advantage of rapid onset of protection by antibiotics at short distances with the long lasting protective effect of OCV. More cases are averted for this strategy than for for OCV alone, particularly at low and intermediate radii (S8 Fig and S1 Table). Adding the administration of antibiotics within the first 15 m around cases to an OCV campaign at a radius of 45 m starting early, the median number of averted cases increases from 469 (95% prediction interval (PI) -2730 to 4531) to 632 (95% PI -2455 to 4276). The effect is less pronounced (increase of averted cases from 2986 (95% PI 0 to 11090) to 3036 (95% PI -8 to 10748) in a radius of 70 m when starting early) at larger radii because OCV alone already leads to high numbers of averted cases. With respect to the combined administration of OCV and antibiotics at a radius of 100 m starting early, reducing the radius for antibiotics to 15 m only leads to a minimal decrease from 3425 (95% PI 38 to 12203) to 3315 (95% PI 14 to 11893) in the number of averted cases.

Allocation of several doses of antibiotics per person

Restricting the number of doses of antibiotics a person can receive to one during the whole study period, combined with the short duration of protection offered, limits the efficiency of CATIs with antibiotics. In an alternative scenario, we assume that a person can get several doses of antibiotics, with a minimal interval of 2 weeks, if she/he lives within the intervention radius of several cases reported at different times. By allowing for repeated targeting of a single person, we see an improved efficacy of case-area targeted allocation of antibiotics (S1 Table), even at high radii, such as

100 m. Starting around the epidemic peak, the averted cases increase from 532 (95% PI -371 to 2220) to 1034 (95% PI 28 to 3698), at the cost of a increase in the number of people to target from 22477 (95% PI 2539 to 73165) to 34596 (95% PI 2479 to 126474) (i.e. doses to administrate (S9 Fig)).

The role of long distance transmission

In this study, we aimed at reproducing simulated epidemics that mimic the course and the global spatial clustering of the N'Djamena epidemic. We thus chose to fit our model to the epidemic curve and the τ statistic simultaneously. In addition, our model is based on the assumption that the probability of a transmission event from an infected to a susceptible individual steadily decreases with distance (Section Epidemiological model). Long-distance transmission events, which cause the spread of the disease from one neighborhood to another by the means of travelers and commuters, are thus unlikely to occur. In this set-up, our model is unable to fit the global spatial structure of the epidemic (e.g. the spatial distribution among different clusters of cases and among different neighborhoods), in particular because of the lack of a mechanism for long-distance transmission and the lack of a calibration criterion quantifying the mentioned global structure.

We thus performed a sensitivity analysis investigating the influence of adding long-distance transmission events to the model by the means of setting parameter c to 0.05 (Section Epidemiological model), meaning that 5% of individuals to be infected are chosen regardless of their position within the city among all susceptible people.

The calibration was stopped after the acceptance rate had fallen below 2%. Calibration results (S16 Fig and S17 Fig) reveal that the model was able to reproduce the key characteristics of the epidemic, i.e. the evolution of new cases over time and the spatiotemporal clustering of cases (τ), even if the quality of the fit was lower with respect to the results presented in the main paper. This, together with the slower convergence during calibration, can be explained by the fact that the additional long-distance transmission added increases the difficulty to fit the clustering statistic τ because it introduces an additional constraint and leads to a general increase of the scattering of cases (and clusters thereof) in space.

A comparison of the results of the main CATIs (S18 Fig and S19 Fig) with the two values of c reveals that the general rank order of types of intervention with respect to numbers of averted cases is nearly unchanged. The lower medians of averted cases with OCV and POUWT with long-distance transmission can be explained by the fact that the long-distance transmission introduces more infections into areas previously not targeted by interventions, and the higher spread of the distributions are due to the above-mentioned higher variability in the simulated epidemic curves due to the quality of the fit (S16 Fig). The increased number of averted cases when administering antibiotics results from a higher proportion of people in targeted areas who have not previously received antibiotics because the clusters of cases are generally further apart. This effect is especially important due to the short duration of protection offered and the limitation of one dose per person throughout the study period (Section Allocation of several doses of antibiotics per person). Similarly, the number of persons targeted

is generally higher with all three types of interventions because the targeted clusters have less overlap. The average number of clusters targeted is lower for Antibiotics and POUWT when comparing to the simulations without long-distance transmission because epidemics are averted more efficiently.

Potential impact of asymptomatic shedding

There is an ongoing debate about the importance of the role of asymptomatic shedding in sustaining cholera outbreaks and how to translate the underlying processes into mechanistic models (Grad et al. 2012, Fung 2014). Asymptomatic patients are known to shed *Vibrio cholerae*, however, the rate as well as the duration of shedding are significantly lower than for symptomatically infected (Nelson et al. 2009, Cash et al. 1974). Thus, in our primary analysis we assumed that asymptomatics do not significantly contribute to the force of infection. To investigate the potential impact of asymptomatic shedding on the intervention results we performed an extensive sensitivity analysis, in which we assumed that each asymptomatically infected contributes 10% as much to the force of infection as a symptomatic at the same geographical position would have by setting parameter θ_j to 0.1 for those individuals (Section Epidemiological model). The shedding duration was fixed to 1 day for asymptomatic individuals (Nelson et al. 2009). Parameters were recalibrated except for σ, which was kept fixed to its mean value resulting from the main analysis to facilitate convergence. The calibration was stopped after the acceptance rate had fallen below 2%. Calibration results reveal that the model was able to reproduce the key characteristics of the epidemic, i.e. the evolution of new cases over time and the spatiotemporal clustering of cases (τ), even if the quality of the fit was lower with respect to the results presented in the main paper.

A comparison of the results of CATIs in a 100 m radius with all three intervention types with and without asymptomatic shedding reveals that even if the impact of CATIs are lower the general rank order of interventions did not change (S23 Fig). A notable difference is the lower reduction of epidemic time for early interventions, possibly due to asymptomatic shedders causing the simulated outbreaks to revamp. Another result from these analyses is that antibiotics become significantly less effective to the point of having close to no effect, which can be attributed to the asymptomatics shedding duration (one day) which is not further decreased by the intervention. Using CATIs with OCV in a 100 m radius around peak time in a model with asymptomatic shedders 21% (IQR 15% to 31%) of cases were averted and epidemic durations reduced by 21% (IQR 12% to 30%), against 43% (IQR 35% to 49%) and 35% (IQR 26% to 66%) respectively in our primary analysis.

The effect of imperfect CATI targeting

A set of key assumption of this study is that (1) all symptomatic cases get reported and (2) 70% of the neighbors of all reported cases get targeted by CATIs. We performed a sensitivity analysis to explore the impact of CATIs when relaxing those assumptions by lowering the fraction of symptomatic cases which result in a 100 m ring being targeted by OCV in different scenarii to 80%, 60%, 40% or 5%.

Even when only a fraction of the symptomatic cases are assumed to trigger a CATI, they remain similarly effective as shown in our primary analyses (S20 Fig and S21 Fig). Targeting 100 m rings around 60% of all symptomatic cases with OCV at peak time lead to 1711 (IQR 780 to 2830) averted cases, compared to 1784 (IQR 825 to 2987) when targeting around all symptomatic cases. The number of targeted persons increased to 13092 (IQR 6848 to 21421) and the number of targeted clusters dropped to 386 (IQR 190 to 695). The epidemic duration was reduced by 89 (IQR 57 to 138) days, compared to 97 (IQR 62 to 140) when targeting around all symptomatics. As expected, when targeting CATIs within 100 m around a low proportion of symptomatic cases (5%), we saw a significant reduction in intervention effect. When CATIs started around the epidemic peak the averted cases decreased to 150 (IQR 27 to 442) and the reduction of epidemic time to 32 (IQR 5 to 69) days, which is lower than the reductions achieved by district targeted mass campaigns.

The outcome of CATIs is thus robust to a decrease in the number of targeted rings, which can be attributed to the fact that many rings overlap. This could reveal crucial in practical applications because of imperfect surveillance systems and/or logistical constraints may limit the number of rings that can be targeted, even if full coverage has been shown feasible (Parker et al. 2017).

Reduction of the symptomatic fraction through POUWT

Our parametrization of POUWT interventions relies on the assumption that the mechanism which leads to protection through POUWT is a reduced concentration of *Vibrio choleræ* in drinking water, which in term reduces the force of infection acting upon individuals and thus the probability of getting infected. This mechanism, in which the likelihood of infection depends on the ingested dose, has been observed in experimental human challenge studies (Hornick et al. 1971). However, as noted by Grad et al. (2012) and Fung (2014), not only the likelihood of infection but also the likelihood of an infection being symptomatic is dose-dependent (Hornick et al. 1971), suggesting the reduction of the symptomatic fraction as another mechanism of action of POUWT.

To determine if the effect of CATIs using POUWT is dependent on the mechanism assumed, we re-run POUWT simulations using this second mechanism (i.e. assuming a relative risk of symptomatic infection of 0.74 (95% CI 0.65, 0.85) for targeted individuals instead of a relative risk of exposure). Results show that the benefits of CATIs using POUWT in a 100 m radius around cases are higher when considering this second mechanism, but still lower than effects with CATIs using OCV (S22 Fig). When intervening at peak time the number of averted cases was 1649 (IQR 742 to 2656) and the reduction in epidemic days 67 (IQR 36 to 107) against 833 (IQR 355 to 1530) cases and 39 (IQR 8 to 74) days in our primary analysis.

Previous immunity in CATIs

In CATIs, contacts of cases are more likely than randomly-selected individuals to have been previously infected (symptomatically or asymptomatically) and thus to benefit from acquired immunity. This is why a higher proportion of intervention resources

may go to previously immune than with other modes of intervention allocation. Of note, as shown in our primary analysis, CATIs are more ressource efficient than mass interventions overall because they target people most at risk to get cholera.

To give a sense for how much wastage there could be through CATIs compared to perfect targeting around households of susceptible individuals, we performed 1000 new simulations where at each of the three intervention starting times, we chose 100 households of infected individuals and 100 households of susceptible individuals. Within 100 m around each household we calculated the number of people immune to cholera and then combined results to have the relative risk of wasting an intervention comparing interventions around cases to those around susceptible people. In this extreme example, we found that the risk of targeting a person who may benefit from interventions (i.e., not immune) was reduced 1.7-fold (IQR 1.6 to 1.8) when interventions were targeted within 100 m around households of cases compared to interventions targeted around susceptible households at the start of the epidemic. This reduction increased later in the epidemic, with a 2.3-fold (IQR 2.1 to 2.5) reduction with CATIs starting around peak and a 2.2-fold (IQR 2.0 to 2.3) reduction with CATIs starting at the end of epidemics.

References

Akeret, J., Refregier, A., Amara, A., Seehars, S. & Hasner, C. (2015), 'Approximate Bayesian computation for forward modeling in cosmology', *Journal of Cosmology and Astroparticle Physics* **2015**(08), 043.

Azman, A. S., Rudolph, K. E., Cummings, D. A. T. & Lessler, J. (2013), 'The incubation period of cholera: A systematic review', *Journal of Infection* **66**(5), 432–438.

Beaumont, M. A., Cornuet, J.-M., Marin, J.-M. & Robert, C. P. (2009), 'Adaptive approximate Bayesian computation', *Biometrika* p. asp052.

Cash, R. A., Music, S. I., Libonati, J. P., Snyder, M. J., Wenzel, R. P. & Hornick, R. B. (1974), 'Response of Man to Infection with Vibrio cholerae. I. Clinical, Serologic, and Bacteriologic Responses to a Known Inoculum', *The Journal of Infectious Diseases* **129**(1), 45–52.

Echevarria, J., Seas, C., Carrillo, C., Mostorino, R., Ruiz, R. & Gotuzzo, E. (1995), 'Efficacy and Tolerability of Ciprofloxacin Prophylaxis in Adult Household Contacts of Patients with Cholera', *Clinical Infectious Diseases* **20**(6), 1480–1484.

Esch, T., Heldens, W., Hirner, A., Keil, M., Marconcini, M., Roth, A., Zeidler, J., Dech, S. & Strano, E. (2017), 'Breaking new ground in mapping human settlements from space – The Global Urban Footprint', *ISPRS Journal of Photogrammetry and Remote Sensing* **134**, 30–42.
URL: *http://www.sciencedirect.com/science/article/pii/S0924271617301880*

Esch, T., Schenk, A., Ullmann, T., Thiel, M., Roth, A. & Dech, S. (2011), 'Characterization of Land Cover Types in TerraSAR-X Images by Combined Analysis of Speckle Statistics and Intensity Information', *IEEE Transactions on Geoscience and Remote Sensing* **49**(6), 1911–1925.

Fewtrell, L., Kaufmann, R. B., Kay, D., Enanoria, W., Haller, L. & Colford Jr, J. M. (2005), 'Water, sanitation, and hygiene interventions to reduce diarrhoea in less developed countries: A systematic review and meta-analysis', *The Lancet Infectious Diseases* **5**(1), 42–52.

Filippi, S., Barnes, C. P., Cornebise, J. & Stumpf, M. P. (2013), 'On optimality of kernels for approximate Bayesian computation using sequential Monte Carlo', *Statistical Applications in Genetics and Molecular Biology* **12**(1), 87–107.

Fung, I. C.-H. (2014), 'Cholera transmission dynamic models for public health practitioners', *Emerging Themes in Epidemiology* **11**, 1.

Grabowski, M. K., Lessler, J., Redd, A. D., Kagaayi, J., Laeyendecker, O., Ndyanabo, A., Nelson, M. I., Cummings, D. A. T., Bwanika, J. B., Mueller, A. C., Reynolds, S. J., Munshaw, S., Ray, S. C., Lutalo, T., Manucci, J., Tobian, A. A. R., Chang, L. W., Beyrer, C., Jennings, J. M., Nalugoda, F., Serwadda, D., Wawer, M. J., Quinn, T. C., Gray, R. H. & Program, t. R. H. S. (2014), 'The Role of Viral Introductions in Sustaining Community-Based HIV Epidemics in Rural Uganda: Evidence from Spatial Clustering, Phylogenetics, and Egocentric Transmission Models', *PLOS Medicine* **11**(3), e1001610.

Grad, Y. H., Miller, J. C. & Lipsitch, M. (2012), 'Cholera Modeling: Challenges to Quantitative Analysis and Predicting the Impact of Interventions', *Epidemiology* **23**(4), 523–530. bibtex: grad_2012.

Hornick, R. B., Music, S. I., Wenzel, R., Cash, R., Libonati, J. P., Snyder, M. J. & Woodward, T. E. (1971), 'The Broad Street pump revisited: response of volunteers to ingested cholera vibrios.', *Bulletin of the New York Academy of Medicine* **47**(10), 1181–1191.

Huffman, G. J., Adler, R. F., Bolvin, D. T. & Nelkin, E. J. (2010), The TRMM multi-satellite precipitation analysis (TMPA), *in* M. Gebremichael & F. Hossain, eds, 'Satellite Rainfall Applications for Surface Hydrology', Springer Netherlands, pp. 3–22.

Kaper, J. B., Morris, J. G. & Levine, M. M. (1995), 'Cholera', *Clinical Microbiology Reviews* **8**(1), 48–86.

Keeling, M. J. & Rohani, P. (2008), *Modeling Infectious Diseases in Humans and Animals*, Princeton University Press.

Khan, W. A., Saha, D., Rahman, A., Salam, M. A., Bogaerts, J. & Bennish, M. L. (2002), 'Comparison of single-dose azithromycin and 12-dose, 3-day erythromycin for

childhood cholera: A randomised, double-blind trial', *The Lancet* **360**(9347), 1722–1727.

Leibovici-Weissman, Y., Neuberger, A., Bitterman, R., Sinclair, D., Salam, M. A. & Paul, M. (2014), Antimicrobial drugs for treating cholera, *in* 'Cochrane Database of Systematic Reviews', John Wiley & Sons, Ltd.

Lessler, J., Salje, H., Grabowski, M. K. & Cummings, D. A. T. (2016), 'Measuring Spatial Dependence for Infectious Disease Epidemiology', *PLOS ONE* **11**(5), e0155249.

Lewnard, J. A., Antillón, M., Gonsalves, G., Miller, A. M., Ko, A. I. & Pitzer, V. E. (2016), 'Strategies to Prevent Cholera Introduction during International Personnel Deployments: A Computational Modeling Analysis Based on the 2010 Haiti Outbreak', *PLOS Medicine* **13**(1), e1001947.

Nelson, E. J., Harris, J. B., Glenn Morris, J., Calderwood, S. B. & Camilli, A. (2009), 'Cholera transmission: The host, pathogen and bacteriophage dynamic', *Nature Reviews Microbiology* **7**(10), 693–702.

Parker, L. A., Rumunu, J., Jamet, C., Kenyi, Y., Lino, R. L., Wamala, J. F., Mpairwe, A. M., Muller, V., Llosa, A. E., Uzzeni, F., Luquero, F. J., Ciglenecki, I. & Azman, A. S. (2017), 'Neighborhood-targeted and case-triggered use of a single dose of oral cholera vaccine in an urban setting: Feasibility and vaccine coverage', *PLOS Neglected Tropical Diseases* **11**(6), e0005652.

Qadri, F., Wierzba, T. F., Ali, M., Chowdhury, F., Khan, A. I., Saha, A., Khan, I. A., Asaduzzaman, M., Akter, A., Khan, A., Begum, Y. A., Bhuiyan, T. R., Khanam, F., Chowdhury, M. I., Islam, T., Chowdhury, A. I., Rahman, A., Siddique, S. A., You, Y. A., Kim, D. R., Siddik, A. U., Saha, N. C., Kabir, A., Cravioto, A., Desai, S. N., Singh, A. P. & Clemens, J. D. (2016), 'Efficacy of a Single-Dose, Inactivated Oral Cholera Vaccine in Bangladesh', *New England Journal of Medicine* **374**(18), 1723–1732.

Reveiz, L., Chapman, E., Ramon-Pardo, P., Koehlmoos, T. P., Cuervo, L. G., Aldighieri, S. & Chambliss, A. (2011), 'Chemoprophylaxis in Contacts of Patients with Cholera: Systematic Review and Meta-Analysis', *PLOS ONE* **6**(11), e27060.

Salje, H., Cauchemez, S., Alera, M. T., Rodriguez-Barraquer, I., Thaisomboonsuk, B., Srikiatkhachorn, A., Lago, C. B., Villa, D., Klungthong, C., Tac-An, I. A., Fernandez, S., Velasco, J. M., Roque, V. G., Nisalak, A., Macareo, L. R., Levy, J. W., Cummings, D. & Yoon, I.-K. (2016), 'Reconstruction of 60 Years of Chikungunya Epidemiology in the Philippines Demonstrates Episodic and Focal Transmission', *Journal of Infectious Diseases* **213**(4), 604–610.

Salje, H., Lessler, J., Berry, I. M., Melendrez, M. C., Endy, T., Kalayanarooj, S., A-Nuegoonpipat, A., Chanama, S., Sangkijporn, S., Klungthong, C., Thaisomboonsuk, B., Nisalak, A., Gibbons, R. V., Iamsirithaworn, S., Macareo, L. R., Yoon, I.-K., Sangarsang, A., Jarman, R. G. & Cummings, D. A. T. (2017), 'Dengue diversity

across spatial and temporal scales: Local structure and the effect of host population size', *Science* **355**(6331), 1302–1306.

Salje, H., Lessler, J., Endy, T. P., Curriero, F. C., Gibbons, R. V., Nisalak, A., Nimmannitya, S., Kalayanarooj, S., Jarman, R. G., Thomas, S. J., Burke, D. S. & Cummings, D. A. T. (2012), 'Revealing the microscale spatial signature of dengue transmission and immunity in an urban population', *Proceedings of the National Academy of Sciences of the United States Of America* **109**(24), 9535–9538.
