# Supplementary material for: The potential impact of case-area targeted interventions in response to cholera outbreaks: A modeling study
Source: PLoS Med. 2018 Feb 27;15(2):e1002509. doi: 10.1371/journal.pmed.1002509 (PMC5828347; doi:10.1371/journal.pmed.1002509)
Supplement: S1 Alternative Language Abstract — (PDF) [file pmed.1002509.s026.pdf]

The potential impact of case-area targeted interventions in response to cholera outbreaks: A modeling study

Résumé

Contexte

La stratégie CATI (case-area targeted interventions) qui allie la prévention du choléra et des interventions réactives ciblées dans le voisinage des cas de Choléra, comprend plusieurs activités dont le traitement de l'eau, l'hygiène et l'assainissement, le vaccin oral et le traitement antibiotique prophylactique. Cette stratégie permettrait d'éviter d'une manière efficiente l'augmentation du nombre de cas et de décès en cours d'épidémie. Des activités de riposte dans le voisinage des cas de choléra ont été menées dans de récentes épidémies, mais peu de données empiriques sont disponibles pour démontrer leur efficacité et efficience, ainsi que pour optimiser leur ciblage. Nous voulons ici apporter des éléments pratiques de la mise en œuvre de celles-ci, en explorant les déterminants de l'impact des interventions, dont la zone de ciblage et le timing, lors d'épidémies de choléra simulées, calibrées sur les données d'une épidémie de choléra en milieu urbain en Afrique.

Méthode et résultats

Nous avons développé un modèle de micro-simulation qui a été calibré pour reproduire à la fois la courbe épidémique totale ainsi que la répartition spatiale des cas de l'épidémie survenue en 2011 à N'Djaména, Tchad, avec 4352 cas rapportés en 232 jours. L'implémentation des CATIs dans les jours suivant l'admission des cas aux centres de soins a été simulée avec différentes combinaisons de réponses : prophylaxie antimicrobienne, vaccin oral, et traitement d'eau au point d'utilisation. Ces approches ont été simulées à des moments différents au cours de l'épidémie, et avec différents rayons d'action autour des ménages des cas déclarés. Nos conclusions suggèrent que les CATIs raccourcissent la durée de l'épidémie, et sont plus efficientes que des campagnes de masse qui ciblent des populations à plus grande échelle. L'analyse suggère que l'application des CATIs utilisant la vaccination est l'intervention singulière la plus efficace, suivie par le traitement d'eau au point d'utilisation, et la distribution d'antibiotiques. La vaccination tôt dans l'épidémie, ciblée dans un rayon de 100 m autour des ménages des cas déclarés réduit la durée des épidémies simulées de 68% (écart interquartile (EI) 62 à 72) avec une réduction de 81% (EI 69 à 87) du nombre de cas comparée à une épidémie non contrôlée. Ces interventions réduisent d'un facteur de 44 (EI 27 à 78) le nombre de personnes ciblées pour éviter un seul cas de choléra, comparé à des campagnes de masse dans des zones à haut risque. Le rayon optimal de ciblage autour des ménages des cas varie selon les interventions, avec un rayon allant de 30 à 45m pour les antibiotiques, et de 70 à 100 m pour

la vaccination orale et le traitement de l'eau au point d'utilisation. Des CATIs combinées, alliant le traitement de l'eau ou la prophylaxie antibiotique avec la vaccination orale, ont montrées une augmentation limitée de l'impacte des interventions. La riposte par vaccination et traitement de l'eau dans un rayon de 100 m autour des ménages de cas et tôt dans l'épidémie réduit la durée de l'épidémie de 70% (EI 65 à 75) et le nombre de cas de 82% (EI 71 à 88) comparé à une épidémie non contrôlée. Les interventions tardives, même après le pic épidémique, réduisent relativement peu le nombre de cas évités, mais raccourcissent la durée de l'épidémie, par exemple de 28% (EI 15 to 45) en vaccinant dans un rayon de 100 m. Bien que cette étude ait suivi une approche rigoureuse et basée sur des données provenant d'une épidémie réelle, l'incertitude relative de l'impact du traitement de l'eau et la prophylaxie antibiotique sur la réduction des risques d'infection, ainsi que l'hétérogénéité de la dynamique des épidémies de choléra selon leur localisation, limite la précision et la généralisation de nos résultats quantitatifs.

Conclusions

Dans cette étude, nous avons montré que des interventions de riposte combinant la vaccination orale ciblée, le traitement de l'eau au point d'utilisation et la prophylaxie antibiotique dans un rayon adapté autour des ménages des cas déclarés sont des méthodes efficaces et efficientes de lutte en cas d'épidémie de choléra. Ces méthodes peuvent être efficaces et complémentaires à des campagnes de masse, particulièrement lors de la phase initiale d'une flambée épidémique en situation de faibles ressources de riposte, ou lors d'une épidémie en déclin pour en raccourcir la durée totale.
